# The Length of Haplotype Blocks and Signals of Structural Variation in Reconstructed Genealogies

**DOI:** 10.1093/molbev/msaf190

**Published:** 2025-08-06

**Authors:** Anastasia Ignatieva, Martina Favero, Jere Koskela, Jaromir Sant, Simon R Myers

**Affiliations:** Department of Statistics, University of Oxford, Oxford, UK; School of Mathematics and Statistics, University of Glasgow, Glasgow, UK; Department of Mathematics, University of Stockholm, Stockholm, Sweden; Department of Statistics, University of Warwick, Coventry, UK; School of Mathematics, Statistics and Physics, Newcastle University, Newcastle, UK; Department of Statistics, University of Oxford, Oxford, UK; Department of Statistics, University of Oxford, Oxford, UK

## Abstract

Recent breakthroughs have enabled the accurate inference of large-scale genealogies. Through modelling the impact of recombination on the correlation structure between genealogical local trees, we evaluate how this structure is reconstructed by leading approaches. Despite identifying pervasive biases, we show that applying a simple correction recovers the desired distributions for one algorithm, Relate. We develop a statistical test to identify clades spanning unexpectedly long genomic regions, likely reflecting regional suppression of recombination in some individuals. Our approach allows a systematic scan for inter-individual recombination rate variation at an intermediate scale, between genome-wide differences and individual hotspots. Using genealogies reconstructed with Relate for 2,504 human genomes, we identify 50 regions possessing clades with unexpectedly long genomic spans ($P<1\cdot 10^{-12}$). The strongest signal corresponds to a known inversion on chromosome 17. The second strongest uncovers a novel 760-kb inversion on chromosome 10, common (21%) in S. Asians and correlated with GWAS hits for a range of phenotypes. Other regions indicate additional genomic rearrangements: inversions (8), copy number changes (2), or other variants (12). The remaining regions appear to reflect recombination suppression by previously unevidenced mechanisms. They are enriched for precisely spanning single genes ($P=5\cdot 10^{-10}$), specifically those expressed in male gametogenesis, and for eQTLs ($P=2\cdot 10^{-3}$). This suggests an extension of previously hypothesized crossover suppression within meiotic genes, towards a model of suppression varying across individuals with different expression levels. Our methods can be readily applied to other species, showing that genealogies offer previously untapped potential to study structural variation and other phenomena impacting evolution.

## Introduction

In the presence of recombination, the genealogical history of a sample can be fully captured in the form of an ancestral recombination graph (ARG). This can be represented as a sequence of local trees describing the sample genealogy at each locus, connected by recombination events that reshape these trees along the genome. ARGs can, in principle, capture the effects of all the evolutionary forces that have shaped the observed genetic diversity of a sample of sequences, while providing a much more efficient representation of genomes than multiple sequence alignments (Kelleher et al. 2019). Thus, statistical inference methods which take ARGs as inputs have the potential to provide very powerful insights into evolutionary events and parameters.

However, the true underlying genealogy is usually not observable in practice, and the ARG must be reconstructed from the data, which typically comprises a set of genetic sequences sampled at the present time. This is a notoriously difficult problem, due to the computational cost of traversing the huge search space of plausible ARGs. This has been the main bottleneck for the widespread development of genealogy-based inference, but has seen impressive recent breakthroughs, with methods now capable of reconstructing and efficiently storing ARGs for tens or even hundreds of thousands of samples (Speidel et al. 2019; Wohns et al. 2022; Zhan et al. 2023; Zhang et al. 2023). Inference using ARGs reconstructed from large-scale human sequencing datasets is in its infancy, but has already produced novel scientific insights, for instance in untangling the history of human demography (Speidel et al. 2019; Wohns et al. 2022) and understanding the phenotypic effects of genetic variants (Zhang et al. 2023).

This is a rapidly developing field, however progress has been hampered by the fact that methods which work very well on simulated ARGs often lose power and accuracy when applied to reconstructed genealogies, for reasons that are, in general, poorly understood. Methods for quantifying the quality of ARG reconstruction have generally been limited to comparing simulated to reconstructed ARGs, to quantify how well local tree topology (Kelleher et al. 2019; Speidel et al. 2019) and pairwise coalescence times (Brandt et al. 2022) are recovered. Deng et al. (2021) and McKenzie and Eaton (2022) took the approach of deriving the distribution of the genomic distance between consecutive local trees (under a given model), and comparing this to the empirical distributions calculated from reconstructed ARGs. All of these studies have broadly demonstrated that different tools have somewhat different strengths, but since they commonly output strikingly different ARGs for the same dataset (Wong et al. 2024), more in-depth exploration is needed to understand (and correct) the underlying causes.

Moreover, all currently available methods of recording and inferring ARGs typically only account for mutations in the form of single base substitutions, and ignore the presence of genomic structural variants (SVs), such as duplications and inversions. SVs are a ubiquitous and evolutionarily important type of mutation, playing a key role in speciation and local adaptation (Kirkpatrick and Barton 2006), and within human populations through altering the structure and expression of genes (Chiang et al. 2017; Abel et al. 2020). Thus, identifying and analyzing the evolution of structural variants at all scales is an incredibly important goal, but so far no studies have attempted to leverage ARGs for this purpose, beyond simulation (e.g. Peischl et al. 2013).

**Fig. 1.**
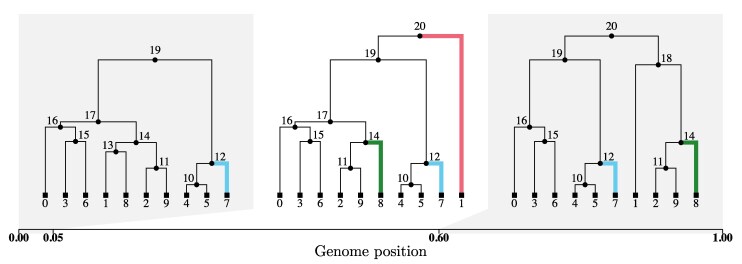
An ARG with n=10 sequences, represented as a sequence of the corresponding local trees along the genome. The *time-length* of an edge is given by the time of its parent node less the time of its child node; *older* edges are those closer to the root of a local tree. Edge highlighted in red (1→20) spans one local tree and has span 0.55; edge highlighted in green (8→14) spans two local trees and has span 0.95; edge highlighted in blue (7→12) spans all three local trees and has span 1. ARG simulated using msprime and visualized using tskit (Kelleher et al. 2016; Baumdicker et al. 2022).

Within an ARG, each edge has a well-defined genomic span during which it is present in the local trees (Fig. 1). We analytically derive the theoretical distribution of this quantity using the well-known SMC model (Marjoram and Wall 2006), which is an excellent approximation to the coalescent with recombination (Griffiths and Marjoram 1997). Through calculating the empirical distribution of edge span in ARGs reconstructed by ARGweaver, Relate, tsinfer and tsdate, and ARG-Needle in simulation studies, we find that these tools recover the correct distribution to varying degrees of success.

We then derive the distribution of the length of a haplotype block within an ARG, defined as the genomic span of a given clade of samples (as by Shipilina et al. 2023). Recombination is suppressed in individuals heterozygous for an inversion, which manifests in the ARG as a clade of samples that persists for a longer stretch of the genome than would otherwise be expected: we use this idea to construct a computational tool for detecting localized (between-clade) suppression of recombination (DoLoReS: Detection of Localized Recombination Suppression). This tool is tailored for use with Relate ARGs, implementing suitable adjustments to correct for possible sequencing and phasing errors in the data, and the particularities of the ARG reconstruction method. We demonstrate the power and accuracy of this tool for both simulated and reconstructed ARGs.

Finally, we apply DoLoReS to an ARG for the 1000 Genomes Project (1KGP; 1000 Genomes Project Consortium 2015) reconstructed using Relate (Speidel et al. 2019). We detect several known inversions: for instance, one of the top significant hits is the 17q21.31 inversion polymorphism common in European populations (Stefansson et al. 2005). The second strongest signal corresponds to a previously unknown 760-kb inversion on 10q22.3, which we validate using data from the Human Pangenome Reference Consortium (HPRC; Liao et al. 2023); this inversion is common in S. Asian populations (with a frequency of 21%), spans a number of genes associated with lung function, and correlates with a number of GWAS hits for hematological and immunological traits. We find several other new SVs, and show that our method also detects distinguishable signals of other structural variants, particularly copy number variants (CNVs) and complex rearrangements. This demonstrates that, while Relate only uses SNP data and does not explicitly infer or account for SVs, the reconstructed ARGs still capture the signal of SV presence.

Our study thus presents new results on the SMC’ model by characterizing the distribution of genomic spans of edges and clades, and also demonstrates that these are very close to the equivalent distributions under the coalescent with recombination. This adds to earlier work demonstrating the quality of the SMC’ approximation (Hobolth and Jensen 2014; Wilton et al. 2015) and using it to derive various quantities and distributions of interest (Eriksson et al. 2009; Harris and Nielsen 2013; Carmi et al. 2014; Deng et al. 2021; McKenzie and Eaton 2022). From the point of view of detecting inversions, Bansal et al. (2007) looked for SNPs in long-range LD, and our study builds on this by constructing a genealogy-based statistical test for whether such LD is more extreme than expected. This also links to other work focused on detecting inversions through disruptions in LD patterns, such as Kemppainen et al. (2015) and Li and Ralph (2019). Finally, we note the connections to the work of Peischl et al. (2013), who modelled inversions under the SMC by modifying the rates at which lineages designated as carriers and non-carriers can coalesce in local trees (and analyzed this model through simulation).

Code implementing the methods and used to produce the figures is publicly available at github.com/a-ignatieva/dolores.

## Results

### The Probability that an Edge is Broken up by the Next Recombination Event Along the Genome is Biased in Reconstructed ARGs, Particularly for Old Edges

For a given edge of the ARG, its span can be defined as the genomic positions where it is present in the corresponding local trees (as illustrated in Fig. 1); this is determined by recombination events that change local tree topologies and coalescence times. Intuitively, the longer (resp. shorter) the *time-length* of an edge, the more (resp. less) likely it is to be broken up by recombination as we move along the genome, so its *span* along the genome should be shorter (resp. longer). This is a fundamental property of the ARG which has several implications. For instance, in the absence of recombination, the genealogy is a single tree with all edge spans equal to the length of the genome, and the probability that a given edge carries at least one mutation is proportional to its time-length (assuming mutations occur as a Poisson process along the edges). On the other hand, in the presence of recombination, the number of mutations per edge is less variable, because of the interplay between edge time-length and span.

The SMC’ is an approximation to the coalescent with recombination (CwR) model characterizing the distribution over sample genealogies. Under this model, an ARG can be simulated by starting with an initial binary tree $T_{1}$, representing the sample genealogy at the leftmost point of the chromosome. The distance until the next recombination breakpoint along the genome is then drawn from an exponential distribution, with rate $L_{T_{1}}\cdot \rho /2$, where *ρ* is the population-scaled recombination rate, and $L_{T_{1}}$ is the total branch length of $T_{1}$. Then a recombination point is chosen uniformly at random along the edges of $T_{1}$, the subtree underneath this point is allowed to coalesce at a new location (with the usual coalescent dynamics), and the result is the next local tree $T_{2}$. This process is repeated until the end of the chromosome is reached (see SI, supplementary Sections S1.1 and S1.2, Supplementary Material online, for full details).

**Fig. 2.**
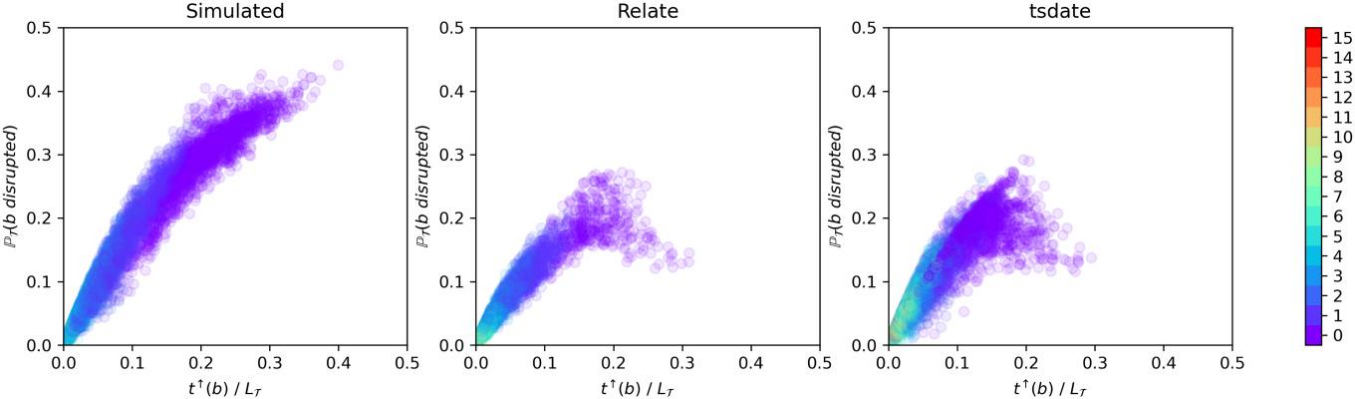
Value of $P_{T}(b\;\text{disrupted})$ calculated for each edge *b*, against the time at its upper endpoint $t^{↑}(b)$ divided by the total branch length of the tree, $L_{T}$. Left panel: for a uniform random sample of 10,000 edges in one simulated ARG with n=100 samples (dataset 1 parameters given in Methods, Section 4.6.1). Middle and right: same quantity calculated for each edge of the ARG reconstructed from the simulated data using Relate (middle) and tsinfer/tsdate (right). Colour shows number of edges between the top end of the edge and the root of T (purple dots correspond to edges extending from the MRCA node).

We first calculate analytically, under the SMC’, the probability $P_{T}(b\;\text{disrupted})$ that a given edge *b* in a local tree T is disrupted by the next recombination event along the genome (Methods, Section 4.1). This probability does not have a simple form, but can be calculated exactly for a given edge and local tree. The left panel of Fig. 2 shows $P_{T}(b\;\text{disrupted})$ calculated for each edge in an ARG simulated under the SMC’, against the normalized age of the edge. Middle and right panels show the same quantities, but calculated for each edge of an ARG reconstructed from the simulated data: using Relate (middle), and tsinfer/tsdate (right).

For the simulated ARG, the probability that a given edge is disrupted by a recombination event is higher for older edges. This is as expected, as edges at the top of a local tree tend to have greater time-length and co-exist with fewer other lineages. This makes such edges more likely to be quickly broken up by recombination, since (i) the rate of recombination on the edge is relatively higher, due to its greater time-length, and (ii) if a recombination event happens below the edge, and the recombinant lineage has not yet coalesced by the time at the lower end of the edge, it is likely to disrupt the edge since it is one of the few remaining choices for coalescence.

However, old edges are generally difficult to accurately reconstruct using the sequences at the leaves due to lack of signal, unless they are strongly supported by mutations (which implies longer edge span along the genome). As a result, reconstructed ARGs have fewer old edges (with fewer points lying towards the right of the plots), and the old edges that *are* present tend to have lower probability of disruption than those of a similar age in the simulated ARG. This bias is seen for both Relate and tsinfer/tsdate. This demonstrates explicitly the difficulty with faithfully reconstructing the ARG topology and event times in the deep past.

### The Distribution of Edge Span is Recovered with Varying Accuracy by different ARG Reconstruction Methods

We next derive an approximation to the distribution of edge span along the genome. If an edge *b* first appears at a position of the genome where the corresponding local tree is T, we show that the waiting distance along the genome until is it broken up by a recombination event is approximately exponentially distributed as


(1) $$\text{Exp}\left(P_{T}(b\;\text{disrupted})\cdot L_{T}\cdot \rho /2\right),$$

we also derive an equivalent approximation for the case where the recombination rate varies along the genome (Methods, Section 4.2). Given a simulated or reconstructed ARG, we can thus calculate a corresponding *P*-value for each edge under this model (assuming edges have independent exponentially distributed spans), and check if the *P*-values follow the expected (uniform) distribution using a Q–Q plot (SI, supplementary Section S1.8, Supplementary Material online).

**Fig. 3.**
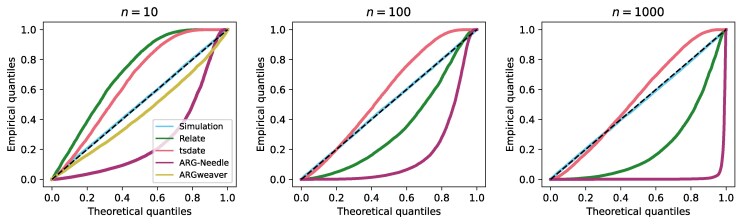
Q–Q plots for edge spans in ARGs simulated with parameters of dataset 1 (dataset 2 for ARGweaver) given in Methods, Section 4.6.1, with n=10 (left panel), n=100 (middle panel), n=1,000 (right panel). Dashed line: diagonal from (0,0) to (1,1). ARGweaver results only shown for n=10 due to excessive runtimes for larger sample sizes. Calculated for a random sample of 10,000 edges for each ARG. An overabundance of edges with short (resp. long) spans would drag the points below (resp. above) the diagonal.

We apply this to ARGs reconstructed from simulated data using Relate, tsinfer/tsdate, ARG-Needle, and ARGweaver. The resulting Q–Q plots are shown in Fig. 3 (the corresponding histograms are also shown in SI, supplementary fig. S13, Supplementary Material online). For ARGs reconstructed using Relate and tsinfer/tsdate, we adjust for the fact that these tools do not detect recombination events that affect only edge time-lengths (SI, supplementary Section S1.8.1, Supplementary Material online). Moreover, we adjust for the fact that Relate does not attempt to infer edge span when the edge is not supported by at least one mutation (SI, supplementary Section S1.8.2, Supplementary Material online), in which case the local topology is resampled from one local tree to the next (unlike tsinfer, which more directly captures the span of edges in the reconstructed ARG based on shared ancestry).

ARGweaver accurately captures the edge span distribution, with the small deviation from the diagonal as the simulation used the SMC’ while ARGweaver uses the SMC (SI, supplementary Section S1.12, Supplementary Material online), although these results were only calculated for n=10 due to excessive runtimes for larger sample sizes. Note that while it is possible to use the SMC’ model with the latest version of ARGweaver, we found that the resulting ARGs contained cycles, which prevented us from calculating the required probabilities.

ARGs reconstructed by tsinfer/tsdate consistently have an excess of edges with long spans, while for Relate this depends on the sample size; both tools however produce skewed distributions of edge span. This is likely to be, in part, due to the waiting distances between trees being generally skewed, as shown by Deng et al. (2021). Moreover, the ARGs produced by tsinfer contain polytomies (nodes with more than two children), which is also not accounted for under the SMC’ model. This results in the total branch length of a reconstructed local tree to often be greater than it would be if the polytomies were resolved to make the tree binary. In addition, some recombination events that would disrupt the edge under the SMC’ (such as recombination points located on child edges), may not do so when the tree contains polytomies.

Relate first reconstructs the sequence of (correlated) local trees along the entire genome and then calculates edge spans, using an argument based on the similarity of clades subtended by the edge in successive trees. Thus, edge spans are calculated approximately, rather than being explicitly inferred, which we do not account for.

The threading procedure used by ARG-Needle to reconstruct the ARG uses the ASMC model (Palamara et al. 2018) to estimate coalescence times between the added sequence and the closely-related samples already in the ARG, with the lowest possible resulting coalescence time dictating which edge the sequence is threaded to and over what genomic length. This procedure (which relies on a combination of maximum a posteriori and posterior mean estimates for the coalescence times) is optimized for metrics having a direct effect on downstream analyses, and appears to lead to ARG-Needle consistently underestimating edge spans. We note that this is somewhat affected by the choice of time discretisation used within the ASMC (particularly for small sample sizes), but our overall findings do not change significantly when toggling this parameter.

Considering the unconditional distributions of the edge spans in the simulated and reconstructed ARGs (i.e. calculating the observed span of each edge and plotting the overall histogram) results in similar conclusions (SI, supplementary fig. S15, Supplementary Material online). Further, histograms of the expected number of mutations per edge (being the product of the observed edge span, observed time-length, and the mutation rate) demonstrate large deviations between the distributions for simulated and reconstructed ARGs (SI, supplementary fig. S16, Supplementary Material online).

### The Distribution of the Length of a Haplotype Block is Recovered Well by Relate after a Suitable Correction

A clade *G* of an ARG can be defined through the set of samples it contains, by writing $G=(g_{1},g_{2},\ldots ,g_{n})$, where $g_{i}=1$ if sample *i* is in *G*, and 0 otherwise. The genomic span of *G* is defined as the interval [a,b], where *a* is the leftmost position at which the corresponding local tree has a branch subtending exactly the samples in *G*, and *b* is the rightmost such position. For instance, in Fig. 1, the clade G=(1,0,1,1,0,0,1,0,1,1) (containing samples 0, 2, 3, 6, 8, 9) has a genomic span of 0.55, since *G* first appears at position 0.05 and is broken up by the following recombination event at position 0.60.

Using the SMC’ model, we derive the distribution of the genomic span of a given clade *G*, conditional on the local tree T in which it first appears (Methods, Section 4.3). This is approximately exponentially distributed as


$$\text{Exp}\left(P_{T}(G\;\text{disrupted})\cdot L_{T}\cdot \rho /2\right),$$

where $P_{T}(G\;\text{disrupted})$ is the probability that *G* either gains or loses at least one sample following the next recombination event along the genome. We also derive an equivalent result when the recombination rate varies along the genome. Since our definition of a clade is equivalent to that of a haplotype block (Shipilina et al. 2023), this distribution is that of the length of a haplotype block. It is important to emphasise that since we condition on *G* and the local tree in which it first appears, this distribution is conditional on the size and age of *G* (as well as the local tree topology and event times, and the recombination map), rather than averaged over all clades.

**Fig. 4.**
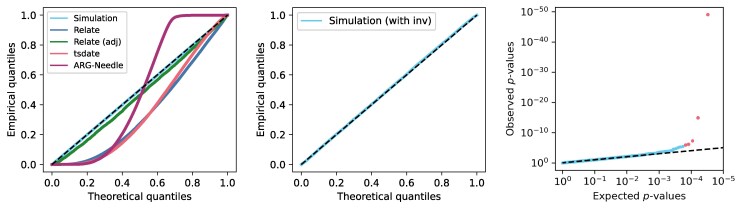
Left: Q–Q plot for clade *P*-values in simulated and reconstructed ARGs with n=100 and parameters as for dataset 1 given in Section 4.6.1. An overabundance of clades with long spans would drag the points below the diagonal. Middle and right: Q–Q plot and *P*-value plot for ARG simulated using SLiM with one inversion under balancing selection (red points correspond to clades with *P*-value below the Bonferroni-corrected significance threshold; blue points correspond to clades with non-significant *P*-values).

For a given (simulated or reconstructed) ARG, we can thus calculate a corresponding *P*-value for each observed clade under this model, and again check if these follow the expected uniform distribution using a Q–Q plot. Figure 4 (left panel) shows that the approximation provides an excellent fit for an ARG simulated under the SMC’ (blue points). Clade spans in ARGs reconstructed using tsinfer/tsdate (red points) tend to be over-estimated in general (possibly due to the presence of polytomies), while ARG-Needle (purple points) both under- and over-estimates this quantity. Relate (dark blue points) also tends to over-estimate clade spans; however, through analyzing and correcting the causes of this, we propose a correction which effectively removes this bias (green points). Firstly, a clade might only be supported by mutations intermittently along its span, and between these regions Relate does not attempt to keep the clade intact. This causes some clade spans to be too short, and we correct this by extending the calculated span of a clade, if the clade disappears and subsequently reappears within a given distance (which is the cM_limit input parameter to DoLoReS, which defaults to 1cM and should be chosen to be relatively large as explained in SI, supplementary Section S1.13.1, Supplementary Material online). Secondly, since it is difficult to reconstruct the endpoints of clades exactly, which may results in genomic span being overestimated, we use the leftmost and rightmost mutations that support a given clade to calculate its genomic span. See SI, supplementary Section S1.13.1, Supplementary Material online for full details.

### Simulated and Reconstructed ARGs Capture Signals of Structural Variation

Chromosomal inversions are a type of structural variant, whereby as a result of recombination, the genome breaks at two points and the segment between the breakpoints is reinserted in the opposite orientation. While recombination can proceed normally in individuals homozygous for the inversion, in heterozygotes recombination is substantially suppressed in the region containing the inversion, since a crossover recombination within the region is likely to result in the production of unbalanced gametes (Kirkpatrick 2010). Detecting inversions computationally from sequencing data typically relies on paired-end mapping (detecting reads mapping in the opposite orientation to the reference), split-read methods (detecting reads that map onto the reference with gaps), and *de novo* assembly (directly reconstructing the sequenced genome to look for differences with the reference) (Tattini et al. 2015). For instance, DELLY (Rausch et al. 2012) implements a combination of these approaches and was used to identify hundreds of inversions in 1KGP data (Sudmant et al. 2015). In general, however, such methods suffer from high false positive rates and poor sensitivity, with their performance depending on the size of the inverted region, particularly for short-read sequencing data (Lucas Lledó and Cáceres 2013); the detection of structural variants from long-read data is challenging due to high sequencing error rates (Sedlazeck et al. 2018). Inversions can also be detected by looking for disrupted patterns of linkage disequilibrium (LD) using population data (Bansal et al. 2007; Kemppainen et al. 2015; Li and Ralph 2019), however this is sensitive to noise in the LD patterns and cannot be used to reliably detect inversions that are not large and high-frequency.

Suppose that in a given ARG, an inversion happens on an edge *g* which subtends a clade *G*. Suppression of recombination in heterozygotes implies that if a lineage within *G* undergoes a recombination, it will coalesce with lineages in *G* with high probability; likewise, if a recombination event happens on an edge not carrying the inversion, with high probability it will coalesce with edges outside *G* (SI, supplementary fig. S3, Supplementary Material online). This implies that inversions can be detected by looking for clades that last for “too long” along the genome due to this local suppression of between-clade recombination. Note that the effect of an inversion differs from simple suppression or general regions of low recombination, since recombination is suppressed in a clade-specific way.

Note that we are imposing the simplifying assumption that recombination in heterozygotes is suppressed completely in the inverted region (so the clade *G* remains completely intact). In reality, recombination can occur in heterozygotes: multiple crossovers occurring in the inverted region would enable this, but such events have relatively low probability unless the inversion region is very large or the recombination rate is very high. Localized reshuffling of clades can also arise through gene conversion, which can indeed be at least as frequent within inversions as outside (Crown et al. 2018; Korunes and Noor 2019), and for reconstructed ARGs we implement a correction to account for this when calculating the genomic span of a clade (described in SI, supplementary Section S1.13.1, Supplementary Material online).

**Test 1:** Phrasing the above as a hypothesis test, for each clade we calculate its genomic span [a,b], and calculate a *P*-value as the probability of this clade having a span greater than b−a (Methods, Section 4.3.1). Simulation studies confirm that for ARGs simulated under the SMC’ model without inversions, these *P*-values are approximately uniformly distributed (Fig. 4, left panel, blue points), as expected.

**Test 2:** Alternatively, we can estimate the number of recombination events *R* that occurred within the genomic interval [a,b], and calculate a *P*-value as the probability that *G* stays intact after at least *R* recombination events (Methods, Section 4.3.2). This is exactly equivalent to Test 1 when the ground truth ARG and recombination map are known. For reconstructed ARGs, we apply both of these tests since they are susceptible to false positives in different practical scenarios: Test 1 is sensitive to the choice of recombination map and presence of sequencing gaps, while Test 2 can result in false positives if there is a high level of recurrent mutation (which can cause reconstruction errors).

Both of these tests are implemented in DoLoReS, which outputs calculated genomic spans and other characteristics of each clade within an input ARG (in tskit format) and the corresponding *P*-values.

#### Simulated ARGs

**Fig. 5.**
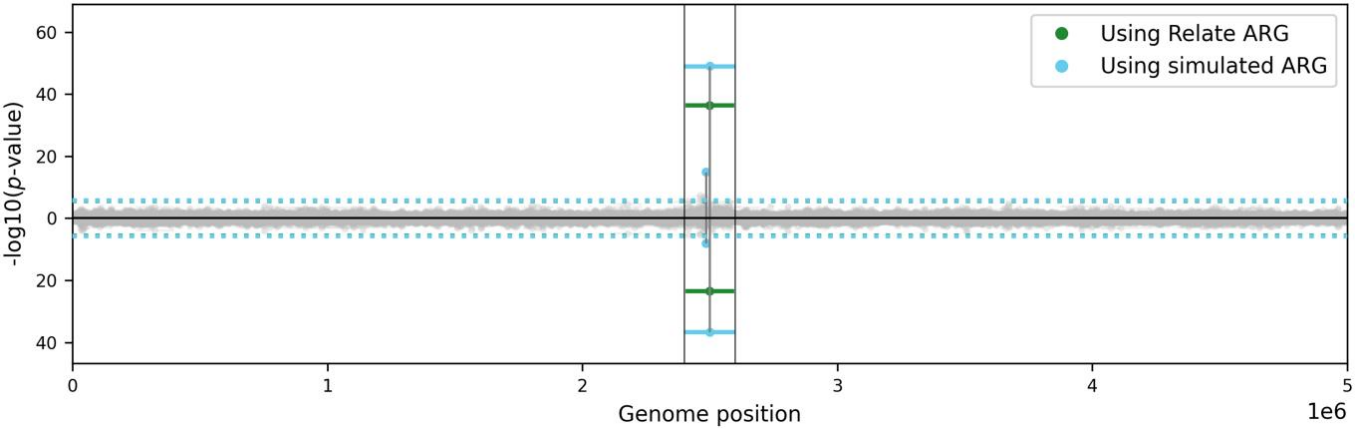
*P*-values for clades in simulated ARG and ARG reconstructed using Relate. Horizontal lines show the genomic span of each clade (with midpoint marked by circle); those with non-significant *P*-values are shown in grey, otherwise in blue and green for the simulated and Relate ARG, respectively. Corresponding *P*-values using Test 1 and Test 2 are shown on the *y*-axis above and below 0, respectively. Vertical black lines delineate the region of the inversion; dotted blue (resp. green) horizontal line shows Bonferroni-corrected significance threshold for calculations using the simulated (resp. Relate) ARG (note these overlap very closely).

To demonstrate the power of these tests in detecting inversions, we used SLiM (Haller and Messer 2019; Haller et al. 2019) to simulate an ARG with one 200-kb inversion (Methods, Section 4.6.2), under balancing selection, since this is a common mechanism under which polymorphic inversions are maintained in different species (Wellenreuther and Bernatchez 2018). Figure 4 shows the corresponding Q–Q plot (middle panel) and *P*-values for each clade (right panel) using Test 1. The spans of most clades are unaffected by the inversion, so most of the points on the Q–Q plot adhere tightly to the diagonal. However, there are outliers in the tail (right panel, shown in red) with significant *P*-values (after Bonferroni correction for multiple testing). Figure 5 shows the *P*-values for each clade using Test 1 (above the 0 line) and Test 2 (below the 0 line). The clades with significant *P*-values (using a Bonferroni-corrected significance threshold of $2\cdot 10^{-6}$) overlap the location of the inverted region, and the clade subtended by the edge carrying the inversion is a significant outlier with the lowest *P*-value. Repeating the simulation using SLiM with no inversion (and otherwise the same parameters), the *P*-values are approximately uniformly distributed and there are no clades with significant *P*-values (SI, supplementary fig. S17, Supplementary Material online).

#### Reconstructed ARGs

We next applied DoLoReS to an ARG reconstructed using Relate for the data simulated using SLiM as described above (applying the correction described in Section 2.3). We detect one clade which has significant *P*-values using both tests, shown in green in Fig. 5, which is exactly the clade carrying the simulated inversion. The corresponding Q–Q and *P*-value plots for Test 1 are shown in the top row of SI, supplementary fig. S18, Supplementary Material online, showing that the Q–Q plot very close to the diagonal. The equivalent plots for a simulation with no inversion are shown in the bottom row, demonstrating that the *P*-values are approximately uniformly distributed and there are no false positives.

We performed further simulation studies to evaluate performance, simulating 100 replicates each for inversions with size varying between 0 and 200 kb (and otherwise the same parameters as above), as described in SI, supplementary Section S1.13.2, Supplementary Material online. The resulting ROC curves (SI, supplementary figs. S8 and S9, Supplementary Material online) show excellent performance when using simulated ARGs, and that high sensitivity is maintained for ARGs reconstructed using Relate for inversions of 100 kb or longer. While our theoretical results hold for clades of any size greater than one, we note that power to detect inversions drops as inversion frequency decreases, since smaller clades have shorter expected spans (so it becomes more difficult to detect outliers).

We also evaluated the performance of our method in predicting inversion genotypes, by considering the samples within the detected significant clades, and compared this against invClust (Cáceres and González 2015), an inversion detection method based on clustering haplotypes using multidimensional scaling. As described in SI, supplementary Section S1.13.3, Supplementary Material online, we found that our method outperforms invClust (SI, supplementary fig. S10, Supplementary Material online), while, unlike invClust, not requiring candidate inverted regions as input. We also found that DoLoReS is accurate in predicting the position of the inverted region (SI, supplementary fig. S10, Supplementary Material online), with the predicted region overlapping over half of the true region 81% of the time.

### Structural Variants can be Detected using ARGs Reconstructed from 1 KGP Data

**Fig. 6.**
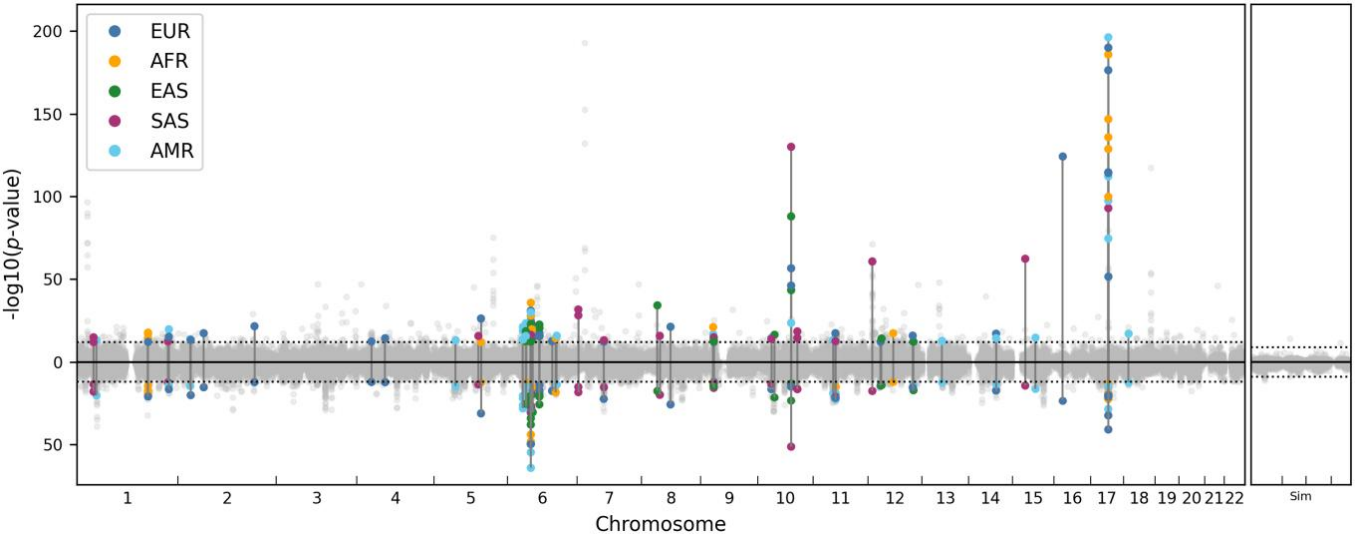
Left panel: *P*-values for clades in 1KGP ARG reconstructed using Relate. Each *P*-value shown as a point, with position on *x*-axis being the midpoint of the corresponding clade span; *P*-values using Test 1 and Test 2 are shown on the *y*-axis above and below 0, respectively. Points for clades with significant *P*-values shown in colour corresponding to the super-population (legend); corresponding *P*-values for Test 1 and Test 2 for each significant clade are connected by solid vertical lines. Dotted black horizontal lines show Bonferroni-corrected significance threshold of $1\cdot 10^{-12}$. Right panel: results for reconstructed ARG using simulated data, dotted line shows Bonferroni-corrected significance threshold of $1\cdot 10^{-9}$ (Methods, Section 4.6.3).

We applied DoLoReS to an ARG reconstructed using Relate for the 1KGP (Phase 3) data by Speidel et al. (2019), splitting the ARG into the five super-populations to avoid confounding by population structure, accounting for varying population size through time, and applying several filters to correct for possible sequencing errors and artifacts, which also corrects for phasing errors and the presence of gene conversion (Methods, Section 4.4). The resulting *P*-values are shown in Fig. 6 (and SI, supplementary figs. S19 and S20, Supplementary Material online). There are a total of 125 significant clades, clustering into 50 localized regions.

#### 17q21.31 Inversion

One of the detected regions with the lowest *P*-values, on chromosome 17, corresponds to the known 900-kb inversion common in European populations, with two distinct haplotypes H1 and H2, corresponding to inversion non-carriers and carriers, respectively (Stefansson et al. 2005). Since we separately test each clade within each of the population-specific ARGs, multiple clades from the same population can have significant *P*-values (creating the vertical stack of points seen in Fig. 6). This is because if the samples are perfectly split into two clades *A* and *B* (of carriers and non-carriers of the inversion, respectively), both will show detectable signal of between-clade recombination suppression. Moreover, sub-clades of *A* or *B* can also have longer genomic spans due to the effects of locally suppressed recombination.

**Fig. 7.**
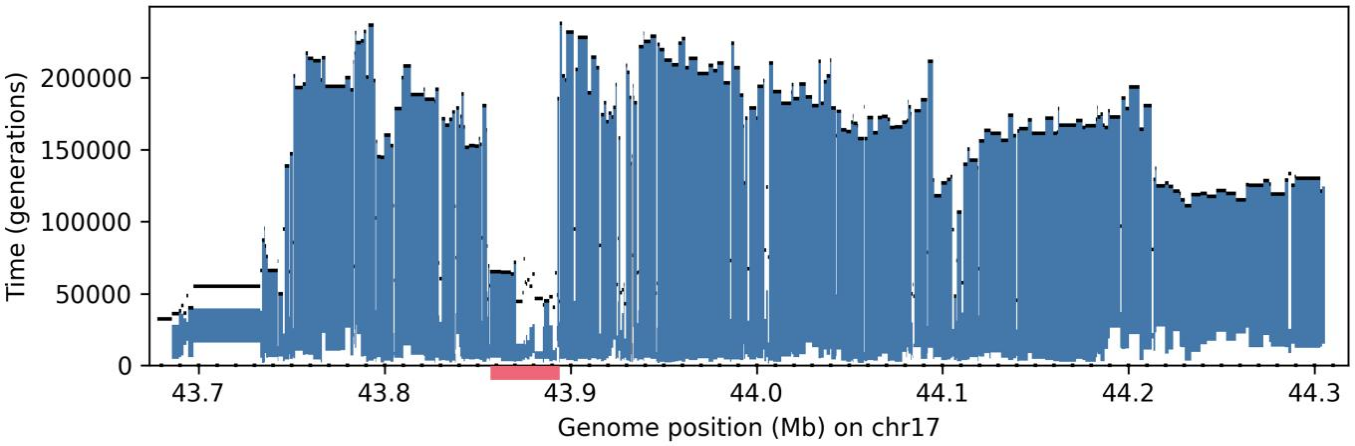
Age estimate for 17q21.31 inversion (the H2 haplotype) using the 1KGP ARG subsetted to European populations. Vertical lines at each genomic position are drawn between the time at the lower and upper end of the branch in the corresponding local tree which subtends the predicted carriers of the inversion. Black lines show MRCA time in full (all-population) ARG. Time is measured in generations, genome positions given in GRCh37 coordinates. Red bar highlights region where inversion appears much more recent.

We detect strong signal of this inversion in all populations apart from E. Asian, estimating its average frequency at approximately 24% in European, 15% in American, 6% in S. Asian, and 2% in African populations, which aligns well with prior estimates (Donnelly et al. 2010). The ARG also allows for the estimation of the time of the inversion, through identifying the predicted clade carrying the inversion in each local tree, and extracting the time at the lower and upper end of the branch subtending this clade (Fig. 7); this gives an average age of between 8,000 and 123,000 generations. This aligns with the estimates of 3m years of Stefansson et al. (2005) and 2.3m years of Steinberg et al. (2012), but highlights a large amount of uncertainty; the inversion has also been estimated to be much younger at around 100k years by Donnelly et al. (2010). We detect a region where the inversion appears relatively much more recent (highlighted in red on Fig. 7), overlapping the 5’ UTR region of the *CRHR1* gene (SI, supplementary fig. S22, Supplementary Material online). This is the same as the region of very low sequence divergence between the H1 and H2 haplotypes found by Steinberg et al. (2012). The topology of the ARG, with H1 and H2 forming two disjoint clades with a very ancient MRCA time (SI, supplementary fig. S22K, Supplementary Material online), is consistent with the inversion being very old. From the ARG we infer a change of local tree topology and H1/H2 MRCA time within the highlighted region in Fig. 7, which is suggestive of a historic double crossover event between the two haplotypes (with the two breakpoints separated by around 40 kb), as posited by Steinberg et al. (2012) (Methods, Section 4.5.3).

We also identify signal of a CNV at around 44.3 Mb (SI, supplementary fig. S22G, Supplementary Material online), through identifying instances where for a large number of individuals (between 6 and 19 depending on genomic position), their chromosomes perfectly segregate into the same two clades (Methods, Section 4.5.2). The clades we identify correspond to individuals who are homozygous for three copies of a known 25-kb CNV at this position (which is in LD with the inversion). This demonstrates that CNVs are also detectable from reconstructed ARGs, through the signal they leave in the data that causes errors in ARG topology reconstruction.

Some significant regions correspond to other known inversions, including on 4q13.2 (Korbel et al. 2007), 11p11.12 (Porubsky et al. 2022), and a possible pericentromeric inversion on chromosome 6 (Martínez-Fundichely et al. 2014).

#### 16p12.2 Complex Structural Polymorphism

The significant clade on chromosome 16 corresponds to a known 1.1 Mb structural polymorphism, which was posited to be an inversion in a number of studies (Tuzun et al. 2005; Bansal et al. 2007). This was subsequently shown to be the result of mis-assembly of the reference genome due to complex structural variation in this region, with two common haplotypes that differ by a large (333 kb) segmental duplication (Antonacci et al. 2010). Our method thus captures signal of local recombination suppression in individuals heterozygous for this polymorphism.

Other significant hits include known regions of complex structural variation on 11q11 (Korbel et al. 2007) and 15q13.3 (Antonacci et al. 2010).

#### Structural Variation on Chromosome 6

There is a large number of significant hits on chromosome 6, with a total of 43 significant clades, clustering into 10 distinct regions (SI, supplementary fig. S19, Supplementary Material online).

We highlight the top significant hit on 6p11.2, shown in detail in SI, supplementary fig. S25, Supplementary Material online. The detected clades span approximately 340 kb overall. However, this region contains multiple CNVs (SI, supplementary fig. S25G-H, Supplementary Material online), which are not in LD with the detected clades, but clearly cause general distortion of the reconstructed genealogies in this region (as shown by the high proportion of mutations which cannot be uniquely mapped to a branch of the local trees, SI, supplementary fig. S25C, Supplementary Material online). As a result of this complex SV landscape, the significant clades are fragmented and absent in many of the local trees within this span (e.g. tree 3 in SI, supplementary fig. S25K, Supplementary Material online). Thus, while the signal of recombination suppression is still detected by our tool (since it correctly handles the significant clades disappearing and reappearing within the region), this complexity makes it difficult to confidently assign precise SV boundaries and carrier status.

#### 10q22.3 Inversion

We detect a 760-kb region of locally suppressed recombination on chromosome 10; details of the genomic spans of the significant clades are shown in Fig. 9. This indicates an inversion with an average frequency of approximately 9% (21% in S. Asian, 15% in American, 7% in European and 2% in E. Asian populations), with an age of between 2,625 and 13,392 generations (based on the lower and upper time of the edge subtending the inversion, averaged across its span). Analysis of the genealogies in this region indicates that the non-inverted orientation (with respect to the reference genome) is ancestral.

The presence of segmental duplications (blocks of DNA larger than 1 kb and with >90% sequence similarity, occurring multiple times along the genome) can enable non-allelic homologous recombination (NAHR), a potential mechanism through which large structural polymorphisms arise (Lupski and Stankiewicz 2005). NAHR between two segmental duplications which appear in opposite orientations can, specifically, lead to an inversion of the sequence that they flank. The endpoints of the significant clades (Fig. 9a) align exactly with the positions of inverted segmental duplications of length 50 kb (Fig. 9f), supporting the possibility of an inversion in this region. We confirmed that for predicted carriers of the inversion there is an enrichment of discordantly mapped paired-end reads between these breakpoints.

**Fig. 8.**
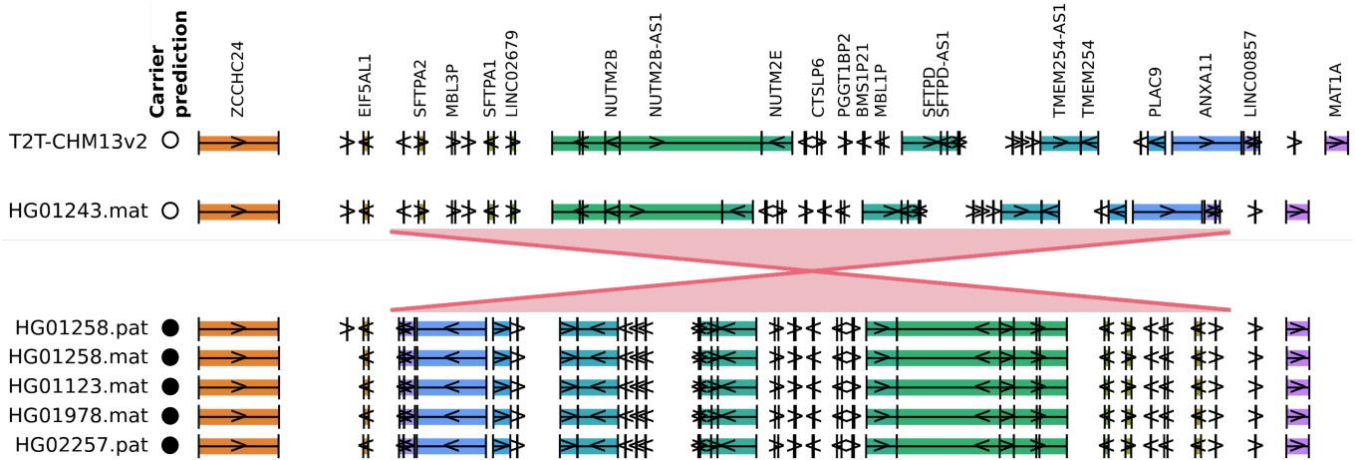
Validation of 10q22.3 inversion using HPRC data for 47 diploid individuals and the T2T-CHM13 reference. Five sequences (out of 95) display an inversion in the predicted region (shown in red), as indicated by a reversal of gene ordering compared to non-carriers (HG01243 chosen as a representative example; see SI, supplementary fig. S23, Supplementary Material online for a comparison against all sequences). Circles show predicted status of carrying the inversion (white = non-carrier, filled = carrier).

To further validate this finding, we used complete long-read sequencing data from 47 individuals generated by the HPRC (Liao et al. 2023) and the T2T-CHM13 reference (Nurk et al. 2022). The HPRC sequenced a sample of children in parent-child 1KGP trios, whereas the ARG includes the parents; we thus used tagging SNPs to determine the predicted status of each HPRC sequence. We identify five sequences carrying an inversion within the predicted region, corresponding to one homozygous (HG01258) and three heterozygous (HG01123, HG01978, HG02257) individuals (Fig. 8 and SI, supplementary fig. S23, Supplementary Material online).

**Fig. 9.**
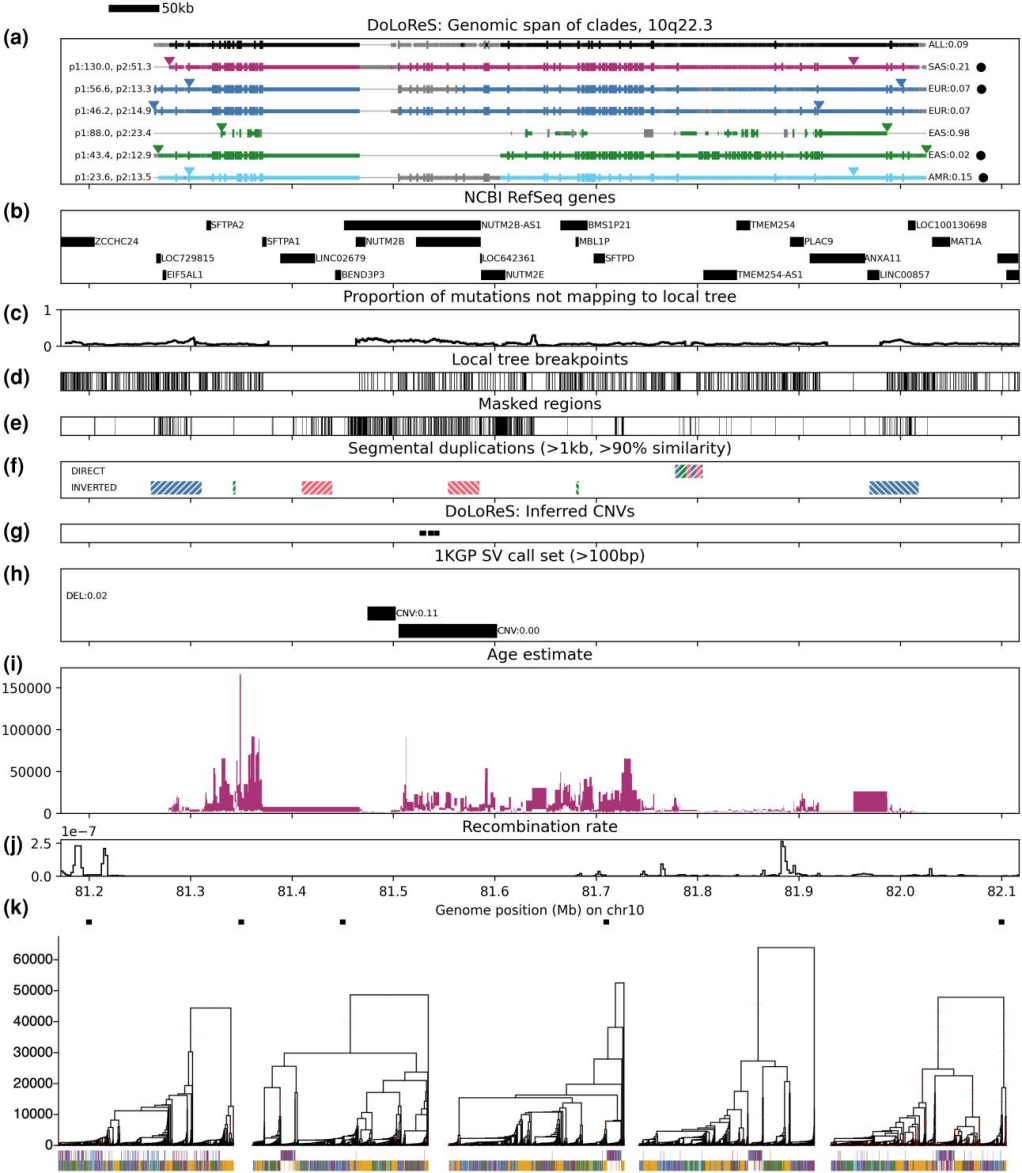
Details of 10q22.3 region. a) span of each significant clade shown as horizontal line (grey: clade is not present at that position exactly, but another highly correlated clade is). Vertical lines show positions of SNPs supporting the clade. Left label: *P*-values for Tests 1 and 2. Right label: population frequency. Black circles indicate predicted carriers. See Methods, Section 4.5 for details of this and other panels. b) positions of genes (UCSC Genome Browser NCBI RefSeq track). c) moving average of the proportion of SNPs not mapping onto the corresponding local tree. d) positions of breakpoints between local trees. e) regions masked during ARG reconstruction. f) segmental duplications. g) predicted positions of CNVs based on ARG clade analysis (Methods, Section 4.5.2). h) SVs in the 1KGP call set (labels show correlation with predicted inversion carriers). i) age of predicted inversion estimated using the ARG subsetted to S. Asian populations (Methods, Section 4.5.3). j) recombination rate (using HapMapII recombination map; averaged in bins of 2kbp). k) local trees at the positions indicated by squares (chosen at approximately equidistant points across the region, while avoiding regions with known CNVs where ARG reconstruction is unreliable); *y*-axis is time measured in generations; vertical lines drawn below each sample with colour corresponding to population (those belonging to a significant clade drawn in top row).

There are two relatively large CNVs within the span of the inversion: CNV1 at 81,474,561 bp (30 kb with zero or two copies, overall allele frequency = 0.47) and CNV2 at 81 505,304 bp (100 kb with zero or two copies, overall allele frequency = 0.02). CNV2 is not in LD with the inversion, but appears to distort the reconstructed genealogy around this region similarly to that on 6p11.2 (resulting in regions where the detected significant clades are broken up temporarily within the ARG, Fig. 9a). However, the inverted haplotype has a deletion of CNV1, with all individuals homozygous for the inversion also homozygous for zero copies of the CNV (using the 1KGP SV call set and analyzing read depth as shown in SI, supplementary fig. S24, Supplementary Material online; the deletion occurs within the *NUTM2B* gene and is also visible in Fig. 8).

The region contains a number of genes associated with lung function (pulmonary-surfactant associated proteins *SFTPA1*, *SFTPA2*, *SFTPD*, and *DYDC2*) and immunity (*ANXA11*); SNPs supporting the significant clades in these regions are significantly associated with their expression (supplementary table S1, Supplementary Material online). Searching for significant GWAS hits in LD with the predicted inversion carriers (Methods, Section 4.5.4) identified highly correlated variants associated with blood levels of SFTPD (rs2146192: $r^{2}=0.67$) and Cystatin C (rs55855057: $r^{2}=0.64$), decreased hemoglobin (rs61859980: $r^{2}=0.98$, rs61863508: $r^{2}=1.0$), decreased hematocrit (rs61859980: $r^{2}=0.98$), and increased levels of blood urea (rs36073,865: $r^{2}=1.0$, rs55838,345: $r^{2}=0.62$, rs17678,338: $r^{2}=0.6$, rs17678,338: $r^{2}=0.6$).

#### Other SVs

We scanned all of the identified significant regions for those with relatively high frequency, large genomic spans, (direct or inverted) segmental duplications near the identified breakpoints, and evidence from analysis of reads pointing to possible structural variation. This identified a total of 10 inversions (5 novel), one known deletion, one novel possible CNV or complex rearrangement, seven complex rearrangements or other variants (three novel), and five regions with strong indications of structural variation but no clear classification (two novel). For 12 of these 24 variants, we were able to confirm the presence of structural variants in these regions in the HPRC data. The remaining 26 regions show no clear evidence of structural variation. Full details are presented in Supplementary Table S1, Supplementary Material online. The identified variants include:

- a 550-kb region on 11q11 (average carrier frequency of 23%), flanked by inverted segmental duplications of length 30 kb, and overlapping a CNV from the 1KGP call set which is in LD with the predicted carriers (SI, supplementary fig. S26, Supplementary Material online). This corresponds to a known complex rearrangement identified by Korbel et al. (2007); we verified the presence of structural variation in the region in the HPRC sample. The variant overlaps a number of genes and correlates with a large number of GWAS hits for cardiovascular traits.
- a 375-kb region on 11q12.1 (200 kb away from the region described above), with an average frequency of 4%, flanked by directly oriented segmental duplications (SI, supplementary fig. S27, Supplementary Material online). Analysis of reads in this region indicates the presence of a small deletion and inversion within the predicted span correlated with the predicted carriers. This variant is in LD with a variant significantly associated with adolescent idiopathic scoliosis (rs17500359, $r^{2}=1.0$).
- a 450-kb region on 7q11.21, with an average frequency of 13%, flanked by a large number of long inverted segmental duplications (SI, supplementary fig. S28, Supplementary Material online) with large number of discordant paired-end reads correlated with predicted carriers. Although we do not find any predicted carriers in the HPRC sample, there is evidence of a 130-kb duplication within the region corresponding to a tandem duplication of *RABGEF1* (confirmed as present in 1KGP samples HG01356, HG01351 and HG01140 using analysis of reads), suggesting frequent copy number changes and rearrangements in this region.

We note that while we detect several known regions of structural variation, we do not find significant clades within the span of some other known large inversions, for instance on 8p23.1. We do not identify any clades in the reconstructed ARG that span this 4.5 Mb region. We also find that clades that are highly correlated with the predicted carriers of a tag SNP for the inversion (from Wang et al. 2023) only span short regions (at most 15 kb) and hence are not significant. Thus, our method fails to detect this inversion since the reconstructed ARG does not capture long-ranging LD within the region. We hypothesise that this might be because the probability of double crossover events within an inversion grows with its size. Our model aims to identify regions and clades with strong recombination suppression, and thus may not identify inversions that are large enough to recombine within their spans in this manner.

## Discussion

We have found that the distribution of edge span is very accurately captured by our approximation based on the SMC’. The differences between the distribution of edge spans in ARGs simulated under the SMC’ model and that in ARGs produced using reconstruction tools are due to both model misspecification and the particularities of each algorithm. Our corrections for Relate result in almost complete recovery of the theoretical distributions. We suggest that the bias seen in ARGs reconstructed using tsinfer stems from the presence of polytomies, which lead to an excess of deep edges with long spans. It is nontrivial to adjust for their presence within our model, and it is not currently possible to break polytomies at random without re-sampling edges in each tree independently (which would prevent a proper calculation of their span). In general, deep edges can arise either through true demographic events or due to inaccuracies in ARG reconstruction; our method can be used to detect deep edges that are likely to be artifactual. We note that apart from ARGweaver, none of the ARG reconstruction methods we consider are explicitly optimized to recover the distribution of edge span, focusing instead on other aspects such as node times, local tree topologies, and patterns of LD (which *are* recovered well in our simulation studies). Our results can potentially be used to improve the estimates of edge span produced by tsinfer and Relate during the topology reconstruction step, and hence also improve the downstream inference of node times.

The SMC’-based approximation we construct for the distribution of the length of a haplotype block (the span of a clade of samples) also provides a very close fit, based on simulations. The corresponding tool we develop for detecting regions of locally suppressed recombination has excellent performance on simulated ARGs, as well as (with appropriate adjustments) ARGs reconstructed using Relate. Since the method detects long clade span after adjusting for the age of the clade and the local tree topology, and hence specifically detects localized (between-clade) suppression of recombination, it has the power to discriminate inversions from other genealogy-distorting events, such as point mutations under balancing selection. Our method can be used with arbitrary models of varying population size, and (based on simulation studies) appears to be generally robust to mis-specification of demographic history. An inherent limitation of the method is that deep, ancient, population structure can result in a similar signal of localized recombination suppression as SVs, so cannot be easily distinguished. Local adaptation in the face of gene flow at individual loci (“islands of divergence”) can also result in such signals: local adaptation causes stratification at one locus but not another, resulting in clades that span longer-than-expected regions of the genome (though for a clade to be significant would require the presence of linked adaptive loci). The method also cannot identify the specific types of genomic variants that might be causing suppression of recombination in heterozygotes, without utilizing other sources of information (such as direct or inverted segmental duplications and other genomic features near the identified regions, or additional analysis of other types of data). However, it provides an alternative line of evidence to methods based on the analysis of paired-end reads, which can miss the presence of complex structural variants or those occurring in regions with poor read mapping.

Applying DoLoReS to the 1KGP ARG reconstructed using Relate identifies a number of regions with both known and novel SVs. Using the ARG allows for the genealogy-based analysis of the age and population frequencies of an SV, and potentially identification of recurrent inversion events. Our tool identifies a large and relatively common inversion on chromosome 10, which has remained unobserved using previous methods due to a lack of clear signal in this region from paired-end read mapping. This demonstrates the power of our method to pick up signals of localized recombination suppression. We limited our detailed examination to regions of size at least 50 kb which are well-supported by SNPs, but note that there is a large number of other smaller regions with significant *P*-values (SI, supplementary fig. S20, Supplementary Material online), which are more difficult to confidently validate. In general, while our theoretical results hold for clades of any size greater than one, in practice we limited our investigation to large and well-supported clades, since ARG reconstruction is noisy and error-prone, and we sought to focus on the strongest signals to investigate further.

It is difficult to provide guarantees on when our method will achieve a certain false positive rate outside of the scenarios we simulated, since this will depend on the ARG reconstruction method and the properties of the data, so will be application-dependent. We recommend performing simulation studies tailored to the specific species and dataset at hand, to check the performance of this and other ARG-based methods, and calibrate the input parameters. We analyzed a large number of relevant metrics and orthogonal evidence to classify the likely reason for recombination suppression within each significant region, including those capturing ARG reconstruction quality (e.g. the proportion of SNPs not uniquely mapping to local trees), whether or not the hits span a centromere, genomic features (e.g. presence of segmental duplications), overlap with genes, an analysis of reads and sequencing depth, analysis of HPRC data using *k*-mer based approaches, and a search of the literature for previous evidence of SVs in these regions. The full details are presented in Supplementary Table S1, Supplementary Material online. As shown in SI, supplementary fig. S21, Supplementary Material online, these measures can help to delineate between likely SVs and other sources of suppression.

Roughly half of the detected regions show no clear signals of structural variation based on our analysis. We suggest that other, non-structural reasons may explain allele-specific suppressed recombination in these regions. Previous evidence suggests that recombination crossovers are suppressed within the boundaries of genes expressed in meiosis (McVicker and Green 2010). This suggests that expression quantitative trait loci (eQTLs) altering meiotic gene expression might impact crossover rates by suppressing recombination crossovers on carriers of one particular allele and in particular in heterozygous individuals, similar to structural drivers. To test whether the identified regions and carriers supported this possibility, we first tested (as detailed in Section 4.5.6) whether our regions are enriched for closely matching gene boundaries. We observe strong enrichment (observed 14 single-gene regions, expected 0.67, OR = 31.2, $P=5\cdot 10^{-10}$), with most of the observed overlaps (10) among those not showing structural evidence. Moreover, the 14 corresponding genes are significantly enriched for being highly expressed during male gametogenesis (9 genes; OR=3.2; P=0.047). Secondly, we tested whether SNPs defining carriers of recombination-suppressed alleles are enriched for known *cis*-eQTLs. Again, we see evidence of enrichment ($P=2\cdot 10^{-3}$), although whether these function in meiotic tisues is unknown. Specific example eQTL regions include *SCMH1* on 1p34.2, *SPATA6* on 1p33, and *ZFAND3* on 6p21.2, all essential for normal spermatogenesis (de Luis et al. 1999; Takada et al. 2007; Yuan et al. 2015). This enrichment of our regions for almost perfect overlap with genes, and eQTLs altering their expression, supports the hypothesis of meiotic allele-dependent suppression of recombination within genes. In contrast, although many (30) of our regions are in LD with GWAS hits, this overlap is not significantly higher than expected by chance (P=0.14). Neither do we observe significant excess overlap with regions under selection identified by Akbari et al. (2024) (P=0.60), although several regions contain one or more significant selection SNPs: 12q24.11 (110.7–111.1 Mb), 9p21.1 (31.9–32.1 Mb), 2p23.1 (31.8–32.4 Mb), 17q21.31 (43.6–44.4 Mb), with only the last possessing clear evidence of structural variation. Overall, the data suggest mainly mechanistic drivers of suppressed inter-allelic recombination crossover regions, rather than, for instance, local epistatic selection among trait-influencing variants.

It is clear that even though Relate only uses SNP data and does not explicitly model the presence of SVs, the reconstructed ARG faithfully captures some of these signals. While this allows for their detection, this also means that the genealogies can be distorted by the presence of SVs, most obviously through the phasing errors that they induce in the data. This highlights the value of considering structural variation as an important source of information when developing future ARG reconstruction and analysis methods.

## Materials and methods

### Probability that an Edge is Disrupted by a Recombination Event

Let T be a fixed local tree, and consider a particular edge *b* within this tree. We would like to calculate, under the SMC’ and conditional on T, the probability $P_{T}(b\;\text{disrupted})$ that the next recombination event arriving along the genome disrupts *b*: that is, it changes the time-length of *b*, or the topology of the tree around *b* (we refer to the latter as *b* being *topologically disrupted*). The possible events that can cause *b* to be disrupted are when (i) the recombination point is on *b* and the coalescence point is not on *b*, or (ii) the recombination point is not on *b* but the coalescence point is (SI, supplementary fig. S1, Supplementary Material online). The full list of event types that do and do not disrupt *b* are illustrated in SI, supplementary fig. S2, Supplementary Material online. By integrating over the possible positions of the recombination point and new coalescence event, we calculate the probability of each illustrated event as detailed in SI, supplementary Section S1.3, Supplementary Material online. The resulting expression is in closed form and can be calculated for any given tree and branch. We derive an equivalent probability $P_{T}(b\;\text{topologically disrupted})$, which only takes into account topology-changing recombination events, as detailed in SI, supplementary Section S1.4, Supplementary Material online.

### Distribution of Edge Span

Conditional on the local tree T, for a particular branch *b*, we are interested in the distribution of the genomic span before *b* is disrupted by a recombination event. We approximate this distribution under the SMC’ by making the assumption that as recombination events arrive along the genome, if they do not disrupt *b* then they also do not otherwise change the rest of the local tree (so T stays fixed along the genome). Then since recombination events arrive along the genome as a Poisson process with rate $L_{T}\cdot \rho /2$ (where $L_{T}$ is the total branch length of T), by thinning, recombination events that disrupt *b* arrive at rate


(2) $$P_{T}(b\;\text{disrupted})\cdot L_{T}\cdot \rho /2.$$

Thus the waiting time until *b* is disrupted by a recombination event is exponential with this rate. We derive an equivalent result when the recombination rate is not constant along the genome (SI, supplementary Section S1.8, Supplementary Material online), when considering only topology-changing events (SI, supplementary Section S1.8.1, Supplementary Material online), and when we condition on the branch having at least one mutation event (SI, supplementary Section S1.8.2, Supplementary Material online).

The assumption that recombination events do not change T within the span of *b* is very strong. However, through quantifying the effect of recombination on the height (SI, supplementary Section S1.7, Supplementary Material online) and total branch length (SI, supplementary Section S1.6, Supplementary Material online) of local trees, we show that the averaged effect of recombination on the rest of the tree does not appear to significantly affect the probability that the given edge is disrupted, making this an excellent approximation (SI, supplementary Section S1.11, Supplementary Material online).

### Distribution of Clade Span

For a given local tree T, we define each clade *G* through the samples it contains as in Section 2.3. We calculate the probability $P_{T}(G\;\text{disrupted})$ that, under the SMC’ and conditional on T, *G* is disrupted by the next recombination event along the genome: that is, that the membership of sample nodes in the clade changes in the next local tree (allowing events that disrupt edges within the clade without changing the group of subtended samples). This can happen when the recombination point is on an edge within the clade and the coalescence point is on an edge outside the clade, or if the recombination point is on an edge outside the clade and the coalescence point is on an edge within the clade, as illustrated in SI, supplementary fig. S3, Supplementary Material online. We calculate the probability of these events as detailed in SI, supplementary Section S1.5, Supplementary Material online.

The distribution of the genomic span of a clade *G* can then be similarly approximated by making the assumption that recombination events that do not disrupt *G* do not change T; then the waiting time until *G* is disrupted by a recombination event is exponentially distributed with rate


$$P_{T}(G\;\text{disrupted})\cdot L_{T}\cdot \rho /2.$$

We derive an equivalent result when the recombination rate changes along the genome (SI, supplementary Section S1.9, Supplementary Material online).

#### Test 1

Given an ARG, for each clade $G^{\left(i\right)}$ we calculate its genomic span [a,b], and use the approximation (2) to compute a corresponding one-sided *P*-value to test whether $G^{\left(i\right)}$ has a significantly longer span than under the null hypothesis of no local (between-clade) recombination suppression:


$$p_{i}=\exp\;\left(-P_{T}(G^{\left(i\right)}\;\text{disrupted})\cdot L_{T}\cdot \int_{a}^{b}\rho (w)/2dw\right),$$

where ρ(w) is the recombination rate at position *w* (SI, supplementary Section S1.9, Supplementary Material online). Simulation studies show that the test has excellent performance for simulated ARGs even for small inversions, and maintains good sensitivity for detecting inversions of over 50 kb for Relate ARGs, while accurately pinpointing their position (SI, supplementary Section S1.13.2, Supplementary Material online).

#### Test 2

Under the model described above, an equivalent test can be constructed using the number of recombination events *R* occurring within the genomic span of *G*, which has a geometric distribution with rate $P_{T}(G\;\text{disrupted})$ (SI, supplementary Section S1.14, Supplementary Material online). For each clade $G^{\left(i\right)}$ within a given ARG, we thus calculate a corresponding (one-sided) *P*-value


$$p_{i}={\left[1-P_{T}(G^{\left(i\right)}\;\text{disrupted})\right]}^{R-1}.$$

In practice, *R* is unknown, so we instead calculate the number of breakpoints between local trees within [a,b] (this is conservative as it strongly underestimates the number of recombination events in this interval).

### 1 KGP ARG

We use tskit (Kelleher et al. 2016) to split the ARGs into the five super-populations (EUR: European, AFR: African, SAS: S. Asian, EAS: E. Asian and AMR: American), and analyze them separately. We adjusted for varying population size (as estimated by Relate) as detailed in SI, supplementary Section S1.9.1, Supplementary Material online. We also applied the corrections detailed in SI, supplementary Section S1.13.1, Supplementary Material online (setting L=1 cM), which correct for reconstruction error and also the presence of gene conversion within inversions (by extending the calculated span of a clade if it disappears and then reappears within a short genomic span). We used the 1KGP genomic mask (which marks whether each nucleotide passes a set of quality filters, based on depth of coverage and reads mapping) to set the recombination rate in regions marked as “not passing” to 0 (to avoid false positives for Test 1). Additionally, to be robust to the presence of phasing switch errors in the data, instead of defining a clade by the samples it contains as described in Section 2.3, we instead count how many samples from each individual it contains. That is, we assign each clade *G* a “genotype” ID $\left(G_{1},\ldots ,G_{N}\right)$, where $G_{i}$ is the number of sequences (0, 1, or 2) within *G* from individual *i*, and consider the clade present in a given local tree if there is a clade with the same genotype ID. We filter out clades supported by fewer than 10 mutations, spanning less than 50 kb or fewer than 10 local trees, and those having fewer than 10 or more than N−10 samples (where *N* is the total number of samples in the ARG); this leaves 107k clades. Tests 1 and 2 are then applied for each remaining clade independently (using the HapMapII GRCh37 recombination map for Test 1, and counting the number of local tree breakpoints to estimate the number of recombination events *R* for Test 2). We require both *P*-values to be below the Bonferroni-corrected threshold of $1\cdot 10^{-12}$ (being 0.05 divided by the total number of clades in the ARGs).

### Analysis of Results

For each significant clade *G*, in each local tree within its genomic span, we identify the clade $\tilde{G}$ with which it is most highly correlated (as measured by the correlation coefficient between their genotype IDs). In Fig. 9a, the span of each significant clade *G* is plotted as a solid horizontal line (in colour corresponding to the super-population), if within the local tree at the given genomic position, there is a clade $\tilde{G}$ with a correlation coefficient of at least 0.95 (and in grey if the correlation coefficient is between 0.9 and 0.95). The leftmost and rightmost positions at which the clade appears in the ARG exactly are indicated by triangles.

We also compute the combined genotype ID for the corresponding predicted clade of samples in the full (all-population) ARG, and calculate its genomic span (shown in black in Fig. 9a). Existence of this “superclade” provides additional evidence that the clades identified independently in each population ARG are not false positives.

#### Genomic Features and Measures of ARG Reconstruction Quality

For each identified region, we extract the positions of nearby genes, using the UCSC Genome Browser NCBI RefSeq track (Pruitt et al. 2005). To check for known genomic features that tend to co-occur with SVs, we also extract the positions of segmental duplications of length greater than 1 kb and >90% sequence similarity falling within this region (Bailey et al. 2001, 2002), and the positions of SVs in the 1KGP call set (Sudmant et al. 2015). To detect issues caused by ARG reconstruction artifacts, we extract the positions of breakpoints between adjacent local trees, and the regions masked during ARG reconstruction (labelled as “not passing” in the 1 KGP pilot mask); we also calculate a moving average (in 10 kb windows) of the proportion of SNPs in the 1KGP data that cannot be uniquely mapped onto a branch in the local tree at the corresponding position (as a measure of ARG reconstruction error). To detect instances where poor estimation of event times may inflate the probability that the clade is disrupted by recombination, we also checked (for each significant clade) the proportion of mutations that fall within vs. outside the clade, and compared this to the average proportion of tree total branch length within vs. outside the clade (expecting these quantities to be similar if times are well estimated).

#### Phasing Errors

Using the individual-based definition of a clade means that it is possible for two clades in the same local tree to have identical IDs, if one clade contains one chromosome from each of *k* individuals, and the other clade contains the other chromosome for each of these *k* individuals. This has negligible probability to arise by random chance unless *k* is very small (4 or less, based on simulations with 1KGP-like parameters). This can, however, arise for larger *k* as the result of phasing errors due to structural variation, in particular due to mis-alignment of CNVs which results in ARG reconstructed artifacts. We thus record all instances where, in a local tree, two clades of size at least six have identical IDs, to look for this signal.

#### Inversion Age

A lower and upper bound on the age of an inversion can be obtained by identifying the most highly correlated clade $\tilde{G}$ in each local tree within the genomic span of *G*, and (if the correlation coefficient is at least 0.9) obtaining the times at the bottom and top end of the branch that subtends $\tilde{G}$ (call these times *s* and *t*, respectively).

**Fig. 10.**
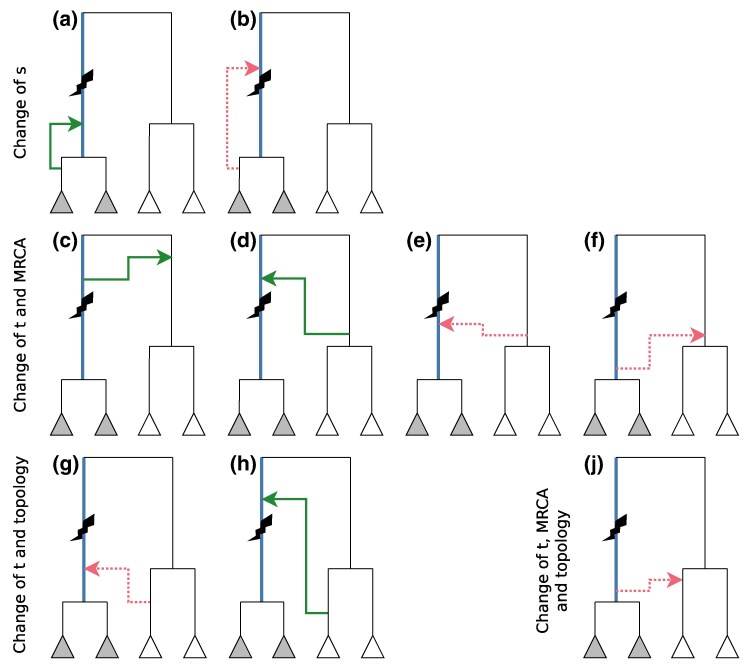
Possible recombination events that change *s* (time of the bottom end of the branch subtending the samples carrying the inversion, shown in blue), *t* (the of the top end of the branch), the MRCA of the carriers and non-carriers, and/or the order of coalescence of the clades. Inversion shown as lightning bolt. Recombination events shown as arrows, where the start of the arrow shows the time and location of the recombination event and the arrowhead shows that of the subsequent coalescence; green solid (resp. red dotted) arrows show feasible (resp. infeasible) events.

While a neutral mutation can arise at any time uniformly distributed along this branch, an inversion prevents certain types of recombination events, so the changes in ARG topology and in *s* and *t* along the genome are informative of its age. Suppose that the inversion is old. Then Figs. 10a,b imply *s* should be relatively recent (being approximately distributed as the coalescence time of *k* samples), while Figs. 10c,d,h imply that both *t* and the MRCA time will be large, since they are constrained to be larger than the age of the inversion. Moreover, if the carriers and non-carriers form two (disjoint) clades in the ARG, the only type of event that can change this topology is that shown in Fig. 10h, which has a very low probability under the SMC’ assumptions. However, a double crossover within the inverted region can allow any of the events shown in red, locally changing *s*, *t*, the MRCA time and/or the topology, in the region between the two recombination breakpoints.

This description aligns with the observed ARG topology and branch time estimates for the 17q21.31 inversion (SI, supplementary fig. S22, Supplementary Material online): the carriers and non-carriers form two disjoint clades with a very large MRCA time for most of the inverted region, apart from the region highlighted in red in Fig. 7. In this region the corresponding local tree topologies change in a way consistent with an event of type F around position 43.86 Mb (which changes *t* and the MRCA time), followed by a number of recombination events of type J between 43.87–43.90 Mb (which change *t*, the MRCA time, and the tree topology as shown in SI, supplementary fig. S22K, Supplementary Material online).

#### Significant eQTLs and GWAS Hits in LD with Identified Variants

For each identified significant clade, we searched the Open Targets Genetics catalog (Ghoussaini et al. 2021; Mountjoy et al. 2021) for genome-wide significant GWAS hits ($P<5\cdot 10^{-8}$) in LD ($r^{2}>0.6$) with SNPs supporting the clade. The full details of the identified SNPs are presented in supplementary table S2, Supplementary Material online. We also checked SNPs supporting the clade for significant associations with gene expression using the QTL catalog.

#### Analysis of HPRC Data

For each identified significant region, we predicted the carrier status of each sequence in the HPRC dataset by checking whether it carries SNPs that occur on the branches that subtend the significant clades. For each sequence, we then calculated *k*-mer counts (setting k=20) in (and around) the region, and counted the number of *k*-mers deleted, duplicated, or inverted when compared to the T2T-CHM13v2 reference. We then calculated the correlation between these *k*-mer counts and predicted carrier status. To look for changes in ordering indicating rearrangements or inversions, we also (i) plotted the ordering of genes in the region as annotated for each sequence, and (ii) selected a subsample of equally spaced *k*-mers on the T2T-CHM13v2 reference, and plotted the relative positions of these *k*-mers on each sequence.

#### Testing for Enrichment

We tested for enrichment of our identified regions for overlapping with genes, genes involved in meiosis, SNPs under selection, GWAS hits and eQTLs. For each significant region we selected a tagging SNP in the middle of the region, constructed a list of 10 best-matched SNPs on the same chromosome (matching on the overall frequency, frequency in Europeans, and average recombination rate within the surrounding 1 Mb region), and defined a region around the matched SNPs of the same physical (and approximately the same genetic) size. We then checked each matched region for overlapping SNPs under selection from Akbari et al. (2024), genes (using the Genome Browser NCBI RefSeq track), genes involved in meiosis (taking clusters 2–4 from Xia et al. (2020, Table S3) and filtering for genes within the top 25% by expression level in testis bulk sequencing data (obtained from the GTEx portal on 19/01/2025, file gene_reads_v10_testis.gct.gz), and GWAS hits and eQTLs as described in Section 4.5.4 (filtering the Open Targets eQTLs for those with an association score of at least 0.6). We then calculated bootstrapped *P*-values by resampling using these matched regions.

### Simulation Parameters

#### Neutral Simulations

Using stdpopsim (Adrion et al. 2020), a library of standardized population genetic simulation models integrated with msprime, we simulated two ARGs under the SMC’ with n=100 (haploid samples) and the following two sets of parameters:

- dataset 1: Chr21 with HapMapII_GRCh38 recombination map, mutation rate $1.29\cdot 10^{-8}$ per site per generation, $N_{e}=10,000$ diploids, constant population size model;
- dataset 2: 5 Mb of Chr21 with flat recombination map, recombination rate $1.2\cdot 10^{-8}$ per site per generation, mutation rate $1.29\cdot 10^{-8}$ per site per generation, $N_{e}=10,000$ diploids, constant population size model.

The mutation rate and $N_{e}$ estimates are the defaults in stdpopsim and in line with other commonly-used estimates for human data (Takahata 1993; Scally and Durbin 2012).

For each simulated dataset, we used ARGweaver, Relate (v1.1.9), tsinfer/tsdate (v0.3.0 and v0.1.5), and ARG-Needle to reconstruct an ARG, using the true simulation parameters as inputs for each tool (and for ARGweaver, a time discretisation grid with 100 points and selecting the MAP ARG from 1,000 posterior samples, for ARG-Needle using 50 time discretisation points). We sense-checked the output of the ARG reconstruction methods using a number of metrics (comparing the MRCA times, local tree topologies, and LD decay, against the simulated ARGs).

#### Simulations with Inversion

We used SLiM (Haller and Messer 2019; Haller et al. 2019) to simulate an ARG with one inversion (n=100, 5 Mb with recombination rate $1\cdot 10^{-8}$ per bp per generation, constant population size 10 000, inverted segment length 200 kb, neutral mutations at rate $1\cdot 10^{-8}$ per bp per generation added using msprime). We used the recipe in Section 14.4 of the SLiM manual (version of 31 August 2024), which simulates balancing selection through a frequency-dependent fitness effect 1−(f−0.5)⋅0.2 (where *f* is the current frequency of the inversion), to maintain the inversion at near intermediate frequency.

#### 1KGP Simulation

To simulate data using parameters similar to the 1KGP data, we used stdpopsim with the AmericanAdmixture_4B11 demographic model, simulating the same number of African, European, Asian and Admixed samples as in the 1KGP, for chromosomes 18–22 (using the HapMapII GRCh37 recombination map). We then applied the 1KGP genomic mask and reconstructed an ARG using Relate, and applied the methods described in Section 4.4 to calculate *P*-values, using a Bonferroni-corrected significance threshold of $1\cdot 10^{-9}$ (using the individual-based definition of a clade). The results are shown in Fig. 6 (right panel).

## Supplementary Material

msaf190_Supplementary_Data

## Data Availability

Code implementing DoLoReS is publicly available at github.com/a-ignatieva/dolores. Scripts used to produce and analyze the simulated and 1KGP data are publicly available at github.com/a-ignatieva/dolores-paper. Simulated data and 1KGP results are publicly available at doi.org/10.6084/m9.figshare.29256770.v1.
