# Supplementary material for: The Length of Haplotype Blocks and Signals of Structural Variation in Reconstructed Genealogies
Source: Mol Biol Evol. 2025 Aug 6;42(9):msaf190. doi: 10.1093/molbev/msaf190 (PMC12400028; doi:10.1093/molbev/msaf190)
Supplement: msaf190_Supplementary_Data [file msaf190_supplementary_data.zip › manuscript_revised_SI.pdf]

Supplementary Information

S1 Supplementary Methods **1**
S1.1 The SMC' model and ARG reconstruction . . . 1
S1.2 Notation and background . . . 2
S1.3 Probability that an edge is disrupted by a recombination event . . . 3
S1.4 Probability that an edge is topologically disrupted by a recombination event . . 6
S1.5 Probability that a clade is disrupted by a recombination event . . . 7
S1.6 Change in total branch length of tree following a recombination event . . . 8
S1.7 Change in tree height following a recombination event . . . 9
S1.8 Distribution of edge span . . . 9
S1.9 Distribution of clade span . . . 12
S1.10 Quality of approximation to the distribution of edge span . . . 13
S1.11 Effects of recombination on a local tree . . . 13
S1.12 Comparison of simulation models . . . 15
S1.13 Detection of local recombination suppression: Test 1 . . . 16
S1.14 Detection of local recombination suppression: Test 2 . . . 19

S2 Proofs **22**

S3 Supplementary Figures **34**

S1 Supplementary Methods

S1.1 The SMC' model and ARG reconstruction

ARGs were first described by Griffiths and Marjoram (1997) as realisations of the coalescent with recombination (CwR), a stochastic process operating backwards in time, generating a genealogy through a sequence of coalescence and recombination events (Hudson, 1983). Wiuf and Hein (1999) reframed the CwR as a stochastic process operating spatially along the genome: starting with the leftmost endpoint, local trees are generated sequentially moving to the right, reshaped by recombination events. While calculating the properties of the ARG under both frameworks is generally intractable, this seminal work spurred on a suite of simplifying approximations, enabling applications to large-scale genomic data.

The sequentially Markovian coalescent (SMC) model, proposed by McVean and Cardin (2005), imposed the assumption that the process along the genome is Markovian, which, in essence, prohibits recombination events in genetic material not ancestral to the sample. This was followed by the SMC' extension (Marjoram and Wall, 2006), which was shown to be an excellent approximation to the CwR, based on the joint distribution of pairwise coalescent times and a quantification of bias in population size estimates (Wilton et al., 2015), and the distribution of the next local tree conditional on the current one in a two-locus model (Hobolth and Jensen, 2014). Thus, for a small trade-off in accuracy, the SMC' model offered a substantially more tractable way of calculating analytic approximations to various quantities of interest, such as the correlation between coalescence time and linkage probability for a randomly sampled pair of sequences (Eriksson et al., 2009), and identity-by-descent tract length distributions and related quantities (Harris and Nielsen, 2013; Carmi et al., 2014). It also enabled the development of powerful new inference methods: for instance, by considering the genealogy of a single pair of sequences, Li and Durbin (2011) developed a HMM-based approach (the pairwise SMC, or PSMC) for inferring the history of human population sizes, which was subsequently extended by Schiffels and Durbin (2014) to multiple samples.

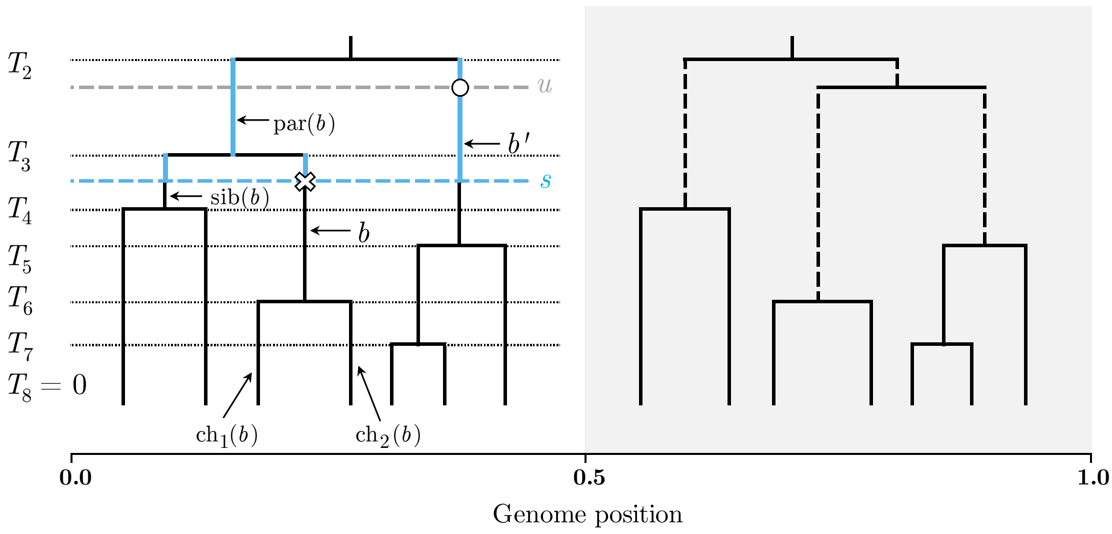

Figure S1: Illustration of the notation used throughout. The ARG has two marginal trees, where the tree on the left is $\mathcal{T}$, with $n = 7$. Coalescent event times are shown as black dotted lines. For the edge labelled b, $t^{\uparrow}(b) = T_3$, $t^{\downarrow}(b) = T_6$, $d^{\leftarrow}(b) = 0$, $d^{\rightarrow}(b) = 0.5$ (so the span of the edge is 0.5); $\mathcal{A}(b) = \{b, \text{sib}(b), \text{par}(b)\}$ and $\mathcal{B}(b) = \{b, \text{sib}(b), \text{ch}_1(b), \text{ch}_2(b)\}$. The recombination event occurs at genomic position 0.5; the recombination point $\mathcal{R} = (b, s)$ is shown as a cross; the coalescence point $\mathcal{C} = (b', u)$ is shown as a circle; $n(s) = 3$ and $L_{\mathcal{T}}(s)$ gives the total length of the edges shown in blue. The tree on the right $\mathcal{T}'$ is obtained by pruning the subtree below $\mathcal{R}$ and reattaching at $\mathcal{C}$; solid vertical lines show edges that have not been affected by the recombination event.

Meanwhile, the definition of the ARG has become decoupled from its initial description as the realisation of a stochastic process, to more broadly denote a genealogical network that captures genetic inheritance. Using this looser definition, the problem of explicitly reconstructing plausible ARGs from sequencing data has seen significant recent progress driven by the use of heuristic methods and principled approximations to the CwR. ARGweaver (Rasmussen et al., 2014) implements an MCMC scheme based on a time-discretised version of the SMC (or SMC') to obtain posterior samples of ARGs compatible with a given dataset. Relate (Speidel et al., 2019) and tsinfer/tsdate (Kelleher et al., 2019; Wohns et al., 2022) reconstruct a single ARG from data, by using methods based on the Li and Stephens (2003) framework to first reconstruct the topologies and then estimating the edge lengths using Bayesian approaches with coalescent-based priors. ARG-Needle (Zhang et al., 2023) reconstructs a single ARG by sequentially threading in each sample, by first identifying the most closely related samples already in the ARG via genotype hashing, and subsequently estimating coalescence times under the Ascertained Sequentially Markovian Coalescent (ASMC) model (a coalescent-based HMM). These methods scale to thousands of human genome-length samples and have already been applied to many large-scale datasets, resulting in powerful inference of evolutionary events and parameters, such as the history of human demography (Wohns et al., 2022), past population sizes (Speidel et al., 2019), signals of selection (Hejase et al., 2022), and genetic associations for complex traits (Zhang et al., 2023).

S1.2 Notation and background

The notation is illustrated in Figure S1. Let $\mathcal{T}$ be a fixed local tree with n leaves. Denote by T_i (for $i \in \{2, \cdots, n\}$) the population-scaled time at which the number of lineages in $\mathcal{T}$ jumps from i to $i-1$, with T_2 being the time of MRCA and setting $T_{n+1} := 0$. Let $n(t)$ be the number of lineages at time t, so $n(0) = n$, with $n(T_j) = j - 1$ and $n(t) = 1$ for $t \geq T_2$.

For an edge $b \in \mathcal{T}$, denote the lower end time by $t^{\downarrow}(b) \geq 0$ and the upper end time by $t^{\uparrow}(b) \leq T_2$, with the *time-length* of the edge given by $\bar{t}(b) = t^{\uparrow}(b) - t^{\downarrow}(b)$. Let $d^{\leftarrow}(b)$ and $d^{\rightarrow}(b)$ be the leftmost and rightmost endpoints of the genomic span of edge b, respectively, with its *span* given by $d^{\rightarrow}(b) - d^{\leftarrow}(b)$.

Let $\mathrm{par}(b), \mathrm{sib}(b), \mathrm{ch}_1(b)$ and $\mathrm{ch}_2(b)$ denote the parent, sibling, left child and right child edge of b respectively, such that we have the following relations:

$$\begin{aligned}
&t^{\downarrow}(\mathrm{par}(b)) = t^{\uparrow}(b)\\
&t^{\uparrow}(\mathrm{sib}(b)) = t^{\uparrow}(b),\\
&t^{\uparrow}(\mathrm{ch}_1(b)) = t^{\uparrow}(\mathrm{ch}_2(b)) = t^{\downarrow}(b),\\
&\mathrm{ch}_1(b) = \mathrm{ch}_2(b) = \emptyset \text{ if } t^{\downarrow}(b) = 0 \text{ (b extends from a leaf node).}
\end{aligned}$$

Define the sets of edges $\mathcal{A}(b) := \{b, \mathrm{sib}(b), \mathrm{par}(b)\}$ and $\mathcal{B}(b) := \{b, \mathrm{sib}(b), \mathrm{ch}_1(b), \mathrm{ch}_2(b)\}$, and denote by b_r the root lineage extending past the MRCA node.

Let $L_{\mathcal{T}}(t)$ be the sum of edge lengths in $\mathcal{T}$ above time t and up to T_2:

$$L_{\mathcal{T}}(t) = \sum_{b \in \mathcal{T}: t^{\uparrow}(b) > t} \left[t^{\uparrow}(b) - \max(t, t^{\downarrow}(b)) \right],$$

so that $L_{\mathcal{T}}(0)$ is the total branch length of $\mathcal{T}$ (condensed as $\mathcal{L}_{\mathcal{T}} := L_{\mathcal{T}}(0)$ in the main text). Denote by $\mathcal{T}_x$ the local tree at position x along the genome.

Under the SMC', moving along the genome, a recombination event happens after an exponentially distributed waiting time with rate $\mathcal{L}_{\mathcal{T}}(0) \cdot \rho/2$; when this event happens, a location $\mathcal{R}$ is selected uniformly at random along the edges of $\mathcal{T}$, say on edge b at time s, which we denote as $\mathcal{R} \in b$ or $\mathcal{R} = (b, s)$. A new coalescence point $\mathcal{C}$ is selected by allowing the recombining lineage to coalesce at rate 1 with all the lineages present above time s (including b_r). We denote a coalescence point on edge b' at time u as $\mathcal{C} \in b'$ or $\mathcal{C} = (b', u)$. The next tree along the genome is then formed by pruning the subtree below the recombination point $\mathcal{R}$ and reattaching it at the chosen coalescence point $\mathcal{C}$. We write $\mathcal{R} \in \mathcal{B}(b)$ to mean that the recombination point is on one of the edges in $\mathcal{B}(b)$.

The difference with the spatial formulation of the CwR is that the coalescence point is restricted to be on the local tree $\mathcal{T}$, whereas under the CwR it could be placed on any edges of the ARG corresponding to the full sequence of trees to the left of the recombination position. In essence, the SMC' approximation disallows any recombination events that occur in non-ancestral material, making the process Markovian along the genome.

The difference between the SMC and SMC' models is that under the SMC', the coalescence point can be chosen on the same edge b containing the recombination point, above time s (so that recombinations can occur that do not change the tree topology or edge lengths), whereas events of this type are disallowed under the SMC.

S1.3 Probability that an edge is disrupted by a recombination event

Considering a fixed edge $b \in \mathcal{T}$, when a recombination event occurs, we would like to know the probability that b is affected by this recombination event. This includes both changes in the time-length of b and events that change the topology of the clade around b (we will say in these cases that b is *topologically* disrupted by the recombination). This can happen because either (1) the recombination point is on $b' \in \mathcal{B}(b)$ and the coalescence point is not on b', or (2) the recombination point is not on $\mathcal{B}(b)$ and the coalescence point is on b. The possible scenarios that do and do not disrupt b are illustrated in Figure S2.

We have, for a given tree $\mathcal{T}$,

$$\begin{aligned}
1 &= \mathbb{P}_{\mathcal{T}}(\mathcal{R} \in \mathcal{B}(b)) + \mathbb{P}_{\mathcal{T}}(\mathcal{R} \notin \mathcal{B}(b))\\
&= \sum_{b' \in \mathcal{B}(b)} \left[\mathbb{P}_{\mathcal{T}}(\mathcal{C} \notin b' | \mathcal{R} \in b') + \mathbb{P}_{\mathcal{T}}(\mathcal{C} \in b' | \mathcal{R} \in b') \right] \cdot \mathbb{P}_{\mathcal{T}}(\mathcal{R} \in b')\\
&\quad + \left[\mathbb{P}_{\mathcal{T}}(\mathcal{C} \in b | \mathcal{R} \notin \mathcal{B}(b)) + \mathbb{P}_{\mathcal{T}}(\mathcal{C} \notin b | \mathcal{R} \notin \mathcal{B}(b)) \right] \cdot \mathbb{P}_{\mathcal{T}}(\mathcal{R} \notin \mathcal{B}(b)).
\end{aligned}$$

The probability that b is disrupted by the recombination event is thus

$$\mathbb{P}_{\mathcal{T}}(b \text{ disrupted}) = \sum_{b' \in \mathcal{B}(b)} \mathbb{P}_{\mathcal{T}}(\mathcal{R} \in b' \text{ and } \mathcal{C} \notin b') + \mathbb{P}_{\mathcal{T}}(\mathcal{R} \notin \mathcal{B}(b) \text{ and } \mathcal{C} \in b)$$

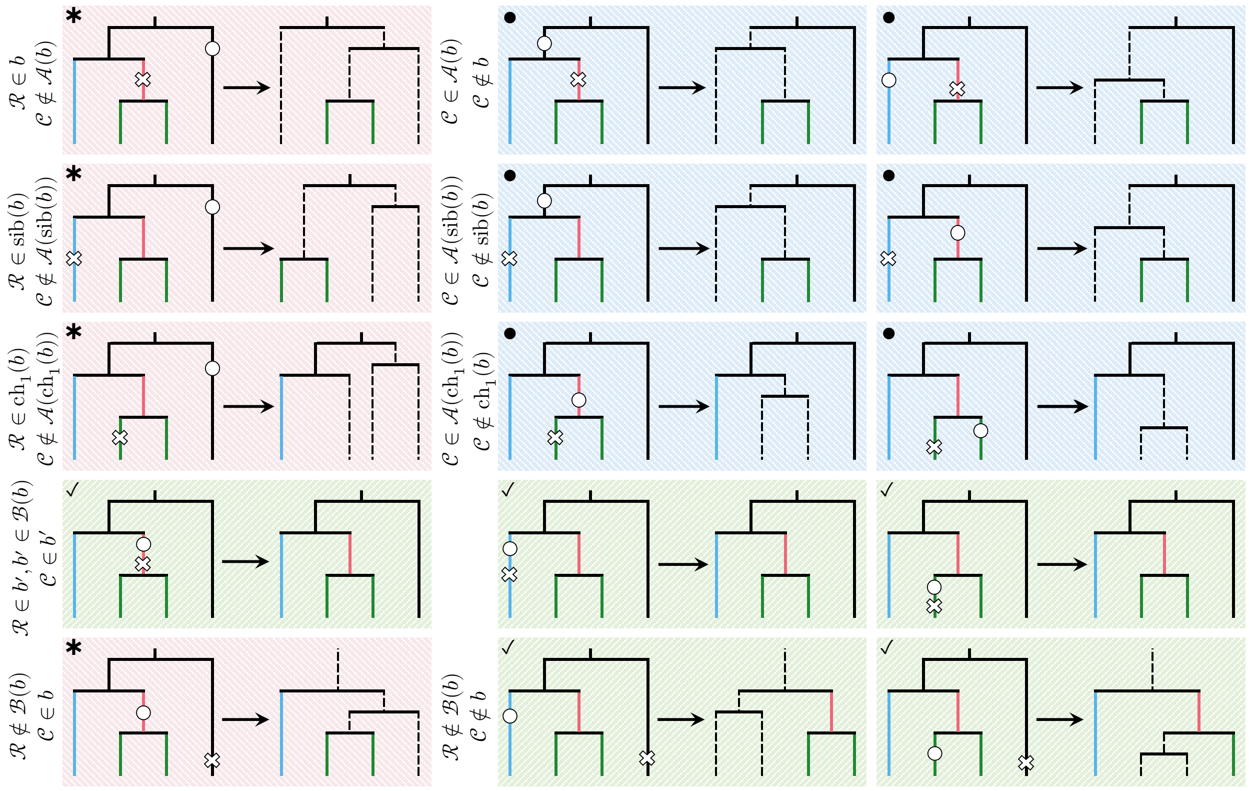

Figure S2: Possible events that do and do not disrupt edge b (shown in red); $\mathrm{sib}(b)$ is shown in blue, $\mathrm{ch}_1(b)$ and $\mathrm{ch}_2(b)$ in green. Recombination points are shown as crosses; coalescence points as circles. edges that are disrupted (or newly added) are shown as dashed lines. Events highlighted in green (marked with ticks) do not disrupt b. Events highlighted in red (marked with stars) disrupt the edge in terms of both edge length and topology (b is topologically disrupted); those highlighted in blue (marked with dots) disrupt b via changing only its time-length.

$$= \sum_{b' \in \mathcal{B}(b)} \mathbb{P}_{\mathcal{T}}(\mathcal{C} \notin b' | \mathcal{R} \in b') \cdot \mathbb{P}_{\mathcal{T}}(\mathcal{R} \in b')$$
$$+ \mathbb{P}_{\mathcal{T}}(\mathcal{C} \in b | \mathcal{R} \notin \mathcal{B}(b)) \cdot \mathbb{P}_{\mathcal{T}}(\mathcal{R} \notin \mathcal{B}(b)). \tag{S1}$$

We now calculate each of these probabilities in turn for an arbitrary edge $\beta \in \mathcal{T}$ under the SMC'.

S1.3.1 Probability recombination point is on edge β

Under the SMC', the recombination point is chosen uniformly at random along the edges of the tree. Thus, the probability that the recombination event happens on edge β is the ratio of the edge length to the total branch length of $\mathcal{T}$, so

$$\mathbb{P}_{\mathcal{T}}(\mathcal{R} \in \beta) = \frac{\bar{t}(\beta)}{L_{\mathcal{T}}(0)}, \tag{S2}$$

and

$$\mathbb{P}_{\mathcal{T}}(\mathcal{R} \notin \mathcal{B}(\beta)) = 1 - \sum_{b' \in \mathcal{B}(\beta)} \frac{\bar{t}(b')}{L_{\mathcal{T}}(0)}. \tag{S3}$$

S1.3.2 Probability coalescence point is not on β given recombination point is on β

The probability that, conditional on the recombination event happening on edge β at time s, the coalescence point is on β has been derived by Deng et al. (2021), which in our notation is as follows.

Proposition S1.1 (Deng et al. (2021), Proposition 1)**.** *Letting $k = n(s)$, so that T_k is the first coalescence time just above time s,*

$$\mathbb{P}_{\mathcal{T}}(\mathcal{C} \in \beta | \mathcal{R} = (\beta, s)) = \frac{1}{k} + e^{ks} \cdot \sum_{j=n(t^{\uparrow}(\beta))+1}^{k} Q_{kj}, \tag{S4}$$

where

$$Q_{kk} := -\frac{1}{k} e^{-kT_k}, \tag{S5}$$

and

$$Q_{kj} = e^{-kT_k} e^{-L_{\mathcal{T}}(T_k)} \frac{1}{j} \left(e^{L_{\mathcal{T}}(T_{j+1})} - e^{L_{\mathcal{T}}(T_j)} \right). \tag{S6}$$

Marginalising out the recombination time s, hence summing over $k = n(s)$ in (S4), gives the following.

Proposition S1.2 (Deng et al. (2021), Proposition 2)**.**

$$\mathbb{P}_{\mathcal{T}}(\mathcal{C} \notin \beta | \mathcal{R} \in \beta) = 1 - \frac{1}{\bar{t}(\beta)} \sum_{k=n(t^{\uparrow}(\beta))+1}^{n(t^{\downarrow}(\beta))} \left(\widetilde{Q}^1(k) + \widetilde{Q}^2(k, k, n(t^{\uparrow}(\beta)) + 1, 0, 1) \right), \tag{S7}$$

where

$$\widetilde{Q}^1(k) := \frac{1}{k} \left(T_k - T_{k+1} \right), \tag{S8}$$

and for $x, y, A, B \in \mathbb{Z}$, $x \geq k$, $2 \leq y \leq x$,

$$\widetilde{Q}^2(k, x, y, A, B) := \frac{1}{k} \left(e^{kT_k} - e^{kT_{k+1}} \right) \sum_{j=y}^{x} (Aj + B) \cdot Q_{kj}. \tag{S9}$$

The proofs, translated into our notation, are given in Sections S2.1 and S2.2.

S1.3.3 Probability coalescence point is on β given recombination point is not on $\mathcal{B}(\beta)$

We start by conditioning on the recombination time s to obtain the following.

Proposition S1.3. *Conditional on the recombination point $\mathcal{R}$ being at time s and on an edge outside the set $\mathcal{B}(\beta)$, with $k = n(s)$, the probability that β is disrupted is*

$$\mathbb{P}_{\mathcal{T}}(\mathcal{C} \in \beta | \mathcal{R} \notin \mathcal{B}(\beta), \mathcal{R} = (\cdot, s)) = \begin{cases} e^{ks} \sum_{j=n(t^{\uparrow}(\beta))+1}^{n(t^{\downarrow}(\beta))} Q_{kj} & s < t^{\downarrow}(\beta) \\ \frac{1}{k} + e^{ks} \sum_{j=n(t^{\uparrow}(\beta))+1}^{k} Q_{kj} & t^{\downarrow}(\beta) \leq s < t^{\uparrow}(\beta) \\ 0 & \text{otherwise.} \end{cases} \tag{S10}$$

with Q_{kk} and Q_{kj} as defined in (S5) *and* (S6)*, respectively.*

The proof is given in Section S2.3.

Let t_1, t_2, t_3, t_4 denote the event times $t^{\downarrow}(\mathrm{ch}_1(\beta)), t^{\downarrow}(\mathrm{ch}_2(\beta)), t^{\downarrow}(\mathrm{sib}(\beta)), t^{\downarrow}(\beta)$ sorted in increasing order, and define $t_0 := 0$ and $t_5 := t^{\uparrow}(\beta)$. Integrating out the recombination time in (S10), we have the following.

Proposition S1.4. *Conditional on the recombination point being on an edge outside the set $\mathcal{B}(\beta)$, the probability that β is disrupted is*

$$\mathbb{P}_{\mathcal{T}}(\mathcal{C} \in \beta | \mathcal{R} \notin \mathcal{B}(\beta)) = \frac{1}{L_{\mathcal{T}}(0) - \sum_{b' \in \mathcal{B}(\beta)} \bar{t}(b')} \left\{ \sum_{k=n(t_1)+1}^{n} k \widetilde{Q}^2(k, n(t^{\downarrow}(\beta)), n(t^{\uparrow}(\beta)) + 1, 0, 1) \right.$$

$$
\begin{aligned}
&+ \sum_{k=n(t_2)+1}^{n(t_1)} (k-1)\widetilde{Q}^2(k, n(t^{\downarrow}(\beta)), n(t^{\uparrow}(\beta)) + 1, 0, 1) \\
&+ \sum_{k=n(t_3)+1}^{n(t_2)} (k-2)\widetilde{Q}^2(k, n(t^{\downarrow}(\beta)), n(t^{\uparrow}(\beta)) + 1, 0, 1) \\
&+ \sum_{k=n(t_4)+1}^{n(t_3)} \Big[\mathbb{1}(t^{\downarrow}(\text{sib}(\beta)) < t^{\downarrow}(\beta))(k-3)\widetilde{Q}^2(k, n(t^{\downarrow}(\beta)), n(t^{\uparrow}(\beta)) + 1, 0, 1) \\
&\qquad + \mathbb{1}(t^{\downarrow}(\text{sib}(\beta)) \geq t^{\downarrow}(\beta))(k-1)\left(\widetilde{Q}^1(k) + \widetilde{Q}^2(k, k, n(t^{\uparrow}(\beta)) + 1, 0, 1)\right)\Big] \\
&+ \sum_{k=n(t_5)+1}^{n(t_4)} (k-2)\left(\widetilde{Q}^1(k) + \widetilde{Q}^2(k, k, n(t^{\uparrow}(\beta)) + 1, 0, 1)\right) \Bigg\}, \qquad \text{(S11)}
\end{aligned}
$$

with $\widetilde{Q}^1$ *and* $\widetilde{Q}^2$ *as defined in* (S8) *and* (S9)*, respectively.*

The proof is given in Section S2.4.

Substituting the expressions (S2), (S3), (S7) and (S11) into (S1) gives the desired probability that edge b is disrupted by the next recombination event.

S1.4 Probability that an edge is topologically disrupted by a recombination event

Most ARG reconstruction algorithms focus on identifying the presence of recombination events through finding patterns of mutations not consistent with tree-like evolution. This, in general, does not allow for the detection of recombination events that only change edge lengths (panels highlighted in blue in Figure S2). We therefore also calculate the probability that an edge b is topologically disrupted (corresponding to panels highlighted in red in Figure S2).

Theorem S1.1. *The probability that an edge b is topologically disrupted by a recombination event is given by*

$$
\begin{aligned}
\mathbb{P}_{\mathcal{T}}(b \text{ topologically disrupted}) = &\sum_{b' \in \mathcal{B}(b)} \mathbb{P}_{\mathcal{T}}(\mathcal{C} \notin \mathcal{A}(b') | \mathcal{R} \in b') \cdot \mathbb{P}_{\mathcal{T}}(\mathcal{R} \in b') \\
&+ \mathbb{P}_{\mathcal{T}}(\mathcal{C} \in b | \mathcal{R} \notin \mathcal{B}(b)) \cdot \mathbb{P}_{\mathcal{T}}(\mathcal{R} \notin \mathcal{B}(b)), \qquad \text{(S12)}
\end{aligned}
$$

with

$$
\mathbb{P}_{\mathcal{T}}(\mathcal{C} \notin \mathcal{A}(\beta) | \mathcal{R} \in \beta) = 1 - \frac{1}{t(\beta)} \sum_{k=n(t^{\uparrow}(\beta))+1}^{n(t^{\downarrow}(\beta))} G_\beta(k),
$$

where, for $k \leq n(t^{\downarrow}(\text{sib}(\beta)))$,

$$
G_\beta(k) := 2 \cdot \widetilde{Q}^1(k) + 2 \cdot \widetilde{Q}^2(k, k, n(t^{\uparrow}(\beta)) + 1, 0, 1) + \widetilde{Q}^2(k, n(t^{\uparrow}(\beta)), n(t^{\uparrow}(\text{par}(\beta))) + 1, 0, 1),
$$

and for $k > n(t^{\downarrow}(\text{sib}(\beta)))$

$$
\begin{aligned}
G_\beta(k) := \widetilde{Q}^1(k) + \widetilde{Q}^2(k, k, n(t^{\downarrow}(\text{sib}(\beta))) + 1, 0, 1) + 2 \cdot \widetilde{Q}^2(k, n(t^{\downarrow}(\text{sib}(\beta))), n(t^{\uparrow}(\beta)) + 1, 0, 1) \\
+ \widetilde{Q}^2(k, n(t^{\uparrow}(\beta)), n(t^{\uparrow}(\text{par}(\beta))) + 1, 0, 1),
\end{aligned}
$$

and $\widetilde{Q}^1$ *and* $\widetilde{Q}^2$ *are as defined in* (S8) *and* (S9)*, respectively.*

The proof is given in Section S2.5.

S1.5 Probability that a clade is disrupted by a recombination event

We now calculate the probability that a particular *clade* of edges is disrupted by the next recombination event, i.e. that the membership of sample nodes in the clade changes from one local tree to the next (but allowing for events that disrupt edges within the clade without changing the group of subtended samples). This can happen when a lineage within the clade recombines and coalesces outside the clade or its root edge, or if a lineage from outside the clade recombines and coalesces into the clade, as illustrated in Figure S3.

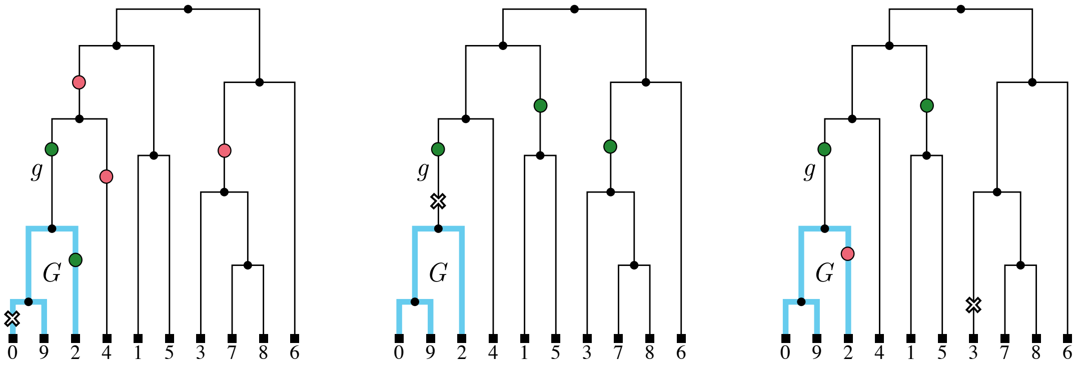

Figure S3: Recombination events that do and do not disrupt a clade. Clade G, subtended by edge g, contains samples $\{0, 2, 9\}$; edges belonging to G are shown in blue. In each tree, for the given recombination point (marked by a cross), red (resp. green) circles show examples of coalescence points that would (resp. would not) result in G being disrupted.

Let G be the set of edges subtended by an edge g, with clade MRCA time $t^{\downarrow}(g) = T_m$, $n_G(t)$ the number of lineages in clade G at time t, abusing notation to write $G \cup g := G \cup \{g\}$. Let $n_{G\cup g}(t)$ be the number of lineages in $G \cup g$ at time t and $L_G(t)$ the total branch length within the clade above time t. Then

$$\begin{aligned}1 = \;&\mathbb{P}(\mathcal{R} \in G) \cdot (\mathbb{P}(\mathcal{C} \notin G \cup g | \mathcal{R} \in G) + \mathbb{P}(\mathcal{C} \in G \cup g | \mathcal{R} \in G)) + \\ &+ \mathbb{P}(\mathcal{R} \in g) \\ &+ \mathbb{P}(\mathcal{R} \notin G \cup g) \cdot (\mathbb{P}(C \notin G | \mathcal{R} \notin G \cup g) + \mathbb{P}(C \in G | \mathcal{R} \notin G \cup g)) \,.\end{aligned}$$

Considering only the events that disrupt the clade, we obtain

$$\mathbb{P}(G \text{ disrupted}) = \mathbb{P}(\mathcal{R} \in G) \cdot \mathbb{P}(\mathcal{C} \notin G \cup g | \mathcal{R} \in G) + \mathbb{P}(\mathcal{R} \notin G \cup g) \cdot \mathbb{P}(C \in G | \mathcal{R} \notin G \cup g). \quad \text{(S13)}$$

Theorem S1.2. *The probability that a clade G is disrupted by a recombination event is*

$$\begin{aligned}\mathbb{P}_{\mathcal{T}}(G \textit{ disrupted}) = \;&\frac{L_G(0)}{L_{\mathcal{T}}(0)} - \frac{1}{L_{\mathcal{T}}(0)} \sum_{k=n(t^{\downarrow}(g)+1)}^{n} \left[n_{G\cup g}(T_{k+1}) \widetilde{Q}^1(k) + \widetilde{Q}^4(k, G \cup g) \right] n_G(T_{k+1}) \\ &+ \frac{1}{L_{\mathcal{T}}(0)} \sum_{k=n(t^{\downarrow}(g))+1}^{n} (k - n_{G\cup g}(T_{k+1})) \left[n_G(T_{k+1}) \widetilde{Q}^1(k) + \widetilde{Q}^4(k, G) \right], \quad \text{(S14)}\end{aligned}$$

where

$$\widetilde{Q}^4(k, A) = \frac{1}{k} \left(e^{kT_k} - e^{kT_{k+1}} \right) \sum_{j=n(t^{\uparrow}(A))+1}^{k} n_A(T_{j+1}) Q_{kj},$$

taking $t^{\uparrow}(G \cup g) = t^{\uparrow}(g)$ and $t^{\uparrow}(G) = t^{\downarrow}(g)$.

The proof is given in Section S2.10.

S1.6 Change in total branch length of tree following a recombination event

Conditioning on the tree $\mathcal{T}$, we now consider the distribution of

$$C = L_{\mathcal{T}'}(0) - L_{\mathcal{T}}(0),$$

the change in total branch length following a single recombination event. First, considering the sign of the change, we have the following.

Proposition S1.5. *The probability of C being negative, zero, or positive is given by*

$$\mathbb{P}_{\mathcal{T}}(C < 0) = \frac{1}{L_{\mathcal{T}}(0)} \sum_{b \in \mathcal{T}} \sum_{k=n(t^{\uparrow}(b))+1}^{n(t^{\downarrow}(b))} \left((k-1)\widetilde{Q}^1(k) + \widetilde{Q}^2(k, k, n(t^{\uparrow}(b)+1, 1, -1) \right) \tag{S15}$$

$$\mathbb{P}_{\mathcal{T}}(C = 0) = \frac{1}{L_{\mathcal{T}}(0)} \sum_{b \in \mathcal{T}} \sum_{k=n(t^{\uparrow}(b))+1}^{n(t^{\downarrow}(b))} \left(\widetilde{Q}^1(k) + \widetilde{Q}^2(k, k, n(t^{\uparrow}(b)) + 1, 0, 1) \right) \tag{S16}$$

$$\mathbb{P}_{\mathcal{T}}(C > 0) = \frac{1}{L_{\mathcal{T}}(0)} \sum_{b \in \mathcal{T}} \sum_{k=n(t^{\uparrow}(b))+1}^{n(t^{\downarrow}(b))} \left(\widetilde{Q}^2(k, n(t^{\uparrow}(b)), 2, 1, 0) + \widetilde{Q}^3(k) \right), \tag{S17}$$

respectively, where

$$\widetilde{Q}^3(k) = \frac{1}{k} \left(e^{-L_{\mathcal{T}}(T_k)} - e^{-L_{\mathcal{T}}(T_{k+1})} \right). \tag{S18}$$

The proof is given in Section S2.6.

To explore the distribution of the magnitude of the change in edge length (when this is non-zero), we derive an approximation of its density.

Proposition S1.6. *Conditional on the change in total branch length being non-zero, the density of C is given approximately by*

$$p^C_{\mathcal{T}}(c | C \neq 0) = \frac{1}{L_{\mathcal{T}}(0)} \sum_{b \in \mathcal{T}} \xi_b(c), \tag{S19}$$

where $\xi_b(c)$ is given by

$$\begin{cases} (n(c+t^{\uparrow}(b))-1) \left(e^{-(l-1)(c+t^{\uparrow}(b))} \cdot \sum\limits_{k=l}^{n(t^{\downarrow}(b))} \widetilde{P}^1_{kl} - \frac{e^{(l-1)(T_l - t^{\uparrow}(b) - c)} - 1}{l-1} \right) & t^{\uparrow}(b) \neq T_2,\ -\bar{t}(b) \leq c < 0 \\ (n(c+2T_2-T_3)-1) \left(e^{-(l-1)(c+2T_2-T_3)} \cdot \sum_{k=l}^{n(t^{\downarrow}(b))} \widetilde{P}^1_{kl} - \frac{e^{(l-1)(T_l - c - 2T_2 + T_3)} - 1}{l-1} \right) & t^{\uparrow}(b) = T_2, \\ & t^{\downarrow}(b) + T_3 - 2T_2 \leq c < 2(T_3 - T_2) \\ \frac{1}{2}(n(c/2+T_2)-1) \left(e^{-(c/2+T_2)} \cdot \sum_{k=l}^{n(t^{\downarrow}(b))} \widetilde{P}^1_{kl} - e^{T_l - c/2 - T_2} + 1 \right) & t^{\uparrow}(b) = T_2,\ 2(T_3 - T_2) \leq c < 0 \\ n(c+t^{\uparrow}(b)) e^{-\left[L_{\mathcal{T}}(t^{\uparrow}(b)) - L_{\mathcal{T}}(c+t^{\uparrow}(b))\right]} \cdot \sum\limits_{k=n(t^{\uparrow}(b))+1}^{n(t^{\downarrow}(b))} \widetilde{P}^2_k & 0 < c \leq T_2 - t^{\uparrow}(b) \\ \frac{1}{2} e^{-(c+t^{\uparrow}(b)-T_2)/2} \cdot \sum\limits_{k=n(t^{\uparrow}(b))+1}^{n(t^{\downarrow}(b))} \widetilde{P}^3_k & c > T_2 - t^{\uparrow}(b) \\ 0 & \textit{otherwise.} \end{cases}$$

and

$$\widetilde{P}^1_{kl} := \frac{1}{k-1} \left(e^{(k-1)T_k} - e^{(k-1)T_{k+1}} \right) \exp\left(l \cdot T_l - k \cdot T_k + L_{\mathcal{T}}(T_l) - L_{\mathcal{T}}(T_k) \right)$$

$$\widetilde{P}^2_k := \frac{1}{k-1} \left(e^{(k-1)T_k} - e^{(k-1)T_{k+1}} \right) \exp\left(t^{\uparrow}(b) - k \cdot T_k - L_{\mathcal{T}}(T_k) + L_{\mathcal{T}}(t^{\uparrow}(b)) \right)$$

$$\widetilde{P}^3_k := \frac{1}{k-1} \left(e^{(k-1)T_k} - e^{(k-1)T_{k+1}} \right) \exp\left(t^{\uparrow}(b) - k \cdot T_k - L_{\mathcal{T}}(T_k) \right).$$

The proof is given in Section S2.7. This is an approximation rather than an exact result under the SMC', since it assumes that after conditioning on the coalescence point not being on the same branch as the recombination point, the coalescent dynamics follow the SMC model (we find this to give a very close approximation, and simplifies our calculations).

S1.7 Change in tree height following a recombination event

The height of the tree, $H(\mathcal{T}) = T_2$, can change following a recombination event if (1) the coalescence point is above T_2, in which case a new root is formed and the tree height increases, or (2) the recombination happens on one of the two lineages descending from the MRCA, then the tree height can either increase or decrease. Let the set $\mathcal{M} := \{\text{ch}_1(b_r), \text{ch}_2(b_r)\}$ contain the two edges descending from the MRCA, and let $H = H(\mathcal{T}') - H(\mathcal{T})$ be the magnitude of the change in height. Then we have the following.

Proposition S1.7. *Conditional on $\mathcal{T}$, the probability of the change in tree height being negative, zero, or positive is given by*

$$\mathbb{P}_{\mathcal{T}}(H < 0) = \frac{1}{L_{\mathcal{T}}(0)} \sum_{b \in \mathcal{M}} \sum_{k=2}^{n(t^{\downarrow}(b))} \left\{ (k-1)\widetilde{Q}^1(k) - \widetilde{Q}^2(k,k,2,0,1) - \widetilde{Q}^3(k) \right\} \tag{S20}$$

$$\mathbb{P}_{\mathcal{T}}(H = 0) = \frac{1}{L_{\mathcal{T}}(0)} \left\{ \sum_{b \in \mathcal{M}} \sum_{k=2}^{n(t^{\downarrow}(b))} \left(\widetilde{Q}^1(k) + \widetilde{Q}^2(k,k,2,0,1) \right) + \sum_{b \notin \mathcal{M}} \left(\bar{t}(b) - \sum_{k=n(t^{\uparrow}(b))+1}^{n(t^{\downarrow}(b))} \widetilde{Q}^3(k) \right) \right\} \tag{S21}$$

$$\mathbb{P}_{\mathcal{T}}(H > 0) = \frac{1}{L_{\mathcal{T}}(0)} \sum_{b \in \mathcal{T}} \sum_{k=n(t^{\uparrow}(b))+1}^{n(t^{\downarrow}(b))} \widetilde{Q}^3(k), \tag{S22}$$

where $\widetilde{Q}^1$, $\widetilde{Q}^2$ and $\widetilde{Q}^3$ are as defined in (S8)*,* (S9) *and* (S18)*, respectively.*

The proof is given in Section S2.9.

S1.8 Distribution of edge span

Under the SMC', for a given edge b, the distribution of its span can be characterised by considering the rate at which edge-disrupting recombination events arrive as we move left-to-right along the genome. However, the instantaneous rate at which b is disrupted at position τ may not be the same as that at position $\tau' > \tau$, due to the effect of other recombination events that might occur between τ and τ'. Thus, the span of an edge is the waiting time to the next edge-disrupting recombination event, but the rate at which this happens is inhomogeneous along the genome (and is, in fact, itself random).

Similarly to Deng et al. (2021), however, we find that if a recombination event between adjacent trees $\mathcal{T}$ and $\mathcal{T}'$ does not disrupt b, then

$$P_{\mathcal{T}}(b \text{ disrupted}) \cdot \mathcal{L}_{\mathcal{T}}(0) \approx P_{\mathcal{T}'}(b \text{ disrupted}) \cdot \mathcal{L}_{\mathcal{T}'}(0),$$

where $P_{\mathcal{T}}(b \text{ disrupted})$ is given by (S1), based on simulation results (Section S1.11). Thus, an approximation to the distribution of edge span can be constructed by assuming that the rate at which edge-disrupting recombination events arrive *is* homogeneous along the genome, which is equivalent to assuming that recombination events that do not disrupt the edge b also do not change the local tree: so if $\mathcal{T}$ is the local tree at position $d^{\leftarrow}(b)$, after each recombination event the newly formed local tree is $\mathcal{T}' = \mathcal{T}$. Recombination events occur as a Poisson process along the genome with rate $\mathcal{L}_{\mathcal{T}}(0) \cdot \rho/2$, allowing us to thin the process by multiplying this rate by the probability that the event is edge-disrupting, thereby offering a tractable approximation for the arrival of edge-disrupting recombination events. Then conditional on $d^{\leftarrow}(b)$, the edge span $d^{\rightarrow}(b) - d^{\leftarrow}(b)$ is distributed as the waiting time to the first event in a Poisson process with rate

$$\mathbb{P}_{\mathcal{T}}(b \text{ disrupted}) \cdot L_{\mathcal{T}}(0) \cdot \frac{\rho}{2}.$$

That is,

$$d^{\rightarrow}(b) - d^{\leftarrow}(b) \mid d^{\leftarrow}(b) \sim \text{Exp}\left(\mathbb{P}_{\mathcal{T}}(b \text{ disrupted}) \cdot L_{\mathcal{T}}(0) \cdot \frac{\rho}{2} \right),$$

or, by rescaling,

$$\mathbb{P}_{\mathcal{T}}(b \text{ disrupted}) \cdot L_{\mathcal{T}}(0) \cdot \frac{\rho}{2}(d^{\rightarrow}(b) - d^{\leftarrow}(b)) \mid d^{\leftarrow}(b) \sim \text{Exp}(1).$$

Analogously, if the recombination rate is not constant along the genome, with the population-scaled recombination rate at position w given by $\rho(w)/2$, then the intensity of the process at position w is instead given by

$$\mathbb{P}_{\mathcal{T}}(b \text{ disrupted}) \cdot L_{\mathcal{T}}(0) \cdot \frac{\rho(w)}{2},$$

and we have

$$\mathbb{P}_{\mathcal{T}}(b \text{ disrupted}) \cdot L_{\mathcal{T}}(0) \cdot \int_{d^{\leftarrow}(b)}^{d^{\rightarrow}(b)} \frac{\rho(w)}{2} dw \mid d^{\leftarrow}(b) \sim \text{Exp}\,(1)\,. \tag{S23}$$

The quality of this approximation can be verified by simulation, using the probability integral transform as follows. For the i-th edge $b_i \in \{b_1, \ldots, b_m\}$ of a simulated ARG, take $\mathcal{T}$ to be the local tree at position $d^{\leftarrow}(b_i)$, compute q_i as

$$q_i := \mathbb{P}_{\mathcal{T}}(b_i \text{ disrupted}) \cdot L_{\mathcal{T}}(0) \cdot \int_{d^{\leftarrow}(b_i)}^{d^{\rightarrow}(b_i)} \frac{\rho(w)}{2} dw,$$

and let $p_i = 1 - e^{-q_i}$. Then a Q-Q plot can be constructed by plotting the ordered quantities $p_{(1)} \leq \ldots \leq p_{(m)}$ against the corresponding quantiles of the uniform distribution $\frac{1}{1+m}, \ldots, \frac{m}{1+m}$. If the approximation fits well, the points should lie on the diagonal. A Kolmogorov–Smirnov (K–S) goodness of fit test can be used to test the null hypothesis that the computed p_i values are uniformly distributed.

We note that if any specific edge at a given position along the genome is selected, it may seem that its genomic span should be the sum of the waiting times to the left and to the right of the given position. This is the well-known "waiting time paradox" and we refer to Feller (1971, p. 12) for a thorough explanation.

S1.8.1 Considering only topology-disrupting events

If we were to consider only events that topologically disrupt the edge, we instead have the approximation

$$\mathbb{P}_{\mathcal{T}}(b \text{ topologically disrupted}) \cdot L_{\mathcal{T}}(0) \cdot \int_{d^{\leftarrow}(b)}^{d^{\rightarrow}(b)} \frac{\rho(w)}{2} dw \mid d^{\leftarrow}(b) \sim \text{Exp}\,(1)\,. \tag{S24}$$

A similar procedure to that described above can be used to check goodness of fit.

S1.8.2 Conditioning on edge having at least one mutation

ARG reconstruction algorithms utilise mutations to infer changes in local tree topologies due to recombination, so it may be of interest to consider only edges in reconstructed ARGs that are supported by at least one mutation. Suppose that mutations occur as a Poisson process along the edges with constant rate θ, and the recombination rate at position x is $\rho(x)/2$. Conditional on the left endpoint of the given edge $d^{\leftarrow}$, let D be its right endpoint, which using (S24) has the density

$$p^{D}_{\mathcal{T}}(\delta | d^{\leftarrow}) = \lambda(d^{\leftarrow}) \exp\left(- \int_{d^{\leftarrow}}^{\delta} \lambda(x) dx \right),$$

where

$$\lambda(x) = \mathbb{P}(b \text{ topologically disrupted}) \cdot L_{\mathcal{T}}(0) \cdot \frac{\rho(x)}{2}.$$

Let $\bar{t}$ be the time-length and M the number of mutations on the edge. Then the conditional distribution of D is given by

$$P_{\mathcal{T}}^{D}(d^{\rightarrow}|M>0,d^{\leftarrow}) = \frac{\int_{d^{\leftarrow}}^{d^{\rightarrow}} \mathbb{P}(M>0|\delta,d^{\leftarrow})p_{\mathcal{T}}^{D}(\delta|d^{\leftarrow})d\delta}{\int_{d^{\leftarrow}}^{\infty} \mathbb{P}(M>0|\delta,d^{\leftarrow})p_{\mathcal{T}}^{D}(\delta|d^{\leftarrow})d\delta},$$

with

$$\mathbb{P}(M>0|\delta,d^{\leftarrow}) = 1-\exp(-\theta\bar{t}(\delta-d^{\leftarrow})).$$

We have

$$\mathbb{P}(M>0|\delta,d^{\leftarrow})p_{\mathcal{T}}^{D}(\delta|d^{\leftarrow}) = \lambda(d^{\leftarrow})\exp\left(\int_{d^{\leftarrow}}^{\delta}\lambda(x)dx\right) - \lambda(d^{\leftarrow})\exp\left(-\theta\bar{t}(\delta-d^{\leftarrow}) - \int_{d^{\leftarrow}}^{\delta}\lambda(x)dx\right).$$

Assuming that the recombination map is piecewise constant, we split the part of the genome to the right of $d^{\leftarrow}$ into portions where the recombination rate is constant between the (ordered) breakpoints $d^{\leftarrow} =: w_0 < w_1 < w_2 < \ldots < w_k < \ldots$, adding an extra breakpoint $w_k := d^{\rightarrow}$. Then we can write

$$\begin{aligned}\int_{d^{\leftarrow}}^{\infty} \mathbb{P}(M>0|\delta,d^{\leftarrow})p_{\mathcal{T}}^{D}(\delta|d^{\leftarrow})d\delta &= 1-\lambda(d^{\leftarrow})\int_{d^{\leftarrow}}^{\infty}\exp\left(-\theta\bar{t}(\delta-d^{\leftarrow})-\int_{d^{\leftarrow}}^{\delta}\lambda(x)dx\right)d\delta \\ &= 1-\lambda(d^{\leftarrow})\sum_{i=0}^{\infty}\exp\left(-\int_{d^{\leftarrow}}^{w_i}\lambda(x)dx\right)\int_{w_i}^{w_{i+1}}\exp\left(-\theta\bar{t}(\delta-d^{\leftarrow})-\int_{w_i}^{\delta}\lambda(x)dx\right)d\delta \\ &= 1-\sum_{i=0}^{\infty}S_i,\end{aligned}$$

where, by integrating,

$$S_i = \frac{\lambda(d^{\leftarrow})\exp\left(-\int_{d^{\leftarrow}}^{w_i}\lambda(x)dx\right)\left[\exp(-\theta\bar{t}(w_i-d^{\leftarrow}))-\exp(-\theta\bar{t}(w_{i+1}-d^{\leftarrow})-\lambda(w_i)(w_{i+1}-w_i))\right]}{\theta\bar{t}+\lambda(w_i)}.$$

Similarly,

$$\int_{d^{\leftarrow}}^{d^{\rightarrow}} \mathbb{P}(M>0|\delta,d^{\leftarrow})p_{\mathcal{T}}^{D}(\delta|d^{\leftarrow})d\delta = 1-\exp\left(-\Lambda(b)\right)-\sum_{i=0}^{k-1}S_i,$$

where

$$\Lambda(b) = \int_{d^{\leftarrow}}^{d^{\rightarrow}}\lambda(x)dx.$$

Combining, we have

$$P_{\mathcal{T}}^{D}(d^{\rightarrow}|M>0,d^{\leftarrow}) = \frac{1-\exp(-\Lambda(b))-\sum_{j=0}^{k-1}S_j}{1-\sum_{j=0}^{\infty}S_j}. \tag{S25}$$

Note that in the limit $\theta\to\infty$, this reduces to $1-\exp\left(-\Lambda(b)\right)$, as expected (since this effectively removes the conditioning).

For the case where the recombination rate is constant along the genome, with $\rho(x)=\rho$ and $\lambda(x)=\lambda$, we obtain

$$P_{\mathcal{T}}^{D}(d^{\rightarrow}|M>0,d^{\leftarrow}) = 1-\frac{\theta\bar{t}+\lambda}{\theta\bar{t}}\exp(-\lambda(d^{\rightarrow}-d^{\leftarrow}))+\frac{\lambda}{\theta\bar{t}}\exp(-(\lambda+\theta\bar{t})(d^{\rightarrow}-d^{\leftarrow})).$$

The p-values for each edge of a simulated ARG can now be computed by evaluating the cdf (S25) for the given values of $d^{\leftarrow}$ and $d^{\rightarrow}$, and the Q-Q plot can again be constructed by plotting these against the corresponding quantiles of the uniform distribution. However, this potentially requires summing over a large number of increments of the recombination map. Instead, we propose to approximate (S25) by

$$\widetilde{P}_{\mathcal{T}}^{D}(d^{\rightarrow}|M>0,d^{\leftarrow}) := 1-\frac{\theta\bar{t}+\Lambda(b)}{\theta\bar{t}}\exp(-\Lambda(b))+\frac{\Lambda(b)}{\theta\bar{t}}\exp(-(\Lambda(b)+\theta\bar{t}d)).$$

We find this to be a very close match to the exact distribution based on simulations with human-like parameters, while being very fast to compute.

S1.9 Distribution of clade span

Similar approximations to those employed when investigating the distribution of edge span along the genome can be used for the distribution of the waiting time until a clade G is broken up by a recombination event (that is, when the clade either gains or loses one or more samples as a consequence of recombination). Consider an inhomogeneous Poisson process with intensity at position w given by

$$\mathbb{P}_{\mathcal{T}_{d^{\leftarrow}(G)}}(G \text{ disrupted}) \cdot L_{\mathcal{T}}(0) \cdot \frac{\rho(w)}{2}, \tag{S26}$$

conditional on the local tree at $d^{\leftarrow}(G)$ (defined as the leftmost position along the genome where the clade arises). Again through rescaling time, we have

$$\mathbb{P}_{\mathcal{T}_{d^{\leftarrow}(G)}}(G \text{ disrupted}) \cdot L_{\mathcal{T}_{d^{\leftarrow}(G)}}(0) \cdot \int_{d^{\leftarrow}(G)}^{d^{\rightarrow}(G)} \frac{\rho(w)}{2} dw \mid d^{\leftarrow}(G) \sim \operatorname{Exp}(1). \tag{S27}$$

Thus, for each clade G^i in a simulated ARG, we can calculate its left and right endpoints $d^{\leftarrow}(G^{(i)})$ and $d^{\rightarrow}(G^{(i)})$, respectively. Letting

$$q_i := \mathbb{P}_{\mathcal{T}_{d^{\leftarrow}(G^{(i)})}}(G^{(i)} \text{ disrupted}) \cdot L_{\mathcal{T}_{d^{\leftarrow}(G^{(i)})}}(0) \cdot \int_{d^{\leftarrow}(G^{(i)})}^{d^{\rightarrow}(G^{(i)})} \frac{\rho(w)}{2} dw, \tag{S28}$$

the quality of the approximation can again be checked using a Q-Q plot as described above.

S1.9.1 Adjusting for varying population size

Given a population size function $N(t)$, $t \geq 0$, let

$$\Lambda(t) = \int_0^t \frac{1}{N(t)} dt.$$

Let $\widetilde{L}_G(0)$ be the total length of branches in G measured in generations, and $\widetilde{L}_{\mathcal{T}}(0)$ the total branch length of $\mathcal{T}$ measured in generations. Then we have

$$\mathbb{P}(\mathcal{R} \in G) = \frac{\widetilde{L}_G(0)}{\widetilde{L}_{\mathcal{T}}(0)}, \quad \mathbb{P}(\mathcal{R} \notin G \cup g) = \frac{\widetilde{L}_{\mathcal{T}}(0) - \widetilde{L}_G(0) - \Lambda^{-1}(t^{\uparrow}(g)) + \Lambda^{-1}(t^{\downarrow}(g))}{\widetilde{L}_{\mathcal{T}}(0)}.$$

Conditional on the recombination point being on a branch within G, the density of the recombination time is

$$p_S(s|\mathcal{R} \in G) = \begin{cases} \frac{n_G(s)N(\Lambda^{-1}(s))}{\widetilde{L}_G(0)} & s \leq T_m \\ 0 & \text{otherwise,} \end{cases}$$

and similarly,

$$p_S(s|\mathcal{R} \notin G \cup g) = \begin{cases} \frac{(n(s)-n_{G\cup g}(s))N(\Lambda^{-1}(s))}{\widetilde{L}_{\mathcal{T}}(0)-\widetilde{L}_G(0)-\Lambda^{-1}(t^{\uparrow}(g))+\Lambda^{-1}(t^{\downarrow}(g))} & s \leq T_2 \\ 0 & \text{otherwise.} \end{cases}$$

Suppose now that $N(t)$ is piecewise constant, and for each $2 \leq k \leq n$, write

$$[T_{k+1}, T_k] = [\tau_k^0, \tau_k^1] \cup \ldots \cup [\tau_k^{\omega(k)-1}, \tau_k^{\omega(k)}],$$

where $\tau_0 := T_{k+1}$, $\tau_k^{\omega(k)} := T_k$, and $N(\tau) = N_k^v$ if $\tau \in [\tau_k^{v-1}, \tau_k^v]$. That is, $\omega(k)$ is the minimal number of (disjoint) intervals where the population size is piecewise constant, while there are k lineages in the tree. Then following similar calculations as in the proof of Theorem S1.2, we have

$$\mathbb{P}(\mathcal{C} \notin G \cup g|\mathcal{R} \in G)\mathbb{P}(\mathcal{R} \in G) = \frac{\widetilde{L}_G(0)}{\widetilde{L}_{\mathcal{T}}(0)} - \frac{1}{\widetilde{L}_{\mathcal{T}}(0)} \sum_{k=n(t^{\downarrow}(g))+1}^{n} n_G(T_{k+1}) \cdot$$

$$\cdot \left[n_{G\cup g}(T_{k+1})\widetilde{Q}^1(k)\frac{\widetilde{T}_k - \widetilde{T}_{k+1}}{T_k - T_{k+1}} + \frac{1}{k}\left(\sum_{j=n(t^{\uparrow}(g))+1}^{k} n_{G\cup g}(T_{j+1})Q_{kj}\right)\sum_{i=1}^{\omega(k)} N_k^i \left(e^{k\tau_k^i} - e^{k\tau_k^{i-1}}\right)\right],$$

where $\widetilde{T}_j = \Lambda^{-1}(T_j)$, and

$$\mathbb{P}(\mathcal{C} \in G|\mathcal{R} \notin G \cup g)\mathbb{P}(\mathcal{R} \notin G \cup g) = \frac{1}{\widetilde{L}_{\mathcal{T}}(0)} \sum_{k=n(t^{\downarrow}(g))+1}^{n} [k - n_{G\cup g}(T_{k+1})] \cdot$$
$$\cdot \left[n_G(T_{k+1})\widetilde{Q}^1(k)\frac{\widetilde{T}_k - \widetilde{T}_{k+1}}{T_k - T_{k+1}} + \frac{1}{k}\left(\sum_{j=n(t^{\downarrow}(g))+1}^{k} n_G(T_{j+1})Q_{kj}\right)\sum_{i=1}^{\omega(k)} N_k^i \left(e^{k\tau_k^i} - e^{k\tau_k^{i-1}}\right)\right],$$

which can be substituted into (S13), and in turn into the expressions in Section S1.9, to give the corresponding approximation to the genomic span of G under an arbitrary piecewise constant population size model. This can also be applied to an arbitrary population size model, through averaging the population size over a suitable time grid and thus approximating it with a piecewise constant function.

S1.10 Quality of approximation to the distribution of edge span

We first assess the quality of the approximation derived in Section S1.8 by simulating an ARG under the SMC' and checking if the simulated edge spans follow (S23), by using the procedure described in Section S1.8. The simulation parameters are given in Section 4.6.1 (main text), and we sampled 10 000 edges from each ARG (uniformly at random) for testing, to speed up computation. The corresponding Q-Q plots are shown in Figure S4 (blue points). The points adhere very closely to the diagonal, demonstrating that the approximation provides an excellent fit. The K–S p-values of 0.31 (left panel) and 0.75 (right panel) also suggest good agreement. Grouping edges by their depth (the number of edges on the way to the MRCA) or clade size (number of samples subtended by the edge) in the tree and constructing Q-Q plots for each group also did not reveal significant deviation from the diagonal (Figure S14), suggesting that the approximation holds for all edges.

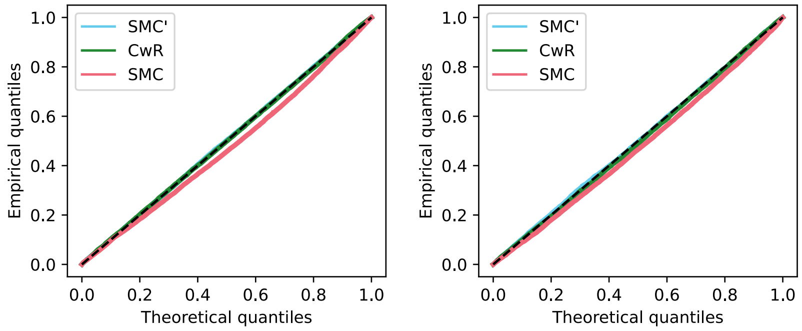

Figure S4: Q-Q plots using (S23) computed from ARGs simulated using the SMC' (blue), CwR (green), and SMC (red) models with $n = 100$. Note the blue and green points closely overlap, and overlay the diagonal. Left panel: dataset 1 parameters; right panel: dataset 2 parameters. Dashed line: diagonal from $(0, 0)$ to $(1, 1)$.

S1.11 Effects of recombination on a local tree

The very good quality of the approximation above can be understood by considering the effects of a recombination event on properties of the local tree.

For a given edge b_i that exists in local trees $\mathcal{T}_{(1)}, \ldots, \mathcal{T}_{(k)}$, let

$$f(b_i, l) := P_{\mathcal{T}_{(l)}}(b_i \text{ disrupted}) \cdot \mathcal{L}_{\mathcal{T}_{(l)}}(0),$$

for $1 \le l \le k$. Let

$$H_1^l(b_i) := f(b_i, l+1)/f(b_i, l),$$

for $1 \le l \le k-1$, and

$$H_2(b_i) = \max_l f(b_i, l)/\min_l f(b_i, l).$$

We calculate these quantities for a uniform random sample of 1 000 edges from an ARG simulated using dataset 1 parameters in Section 4.6.1 (Main Text); the corresponding histograms are shown in Figure S5. These suggest that the quantity $P_{\mathcal{T}}(b_i \text{ disrupted}) \cdot \mathcal{L}_{\mathcal{T}}(0)$ stays relatively conserved following each recombination event, even if individually $P_{\mathcal{T}}(b_i \text{ disrupted})$ and $\mathcal{L}_{\mathcal{T}}(0)$ may vary more significantly. This is similar to the findings of Deng et al. (2021, Figure 5).

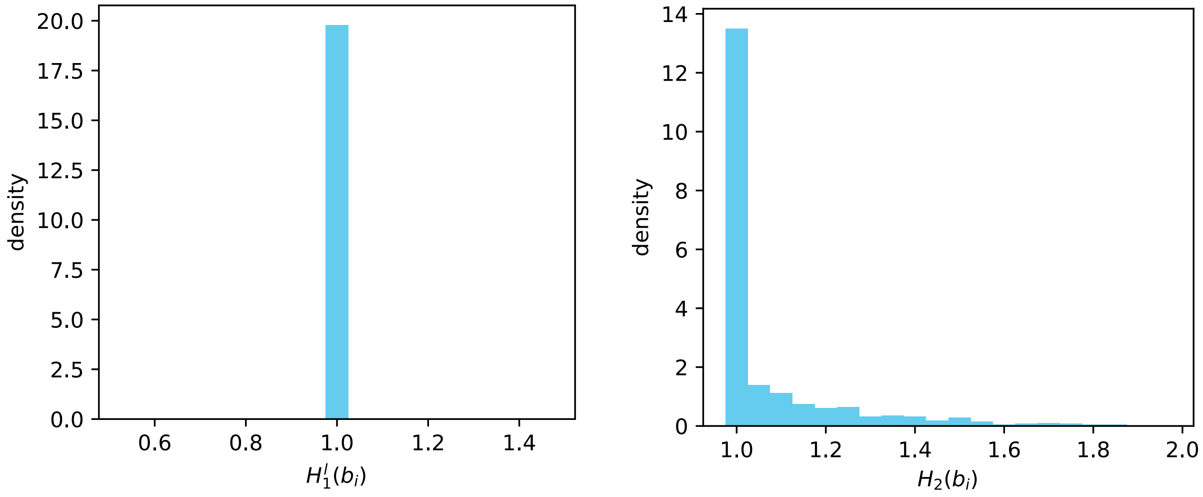

Figure S5: Histograms of $H_1^l(b_i)$ and $H_2(b_i)$ for a simulated ARG.

To calculate a Monte Carlo estimate of the marginal probability that the change in total branch length is negative, zero, or positive, we average over local trees simulated using msprime under the SMC' model. The results are shown in Figure S6. Recombination has a stabilising effect on the total branch length of local trees: when the total branch length is small (resp. large), the probability that the recombination event will increase the total branch length increases (resp. decreases).

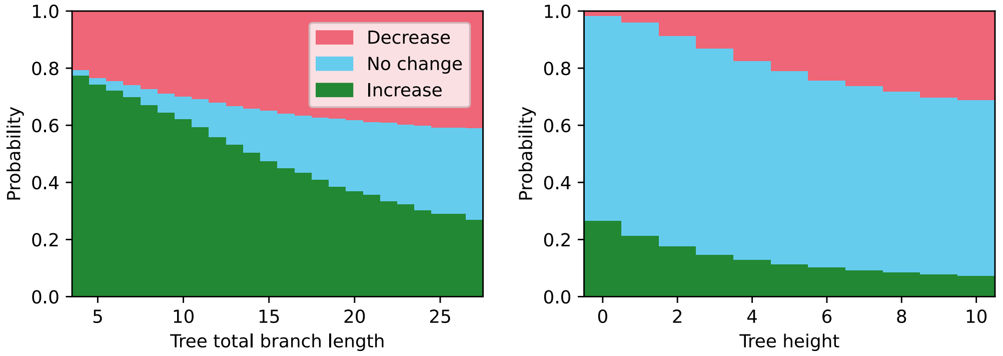

Figure S6: Mean probability of change in total branch length (left) and tree height (right) being negative (red, top stack), zero (blue, middle stack) or positive (green, bottom stack). Trees were simulated and binned according to total branch length (left) or height (right), with 100 trees simulated per bin, and sample size $n = 100$. For each tree, probabilities were calculated using (S15)-(S17), the stacked bar plot shows the mean probabilities for each bin.

Similarly, we can average over trees simulated under the SMC' model to estimate the marginal probability that the change in tree height following a recombination event is negative, zero, or positive. The results are shown in Figure S6 (right panel). As with total branch length, recombination

tends to increase (resp. decrease) the tree height with higher probability when the tree height is small (resp. large).

Figure S7 shows the density (S19) for three simulated trees with varying total branch lengths $L_{\mathcal{T}}(0)$, for $n = 10$ and $n = 100$. In both cases, the density is concentrated around zero and skewed to the left (resp. right) when the total branch length is small (resp. large); it is roughly symmetric about zero for middling values of $L_{\mathcal{T}}(0)$.

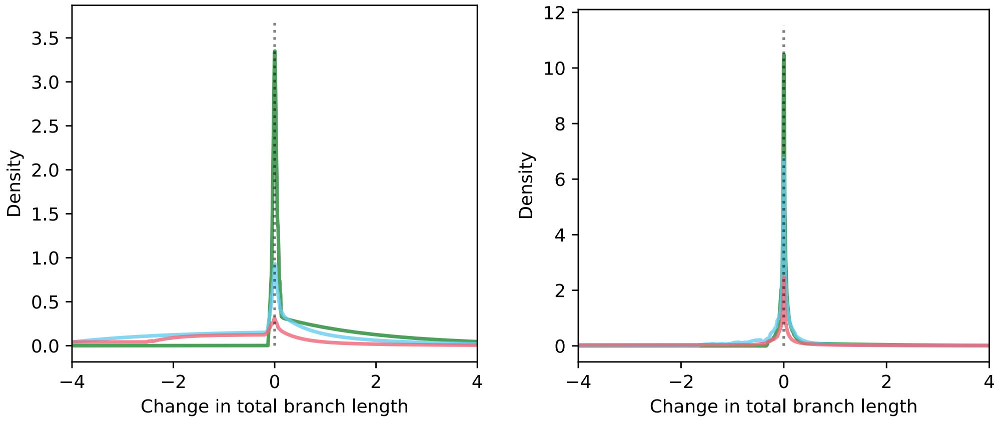

Figure S7: Density of change in total branch length (S19) for three simulated trees, with $n = 10$ (left panel) $n = 100$ (right panel). Left: trees have total branch length 1 (green), 6 (blue) and 24 (red). Right: trees have total branch length 5 (green), 10 (blue) and 28 (red).

These results shed light on why, despite the strong assumption that recombination events that do not disrupt the given edge also do not change the rest of the local tree, our approximation to the distribution of edge span gives an extraordinarily close fit for data simulated under the SMC' model. For an edge that is close to the leaves, the probability that the edge is disrupted by the next recombination event is very small (Figure 2). Thus, many recombination events will occur before this edge is disrupted. As can be seen in Figure S6, recombination has the effect of stabilising the total branch length, with events causing an increase (decrease) in total branch length being more likely if the current total branch length is relatively small (large). Thus, it seems the fluctuations in total branch length average out and do not significantly affect the overall rate of edge-disrupting recombination events. On the other hand, for an edge that is close to the root of the tree, per Figure 2 the probability of the edge being disrupted by the next recombination is relatively high. Thus, a relatively small number of recombination events are likely to occur before they affect the given edge. As illustrated in Figure S7, when a recombination changes the total branch length of the tree, the magnitude of this change is concentrated around 0. Thus, the effect of recombination on the rest of the tree does not appear to significantly affect the probability that the edge is disrupted.

This applies at the level of each individual edge, so after rescaling each observed edge span by its specific event rate as per (S23), these rescaled edge spans follow an Exp(1) distribution. Accounting for mutiple testing using a Bonferroni correction, we can thus use the resulting p-values to detect outlier edges with longer-than-expected spans. The same reasoning applies for the genomic spans of clades.

S1.12 Comparison of simulation models

We next simulate ARGs under the CwR and under the SMC, with the same two parameter settings given in Section 4.6.1 (main text) and again compare the resulting edge spans to (S23). For the CwR, the span of an edge is taken to be the sum of all the genomic intervals where that edge appears in the local tree (to account for the presence of recombination events that occur in non-ancestral material). Figure S4 shows that the approximation is an excellent fit to the CwR (green points), with K–S p-values of 0.06 (left panel) and 0.94 (right panel). This suggests that the distribution of edge spans under the SMC' and that under the CwR are remarkably close.

Under the SMC (red points), edge spans are shorter in general, with points falling below the diagonal (with both K–S p-values < 0.001). This is due to the model disallowing recombination events that do not change the local tree, so edges are more frequently disrupted by recombination.

S1.13 Detection of local recombination suppression: Test 1

Given an ARG, for each clade $G^{(i)}$, we calculate its left and right endpoints $d^{\leftarrow}(G^{(i)})$ and $d^{\rightarrow}(G^{(i)})$, and would like to estimate the probability of observing a clade span greater than $d^{\rightarrow}(G^{(i)}) - d^{\leftarrow}(G^{(i)})$ via $p_i = e^{-q_i}$, with q_i as defined in (S28). This is a one-sided p-value, and we test whether $G^{(i)}$ has a significantly longer span than otherwise expected by comparing this against a Bonferroni-corrected significance threshold (0.05 divided by the total number of tested clades). Simulation studies confirm that for ARGs simulated under the SMC' model without inversions, these p-values are approximately uniformly distributed (Figure 4, left panel, blue points), as expected.

S1.13.1 Reconstructed ARGs

As can be seen from Figure 4 (left panel), similarly to edge span, the distribution of clade span in ARGs reconstructed using Relate, tsinfer/tsdate and ARG-Needle is skewed, with clade span generally overestimated by these methods. Thus, directly applying the test as described above will lead to a high false positive rate. We now describe a correction which can be applied to counteract two main problematic features of reconstructed ARGs, focusing particularly on Relate (due to the presence of polytomies for tsinfer/tsdate being difficult to correct for, and the large bias seen with ARG-Needle which both under- and overestimates clade span).

Let $\mathcal{G} = \{G_1, G_2, \ldots, G_N\}$ be a list of all clades in the reconstructed ARG. The first issue is that due to a lack of mutations around the leftmost and rightmost endpoints of a clade, Relate may overestimate its span, causing false positives. To correct for this, we proceed as follows. Suppose that the root edges of a clade $G^{(i)}$ in trees $\mathcal{T}_{d^{\leftarrow}(G^{(i)})}, \ldots, \mathcal{T}_{d^{\rightarrow}(G^{(i)})}$ have mutations at positions $m_1^i < m_2^i < \ldots < m_K^i$. We (1) remove from $\mathcal{G}$ all clades with fewer than three mutations in total and fewer than M mutations per kb on average, and (2) measure an adjusted clade span $\widetilde{d}$ using the positions of the leftmost and rightmost mutations that support the given clade. That is, we define

$$\widetilde{d}^{\leftarrow}(G^{(i)}) = m_1^i, \ \widetilde{d}^{\rightarrow}(G^{(i)}) = m_K^i.$$

The second issue is that the clade carrying the inversion may not be supported by mutations uniformly along the inverted region, causing it to appear and disappear multiple times in quick succession in the reconstructed ARG, which can cause false negatives. We correct for this by "merging" pairs of clades that are nearby on the genome (in terms of genetic distance, to allow for varying recombination rates). For two clades $G^{(i)}, G^{(j)} \in \mathcal{G}$ that have identical sets of sample descendants and are less than L cM apart, that is

$$100 \cdot \int_{d^{\rightarrow}(G^{(i)})}^{d^{\leftarrow}(G^{(j)})} \rho(w)dw < L,$$

form $G_{i,j} := G^{(i)}$ but setting $d^{\leftarrow}(G^{(i,j)}) := d^{\leftarrow}(G^{(i)})$, $d^{\rightarrow}(G^{(i,j)}) := d^{\rightarrow}(G^{(j)})$, $\widetilde{d}^{\leftarrow}(G^{(i,j)}) := \widetilde{d}^{\leftarrow}(G^{(i)})$, $\widetilde{d}^{\rightarrow}(G^{(i,j)}) := \widetilde{d}^{\rightarrow}(G^{(j)})$, and update $\mathcal{G}$ as

$$\mathcal{G} = \{G^{(i,j)}\} \cup \mathcal{G} \backslash \{G^{(i)}, G^{(j)}\}.$$

We apply this to all pairs of clades in $\mathcal{G}$ iteratively, until no more clades can be merged together. We note that this correction also helps to handle the presence of gene conversion within inverted regions, which can be commonplace (Korunes and Noor, 2019; Crown et al., 2018): a gene conversion will result in a short stretch of the genome where the clade is disrupted but then reappears, and the described adjustment will ensure that this does not affect the calculated clade span.

For each clade in the reduced list $G^{(i)} \in \mathcal{G}$, we thus calculate an adjusted version of (S28):

$$\widetilde{q}_i := \mathbb{P}_{\mathcal{T}_{\widetilde{d}^{\leftarrow}(G^{(i)})}}(G^{(i)} \text{ disrupted}) \cdot L_{\mathcal{T}_{\widetilde{d}^{\leftarrow}(G^{(i)})}}(0) \cdot \int_{\widetilde{d}^{\leftarrow}(G^{(i)})}^{\widetilde{d}^{\rightarrow}(G^{(i)})} \frac{\rho(w)}{2} dw, \tag{S29}$$

again taking $\widetilde{p}_i = e^{-\widetilde{q}_i}$. Note that with the above definitions, this can be computed even though the clades in the reduced list will now not necessarily exist (with the re-defined spans) in the ARG itself. We thus obtain adjusted p-values for each clade, applying a significance threshold of $0.05/N$, where N is the original number of clades in the reconstructed ARG.

We apply these corrections to the ARG reconstructed using Relate for the data simulated using SLiM (as described in Section 4.6.2), setting $L = 0.01$ and $M = 0.05$. The resulting Q-Q and p-value plots are shown in the top row of Figure S18, showing that the correction brings the points on the Q-Q plot very close to the diagonal, and there are three significant clades (all of which overlap the inverted region, and the clade spanning the entire inverted region remains a significant outlier with the lowest p-value). The equivalent plots for a simulation with no inversion are shown in the bottom row of Figure S18, demonstrating that the p-values are approximately uniformly distributed and there are no false positives.

The choice of the parameter L influences power and the rate of false positives, which will both increase as L increases, since the merging procedure lengthens clade spans. We construct a bound on the false positive rate using the following approximation. For a given set of S sequences, the probability that these form a clade in a random coalescent tree of size n is

$$\frac{2}{(S+1)\binom{n-1}{S-1}}$$

(e.g. Hein et al., 2004, p. 84, eq. 3.26). This probability is very small unless S is small or, by symmetry, close to n. Thus, for a given clade G of size S, the probability that the clade is broken up by recombination at position $d^{\rightarrow}(G)$ but then appears again within L cM (purely due to random chance) is bounded above by

$$\frac{2W}{(S+1)\binom{n-1}{S-1}},$$

where W is the number of trees within L cM of $d^{\rightarrow}(G)$ (and further requiring that the clade is supported by at least one mutation results in a smaller bound).

For the 1KGP data, there are $\approx$ 2m trees in total along the genome. With $n = 100$, setting $L = \infty$ (so $W \leq 2\,000\,000$), gives an upper bound of $2 \cdot 10^{-7}$ on the probability that a clade of size $S = 10$ reappears by random chance anywhere along the genome. Thus, even if 10m clades of size 10 are considered, we expect at most two false positives to arise due to the merging procedure. The rate of false positives decreases as n (and S) increase; with $n = 1000$ and $S = 10$ the upper bound falls to $1 \cdot 10^{-16}$. In conclusion, choosing L to be large only slightly increases the rate of false positives, while increasing power.

S1.13.2 Test error rates

We examined the performance of the test by applying it to ARGs simulated using SLiM with the parameters given in Section 2.4.1 (with inversions at intermediate frequency, on average 50%), varying the length of the inverted region from 0 to 200kb (100 ARGs in each case). Defining positive detection as there being at least one clade within the inverted region with a significant p-value, the resulting ROC curve is shown in Figure S8 (left panel). Fixing the false positive rate at 5% (corresponding roughly to one false positive per 100Mb) gives the confusion matrix shown in Table 1a, demonstrating very high sensitivity. For each inversion length, out of all the clades with significant p-values across the simulations, a high percentage lie within the inverted region.

Reconstructing an ARG using Relate for each simulated dataset and applying the adjustments described in Section 2.4.2 gives the ROC curve shown in Figure S8 (right panel); the results in Table 1b show high sensitivity is maintained for inversions longer than around 100kb. These results

demonstrate very good performance in detecting the presence of inversions, as well as pinpointing the candidate clade and its position along the genome.

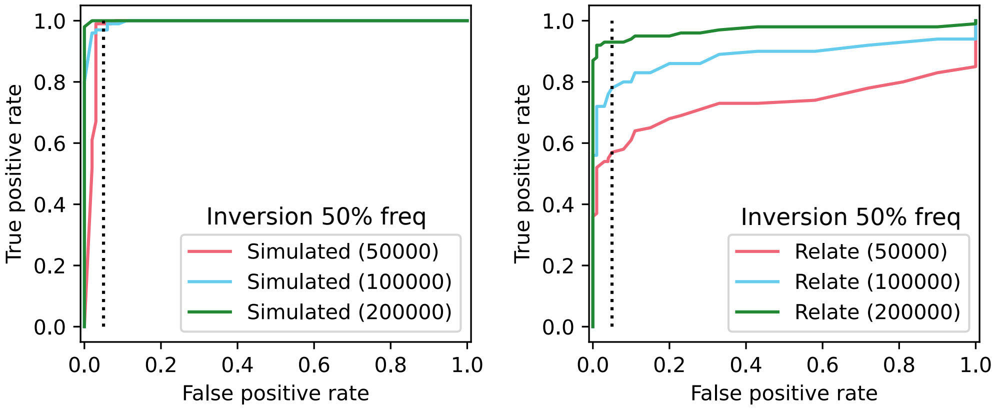

Figure S8: ROC curve for inversion detection test, based on 100 simulations for each given length of the inverted region. Left: using the simulated ARGs; right: ARGs reconstructed using Relate. Dotted line corresponds to a false positive rate of 5% (false positive being defined as an ARG simulated with no inversion but having at least one significant clade in the region). Colours correspond to the different inversion lengths.

(a)

		Inversion length (kb)			
		200	100	50	0
Inv. detected	+	100	97	99	5
	−	0	3	1	95
Clades tested		3.3m	3.4m	3.4m	3.4m
Significant clades		201	144	127	5
Within inv. region		96%	92%	97%	0%

(b)

		Inversion length (kb)			
		200	100	50	0
Inv. detected	+	93	78	57	5
	−	7	22	43	95
Clades tested		116k	116k	117k	122k
Significant clades		227	123	79	5
Within inv. region		96%	97%	96%	0%

Table 1: Confusion matrices and results summaries for inversion detection test, based on 100 simulations for each given length of the inverted region: using (a) the simulated ARGs, (b) ARGs reconstructed using Relate.

To investigate how performance depends on inversion frequency for reconstructed ARGs, we further simulated the same scenario but with the inversion at 10% and 20% average frequency. The resulting ROC curves are shown in Figure S9. As expected, power decreases with decreasing inversion frequency, since smaller clades are expected to have shorter genomic spans, so it is more difficult to detect them as outliers.

S1.13.3 Comparison to other methods

We compared the performance of our test in predicting inversion genotypes against that of invClust (Cáceres and González, 2015), a method based on clustering haplotypes using multidimensional scaling of SNPs. We ran simulations using SLiM with varying inversion sizes, as described in Section S1.13.2. Since invClust requires the candidate location of the inversion, we gave the true simulated position as this input (using a larger region containing the inversion gave the same results, and using regions not overlapping with the inversion gave very poor performance, as can be expected). We then used invClust to predict inversion genotypes (homozygous non-carrier, heterozygous, or homozygous carrier) and calculated the squared correlation with the simulated ground truth. We also predicted inversion genotypes using our method, by considering the sequences within the top significant clade. The results are presented in Figure S10 (left panel), showing that our method achieves very high prediction accuracy, consistently outperforming invClust for all simulated inversion sizes.

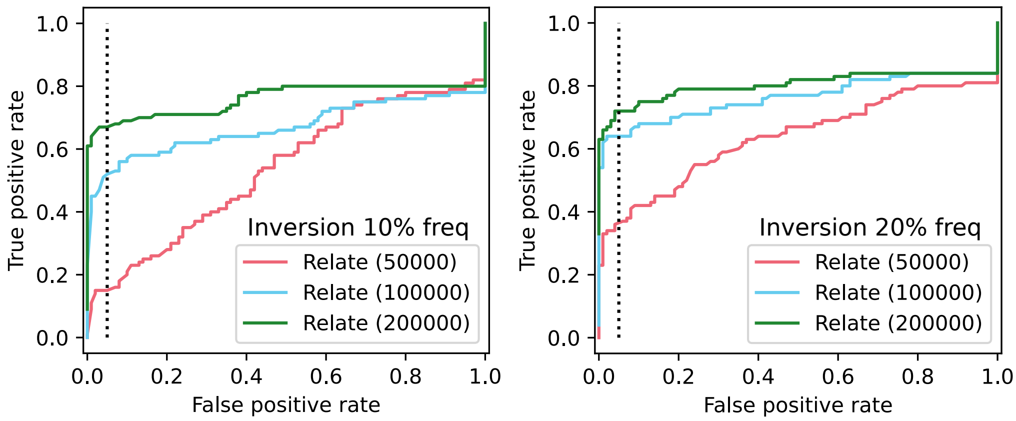

Figure S9: ROC curve for inversion detection test, based on 100 simulations for each given length of the inverted region. Left: inversion at 10% average frequency. Right: inversion at 20% average frequency.

We also calculated an accuracy score for how well our method predicts the location of the inverted region (by calculating the proportion of overlap between the span of the top significant clade and the true simulated region). A histogram of this is shown in Figure S10 (right panel), demonstrating very good accuracy, with the predicted region overlapping more than half of the true region in 81% of simulations. In both of these comparisons (and in the ROC curves in Figures S8 and S9), it can be seen that the performance of our method improves as the size of the inversion increases.

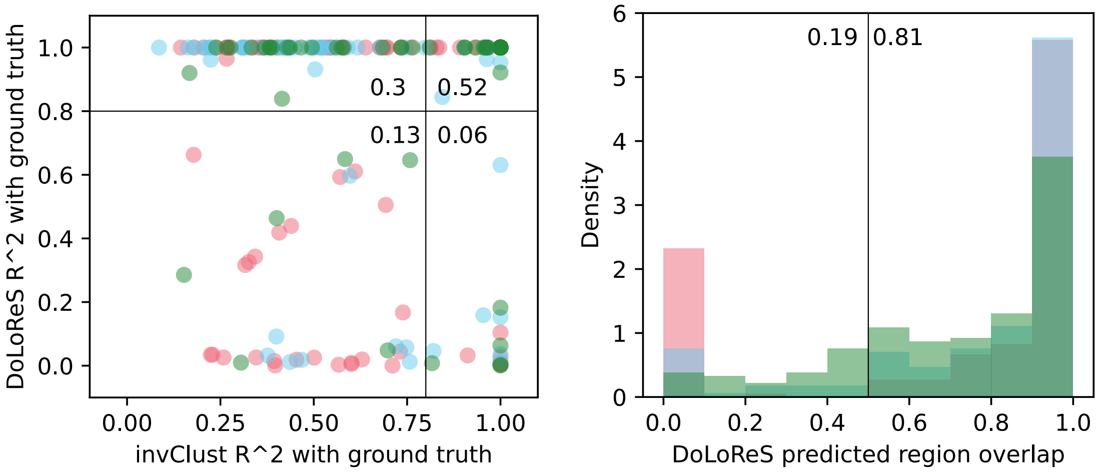

Figure S10: Left panel: comparison of performance against invClust, based on 100 simulations for each given length of the inverted region (colours correspond to region length as in Figure S8). Points show squared correlation between true and predicted inversion carriers. Numbers show proportion of points falling in each quadrant. Right panel: histogram of proportion of overlap between predicted and true inverted region.

We also compared the performance of our method in predicting genotypes and inversion positions against Asaph (Nowling et al., 2022), a method using PCA to detect and localise inversions, but found Asaph to perform very poorly on our simulated data. This is likely because the focus of Asaph is on scalability and the detection of very large and old inversions.

S1.14 Detection of local recombination suppression: Test 2

Under our approximation, recombination events arrive as an inhomogeneous Poisson process along the genome with rate $\lambda(w)$ given by (S26). For a particular clade G, call recombination events which do not change the membership of G "Type 1", and other events "Type 2" (our key

assumption is that Type 1 events also don't change the local trees). We thus have Type 1 events arriving at rate $z\lambda(w)$, and Type 2 events at rate $(1-z)\lambda(w)$, where $z := \mathbb{P}_{\mathcal{T}_{d^{\leftarrow}(G)}}(G \text{ disrupted})$. Let D be the number of Type 1 events before the first Type 2 event. Then it is easy to show that the marginal distribution of D is geometric with parameter z.

Thus, if G is first disrupted by the R-th recombination event, we can calculate a corresponding p-value as

$$p_i = \left[1 - \mathbb{P}_{\mathcal{T}_{d^{\leftarrow}(G)}}(G \text{ disrupted})\right]^{R-1}.$$

If R is known exactly, this is equivalent to Test 1.

References

Cáceres, A. and González, J. R. Following the footprints of polymorphic inversions on SNP data: From detection to association tests. *Nucleic Acids Research*, **43**(8): e53–e53, 2015.

Carmi, S., Wilton, P. R., Wakeley, J., and Pe'er, I. A renewal theory approach to IBD sharing. *Theoretical Population Biology*, **97**: 35–48, 2014.

Crown, K. N., Miller, D. E., Sekelsky, J., and Hawley, R. S. Local inversion heterozygosity alters recombination throughout the genome. *Current Biology*, **28**(18): 2984–2990, 2018.

Deng, Y., Song, Y. S., and Nielsen, R. The distribution of waiting distances in ancestral recombination graphs. *Theoretical Population Biology*, **141**: 34–43, 2021.

Eriksson, A., Mahjani, B., and Mehlig, B. Sequential Markov coalescent algorithms for population models with demographic structure. *Theoretical Population Biology*, **76**(2): 84–91, 2009.

Feller, W. *An introduction to probability theory and its applications, Volume 2.* John Wiley & Sons, 2 edn., 1971.

Griffiths, R. C. and Marjoram, P. An ancestral recombination graph. In P. Donnelly and S. Tavaré, eds., *Progress in population genetics and human evolution*, 257–270. Springer, New York, 1997.

Harris, K. and Nielsen, R. Inferring demographic history from a spectrum of shared haplotype lengths. *PLOS Genetics*, **9**(6): e1003521, 2013.

Hein, J., Schierup, M., and Wiuf, C. *Gene genealogies, variation and evolution: A primer in coalescent theory.* Oxford University Press, USA, 2004.

Hejase, H. A., Mo, Z., Campagna, L., and Siepel, A. A deep-learning approach for inference of selective sweeps from the ancestral recombination graph. *Molecular Biology and Evolution*, **39**(1): msab332, 2022.

Hobolth, A. and Jensen, J. L. Markovian approximation to the finite loci coalescent with recombination along multiple sequences. *Theoretical Population Biology*, **98**: 48–58, 2014.

Hudson, R. R. Properties of a neutral allele model with intragenic recombination. *Theoretical Population Biology*, **23**(2): 183–201, 1983.

Kelleher, J., Wong, Y., Wohns, A. W., Fadil, C., Albers, P. K., and McVean, G. Inferring whole-genome histories in large population datasets. *Nature Genetics*, **51**(9): 1330–1338, 2019.

Korunes, K. L. and Noor, M. A. Pervasive gene conversion in chromosomal inversion heterozygotes. *Molecular Ecology*, **28**(6): 1302–1315, 2019.

Li, H. and Durbin, R. Inference of human population history from individual whole-genome sequences. *Nature*, **475**(7357): 493–496, 2011.

Li, N. and Stephens, M. Modeling linkage disequilibrium and identifying recombination hotspots using single-nucleotide polymorphism data. *Genetics*, **165**(4): 2213–2233, 2003.

Marjoram, P. and Wall, J. D. Fast coalescent simulation. *BMC Genetics*, **7**(1): 1–9, 2006.

McVean, G. A. and Cardin, N. J. Approximating the coalescent with recombination. *Philosophical Transactions of the Royal Society B: Biological Sciences*, **360**(1459): 1387–1393, 2005.

Nowling, R. J., Fallas-Moya, F., Sadovnik, A., Emrich, S., Aleck, M., Leskiewicz, D., and Peters, J. G. Fast, low-memory detection and localization of large, polymorphic inversions from SNPs. *PeerJ*, **10**: e12831, 2022.

Rasmussen, M. D., Hubisz, M. J., Gronau, I., and Siepel, A. Genome-wide inference of ancestral recombination graphs. *PLOS Genetics*, **10**(5): e1004342, 2014.

Schiffels, S. and Durbin, R. Inferring human population size and separation history from multiple genome sequences. *Nature Genetics*, **46**(8): 919–925, 2014.

Speidel, L., Forest, M., Shi, S., and Myers, S. R. A method for genome-wide genealogy estimation for thousands of samples. *Nature Genetics*, **51**(9): 1321–1329, 2019.

Wilton, P. R., Carmi, S., and Hobolth, A. The SMC' is a highly accurate approximation to the ancestral recombination graph. *Genetics*, **200**(1): 343–355, 2015.

Wiuf, C. and Hein, J. Recombination as a point process along sequences. *Theoretical Population Biology*, **55**(3): 248–259, 1999.

Wohns, A. W., Wong, Y., Jeffery, B., Akbari, A., Mallick, S., Pinhasi, R., Patterson, N., Reich, D., Kelleher, J., and McVean, G. A unified genealogy of modern and ancient genomes. *Science*, **375**(6583): eabi8264, 2022.

Zhang, B. C., Biddanda, A., Gunnarsson, Á. F., Cooper, F., and Palamara, P. F. Biobank-scale inference of ancestral recombination graphs enables genealogical analysis of complex traits. *Nature Genetics*, **55**: 768–776, 2023.

S2 Proofs

S2.1 Proof of Proposition S1.1

Conditioning on the recombination happening on edge β, the density of the recombination event time is

$$p^S_{\mathcal{T}}(s|\mathcal{R} \in \beta) = \begin{cases} \frac{1}{t(\beta)} & t^{\downarrow}(\beta) \leq s \leq t^{\uparrow}(\beta) \\ 0 & \text{otherwise,} \end{cases}$$

as the recombination time is chosen uniformly at random along the length of the edge. The conditional density of the coalescence time is

$$p^U_{\mathcal{T}}(u|\mathcal{R} = (\beta, s)) = p^U_{\mathcal{T}}(u|\mathcal{R} = (\cdot, s)) = n(u) \exp\left(-\int_s^u n(t)dt\right), \tag{S1}$$

for $u > s$ and 0 otherwise. Conditional on the coalescence time u, the probability that the coalescence point is on β is

$$\mathbb{P}_{\mathcal{T}}(\mathcal{C} \in \beta|\mathcal{C} = (\cdot, u), \mathcal{R} = (\beta, s)) = \begin{cases} \frac{1}{n(u)} & s < u < t^{\uparrow}(\beta) \\ 0 & \text{otherwise.} \end{cases}$$

Letting $k = n(s)$, so that T_k is the first coalescence time just above time s,

$$\begin{aligned} \mathbb{P}_{\mathcal{T}}(\mathcal{C} \in \beta|\mathcal{R} = (\beta, s)) &= \int_0^{\infty} \mathbb{P}(\mathcal{C} \in \beta|\mathcal{C} = (\cdot, u), \mathcal{R} = (\beta, s)) \cdot p^U_{\mathcal{T}}(u|\mathcal{R} = (\cdot, s))du \\ &= \int_s^{t^{\uparrow}(\beta)} \exp\left(-\int_s^u n(t)dt\right) du \\ &= \int_s^{T_k} \exp\left(-\int_s^u k\,dt\right) du + \sum_{j=n(t^{\uparrow}(\beta))+1}^{k-1} \int_{T_{j+1}}^{T_j} \exp\left(-\int_s^u n(t)\,dt\right) du, \end{aligned}$$

note that $T_{n(t^{\uparrow}(b))+1} = t^{\uparrow}(b)$. The first term is

$$\begin{aligned} \int_s^{T_k} \exp\left(-\int_s^u k\,dt\right) du &= \int_s^{T_k} \exp\left(-k(u-s)\right) du \\ &= \left[-\frac{1}{k}e^{-k(u-s)}\right]_s^{T_k} \\ &= \frac{1}{k} - \frac{1}{k}e^{-kT_k}e^{ks}, \end{aligned} \tag{S2}$$

and the summands of the second term are

$$\begin{aligned} \int_{T_{j+1}}^{T_j} \exp\left(-\int_s^u n(t)\,dt\right) du &= \int_{T_{j+1}}^{T_j} \exp\left(-\int_s^{T_{j+1}} n(t)\,dt - \int_{T_{j+1}}^u n(t)\,dt\right) du \\ &= \exp\left(-\int_s^{T_{j+1}} n(t)\,dt\right) \int_{T_{j+1}}^{T_j} \exp\left(-\int_{T_{j+1}}^u j\,dt\right) du \\ &= \exp\left(-k(T_k - s) - \sum_{i=j+1}^{k-1} i(T_i - T_{i+1})\right) \left[-\frac{1}{j}e^{-j(u-T_{j+1})}\right]_{T_{j+1}}^{T_j} \\ &= e^{ks} \exp\left(-kT_k + L_{\mathcal{T}}(T_{j+1}) - L_{\mathcal{T}}(T_k)\right) \frac{1}{j}\left(1 - e^{-j(T_j - T_{j+1})}\right). \end{aligned}$$

Thus,

$$\mathbb{P}_{\mathcal{T}}(\mathcal{C} \in \beta|\mathcal{R} = (\beta, s)) = \frac{1}{k} + e^{ks} \cdot \sum_{j=n(t^{\uparrow}(\beta))+1}^{k} Q_{kj},$$

where

$$Q_{kk} := -\frac{1}{k} e^{-kT_k},$$

and

$$\begin{aligned}
Q_{kj} &:= \frac{1}{j}\left(1 - e^{-j(T_j - T_{j+1})}\right)\exp\left(-kT_k + L_{\mathcal{T}}(T_{j+1}) - L_{\mathcal{T}}(T_k)\right) \\
&= \frac{1}{j} e^{-kT_k} e^{-L_{\mathcal{T}}(T_k)}\left(e^{L_{\mathcal{T}}(T_{j+1})} - e^{L_{\mathcal{T}}(T_{j+1}) - j(T_j - T_{j+1})}\right) \\
&= e^{-kT_k} e^{-L_{\mathcal{T}}(T_k)} \frac{1}{j}\left(e^{L_{\mathcal{T}}(T_{j+1})} - e^{L_{\mathcal{T}}(T_j)}\right).
\end{aligned}$$

S2.2 Proof of Proposition S1.2

Marginalising out the recombination time in (S4),

$$\begin{aligned}
\mathbb{P}_{\mathcal{T}}(\mathcal{C} \notin \beta | \mathcal{R} \in \beta) &= 1 - \int_{t^{\downarrow}(\beta)}^{t^{\uparrow}(\beta)} \mathbb{P}_{\mathcal{T}}(\mathcal{C} \in \beta | \mathcal{R} = (\beta, s)) p^{S}_{\mathcal{T}}(s | \mathcal{R} \in \beta)\, ds \\
&= 1 - \frac{1}{\bar{t}(\beta)} \sum_{k=n(t^{\uparrow}(\beta))+1}^{n(t^{\downarrow}(\beta))} \int_{T_{k+1}}^{T_k} \left(\frac{1}{k} + e^{ks} \cdot \sum_{j=n(t^{\uparrow}(\beta))+1}^{k} Q_{kj}\right) ds \\
&= 1 - \frac{1}{\bar{t}(\beta)} \sum_{k=n(t^{\uparrow}(\beta))+1}^{n(t^{\downarrow}(\beta))} \left(\widetilde{Q}^1(k) + \widetilde{Q}^2(k, k, n(t^{\uparrow}(\beta)) + 1, 0, 1)\right),
\end{aligned}$$

where

$$\widetilde{Q}^1(k) := \frac{1}{k}\left(T_k - T_{k+1}\right),$$

and for $x, y, A, B \in \mathbb{Z}$, $x \geq k$, $2 \leq y \leq x$,

$$\widetilde{Q}^2(k, x, y, A, B) := \frac{1}{k}\left(e^{kT_k} - e^{kT_{k+1}}\right) \sum_{j=y}^{x} (Aj + B) \cdot Q_{kj}.$$

S2.3 Proof of Proposition S1.3

We have

$$\mathbb{P}_{\mathcal{T}}(\mathcal{C} \in \beta | \mathcal{C} = (\cdot, u), \mathcal{R} \notin \mathcal{B}(\beta), \mathcal{R} = (\cdot, s)) = \begin{cases} \frac{1}{n(u)} & \max(s, t^{\downarrow}(\beta)) < u < t^{\uparrow}(\beta) \\ 0 & \text{otherwise.} \end{cases}$$

For $s < t^{\downarrow}(\beta)$,

$$\begin{aligned}
\int_{t^{\downarrow}(\beta)}^{t^{\uparrow}(\beta)} \exp\left(-\int_s^u n(t)dt\right) du &= \sum_{j=n(t^{\uparrow}(\beta))+1}^{n(t^{\downarrow}(\beta))} \int_{T_{j+1}}^{T_j} \exp\left(-\int_s^u n(t)dt\right) du \\
&= \sum_{j=n(t^{\uparrow}(\beta))+1}^{n(t^{\downarrow}(\beta))} \exp\left(-\int_s^{T_{j+1}} n(t)\, dt\right) \int_{T_{j+1}}^{T_j} \exp\left(-\int_{T_{j+1}}^{u} j\, dt\right) du \\
&= e^{ks} \cdot \sum_{j=n(t^{\uparrow}(\beta))+1}^{n(t^{\downarrow}(\beta))} Q_{kj},
\end{aligned}$$

and the case $s \geq t^{\downarrow}(\beta)$ is given by (S2). Thus,

$$\mathbb{P}_{\mathcal{T}}(\mathcal{C} \in \beta | \mathcal{R} \notin \mathcal{B}(\beta), \mathcal{R} = (\cdot, s)) = \int_{\max(s, t^{\downarrow}(\beta))}^{t^{\uparrow}(\beta)} \exp\left(-\int_s^u n(t)dt\right) du$$

$$= \begin{cases} e^{ks} \sum_{j=n(t^{\uparrow}(\beta))+1}^{n(t^{\downarrow}(\beta))} Q_{kj} & s < t^{\downarrow}(\beta) \\ \frac{1}{k} + e^{ks} \sum_{j=n(t^{\uparrow}(\beta))+1}^{k} Q_{kj} & t^{\downarrow}(\beta) \le s < t^{\uparrow}(\beta) \\ 0 & \text{otherwise.} \end{cases}$$

S2.4 Proof of Proposition S1.4

Consider all of the possible orderings of the event times $t_1, \ldots, t_4$, as illustrated in Figure S11.

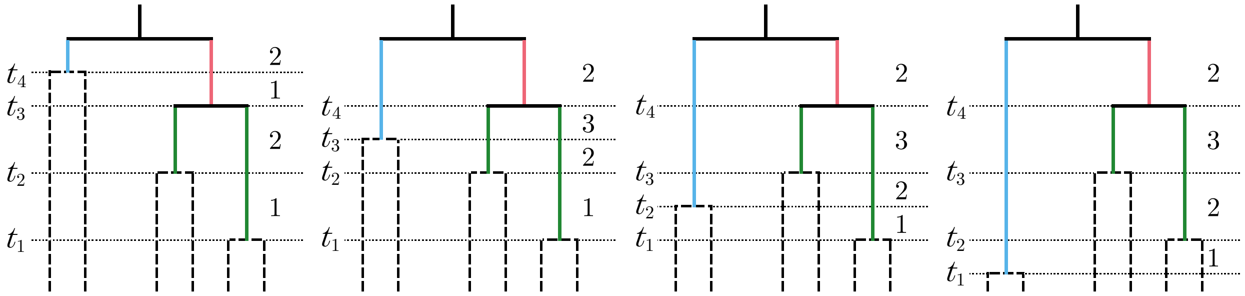

Figure S11: All possible orderings of $t^{\downarrow}(\mathrm{ch}_1(\beta)), t^{\downarrow}(\mathrm{ch}_2(\beta)), t^{\downarrow}(\mathrm{sib}(\beta)), t^{\downarrow}(\beta)$. The edge β is shown in red, $\mathrm{sib}(\beta)$ in blue, $\mathrm{ch}_1(\beta)$ and $\mathrm{ch}_2(\beta)$ in green. Numbers to the right of each tree show the number of lineages in $\mathcal{B}(\beta)$ in each time interval. For instance, in the leftmost tree, $t_1 = t^{\downarrow}(\mathrm{ch}_2(\beta))$, $t_2 = t^{\downarrow}(\mathrm{ch}_1(\beta))$, $t_3 = t^{\downarrow}(\beta)$ and $t_4 = t^{\downarrow}(\mathrm{sib}(\beta))$.

The number of lineages in set $\mathcal{B}(\beta)$ at time s can be written as

$$n_{\mathcal{B}(\beta)}(s) := \sum_{b' \in \mathcal{B}(\beta)} \mathbb{1}(s \in [t^{\downarrow}(b'), t^{\uparrow}(b')])$$

$$= \begin{cases} 1 & t_1 \le s < t_2 \\ 2 & t_2 \le s < t_3 \\ 1 + 2 \cdot \mathbb{1}(t^{\downarrow}(\mathrm{sib}(\beta)) < t^{\downarrow}(\beta)) & t_3 \le s < t_4 \\ 2 & t_4 \le s < t_5 = t^{\uparrow}(\beta) \\ 0 & \text{otherwise,} \end{cases}$$

where $\mathbb{1}(\cdot)$ is the indicator function. Then conditional on the recombination point not being on an edge in the set $\mathcal{B}(\beta)$, the density of the recombination event time is

$$p_{\mathcal{T}}^{S}(s|\mathcal{R} \notin \mathcal{B}(\beta)) = \begin{cases} \frac{n(s) - n_{\mathcal{B}(\beta)}(s)}{L_{\mathcal{T}}(0) - \sum_{b' \in \mathcal{B}(\beta)} \bar{t}(b')} & 0 \le s \le T_2 \\ 0 & \text{otherwise.} \end{cases}$$

Marginalising out the recombination time in (S10),

$$\mathbb{P}_{\mathcal{T}}(\mathcal{C} \in \beta | \mathcal{R} \notin \mathcal{B}(\beta)) = \int_0^{t^{\uparrow}(\beta)} \mathbb{P}_{\mathcal{T}}(\mathcal{C} \in \beta | \mathcal{R} \notin \mathcal{B}(\beta), \mathcal{R} = (\cdot, s)) \cdot p_{\mathcal{T}}^{S}(s | \mathcal{R} \notin \mathcal{B}(\beta))\, ds.$$

$$= \frac{1}{L_{\mathcal{T}}(0) - \sum_{b' \in \mathcal{B}(\beta)} \bar{t}(b')} \Bigg\{ \sum_{k=n(t_1)+1}^{n} k \widetilde{Q}^2(k, n(t^{\downarrow}(\beta)), n(t^{\uparrow}(\beta)) + 1, 0, 1)$$

$$+ \sum_{k=n(t_2)+1}^{n(t_1)} (k-1) \widetilde{Q}^2(k, n(t^{\downarrow}(\beta)), n(t^{\uparrow}(\beta)) + 1, 0, 1)$$

$$
\begin{aligned}
&+ \sum_{k=n(t_3)+1}^{n(t_2)} (k-2)\widetilde{Q}^2(k, n(t^\downarrow(\beta)), n(t^\uparrow(\beta)) + 1, 0, 1) \\
&+ \sum_{k=n(t_4)+1}^{n(t_3)} \Big[\mathbb{1}(t^\downarrow(\text{sib}(\beta)) < t^\downarrow(\beta))(k-3)\widetilde{Q}^2(k, n(t^\downarrow(\beta)), n(t^\uparrow(\beta)) + 1, 0, 1) \\
&\quad + \mathbb{1}(t^\downarrow(\text{sib}(\beta)) \geq t^\downarrow(\beta))(k-1)\left(\widetilde{Q}^1(k) + \widetilde{Q}^2(k, k, n(t^\uparrow(\beta)) + 1, 0, 1)\right)\Big] \\
&+ \sum_{k=n(t_5)+1}^{n(t_4)} (k-2)\left(\widetilde{Q}^1(k) + \widetilde{Q}^2(k, k, n(t^\uparrow(\beta)) + 1, 0, 1)\right) \Bigg\}.
\end{aligned}
$$

S2.5 Proof of Theorem S1.1

If we now say that an edge is disrupted only if there is a change in topology (but not edge length), we can allow the events shown in Figure 2 in blue (marked with dots), i.e. those where the recombination point is on edge $\beta \in \mathcal{B}(\beta)$, and the coalescence point is on one of the edges in $\mathcal{A}(\beta)$. Thus,

$$
\begin{aligned}
\mathbb{P}_\mathcal{T}(b \text{ topologically disrupted}) = &\sum_{b' \in \mathcal{B}(b)} \mathbb{P}_\mathcal{T}(\mathcal{C} \notin \mathcal{A}(b') | \mathcal{R} \in b') \cdot \mathbb{P}_\mathcal{T}(\mathcal{R} \in b') \\
&+ \mathbb{P}_\mathcal{T}(\mathcal{C} \in b | \mathcal{R} \notin \mathcal{B}(b)) \cdot \mathbb{P}_\mathcal{T}(\mathcal{R} \notin \mathcal{B}(b)).
\end{aligned}
$$

To calculate the probability $\mathbb{P}_\mathcal{T}(\mathcal{C} \notin \mathcal{A}(\beta) | \mathcal{R} \in \beta)$, we follow the same approach as the proofs of Propositions S1.1 and S1.2, first conditioning on the recombination point $\mathcal{R} = (\beta, s)$. Let $k = n(s)$, so that T_k is the first coalescence time just above time s. Let

$$
r(u) := \exp\left(-\int_s^u n(t)dt\right).
$$

Then if $t^\downarrow(\text{sib}(b)) < s$,

$$
\begin{aligned}
\mathbb{P}_\mathcal{T}(\mathcal{C} \in \mathcal{A}(\beta) | \mathcal{R} = (\beta, s)) &= \int_0^\infty \mathbb{P}(\mathcal{C} \in \mathcal{A}(\beta) | \mathcal{C} = (\cdot, u), \mathcal{R} = (\beta, s)) \cdot p^U_\mathcal{T}(u | \mathcal{R} = (\cdot, s)) du \\
&= \int_s^{t^\uparrow(\beta)} 2 \cdot r(u)\, du + \int_{t^\uparrow(\beta)}^{t^\uparrow(\text{par}(\beta))} r(u)\, du \\
&= \frac{2}{k} + e^{ks} \cdot \left(\sum_{j=n(t^\uparrow(\beta))+1}^{k} 2 \cdot Q_{kj} + \sum_{j=n(t^\uparrow(\text{par}(\beta)))+1}^{n(t^\uparrow(\beta))} Q_{kj} \right),
\end{aligned}
$$

and if $t^\downarrow(\text{sib}(b)) \geq s$,

$$
\begin{aligned}
\mathbb{P}_\mathcal{T}(\mathcal{C} \in \mathcal{A}(\beta) | \mathcal{R} = (\beta, s)) &= \int_s^{t^\downarrow(\text{sib}(\beta))} r(u)\, du + \int_{t^\downarrow(\text{sib}(\beta))}^{t^\uparrow(\beta)} 2 \cdot r(u)\, du + \int_{t^\uparrow(\beta)}^{t^\uparrow(\text{par}(\beta))} r(u)\, du \\
&= \frac{1}{k} + e^{ks} \cdot \left(\sum_{j=n(t^\downarrow(\text{sib}(\beta)))+1}^{k} Q_{kj} + \sum_{j=n(t^\uparrow(\beta))+1}^{n(t^\downarrow(\text{sib}(\beta)))} 2 \cdot Q_{kj} + \sum_{j=n(t^\uparrow(\text{par}(\beta)))+1}^{n(t^\uparrow(\beta))} Q_{kj} \right).
\end{aligned}
$$

Marginalising out the recombination time,

$$
\begin{aligned}
\mathbb{P}_\mathcal{T}(\mathcal{C} \notin \mathcal{A}(\beta) | \mathcal{R} \in \beta) &= 1 - \int_{t^\downarrow(\beta)}^{t^\uparrow(\beta)} \mathbb{P}_\mathcal{T}(\mathcal{C} \in \mathcal{A}(\beta) | \mathcal{R} = (\beta, s)) p^S_\mathcal{T}(s | \mathcal{R} \in \beta)\, ds \\
&= 1 - \frac{1}{\bar{t}(\beta)} \sum_{k=n(t^\uparrow(\beta))+1}^{n(t^\downarrow(\beta))} G_\beta(k),
\end{aligned}
$$

where for $k \le n(t^{\downarrow}(\text{sib}(\beta)))$,

$$G_\beta(k) \;=\; 2\cdot \widetilde{Q}^1(k) \,+\, 2\cdot \widetilde{Q}^2(k,k,n(t^{\uparrow}(\beta)) \,+\, 1,0,1) + \widetilde{Q}^2(k, n(t^{\uparrow}(\beta)), n(t^{\uparrow}(\text{par}(\beta))) \,+\, 1,0,1),$$

and for $k > n(t^{\downarrow}(\text{sib}(\beta)))$

$$\begin{aligned} G_\beta(k) = \widetilde{Q}^1(k) + \widetilde{Q}^2(k,k,n(t^{\downarrow}(\text{sib}(\beta))) + 1,0,1) + 2\cdot \widetilde{Q}^2(k, n(t^{\downarrow}(\text{sib}(\beta))), n(t^{\uparrow}(\beta)) + 1,0,1) \\ + \widetilde{Q}^2(k, n(t^{\uparrow}(\beta)), n(t^{\uparrow}(\text{par}(\beta))) + 1,0,1). \end{aligned}$$

S2.6 Proof of Proposition S1.5

S2.6.1 Probability of no change in total branch length

The probability that the recombination event does not result in a change in total branch length is

$$\begin{aligned} \mathbb{P}_{\mathcal{T}}(C=0) &= \sum_{b\in\mathcal{T}} \frac{\bar{t}(b)}{L_{\mathcal{T}}(0)} \left(1 - P_{\mathcal{T}}(\mathcal{C} \notin b | \mathcal{R} \in b)\right) \\ &= \frac{1}{L_{\mathcal{T}}(0)} \sum_{b\in\mathcal{T}} \sum_{k=n(t^{\uparrow}(\beta))+1}^{n(t^{\downarrow}(\beta))} \left(\widetilde{Q}^1(k) + \widetilde{Q}^2(k,k,n(t^{\uparrow}(\beta)) + 1,0,1)\right), \end{aligned}$$

using (S7). This is equal to the probability derived by Deng et al. (2021, Theorem 1).

S2.6.2 Probability that change in total branch length is negative

We have

$$\begin{aligned} \mathbb{P}_{\mathcal{T}}(C<0|\mathcal{R}=(b,s)) &= \int_s^{t^{\uparrow}(b)} \frac{n(u)-1}{n(u)} \cdot n(u) \exp\left(-\int_s^u n(t)\,dt\right) du \\ &= \frac{k-1}{k} + e^{ks} \sum_{j=n(t^{\uparrow}(b))+1}^{k} (j-1) Q_{kj}. \end{aligned}$$

Integrating over the recombination time,

$$\begin{aligned} \mathbb{P}_{\mathcal{T}}(C<0|\mathcal{R}\in b) &= \frac{1}{\bar{t}(b)} \int_{t^{\downarrow}(b)}^{t^{\uparrow}(b)} \left(\frac{k-1}{k} + e^{ks} \sum_{j=n(t^{\uparrow}(b))+1}^{k} (j-1) Q_{kj}\right) ds \\ &= \frac{1}{\bar{t}(b)} \sum_{k=n(t^{\uparrow}(b))+1}^{n(t^{\downarrow}(b))} \left((k-1)\widetilde{Q}^1(k) + \widetilde{Q}^2(k,k,n(t^{\uparrow}(\beta)) + 1,1,-1)\right). \end{aligned}$$

Then

$$\mathbb{P}_{\mathcal{T}}(C<0) = \frac{1}{L_{\mathcal{T}}(0)} \sum_{b\in\mathcal{T}} \sum_{k=n(t^{\uparrow}(b))+1}^{n(t^{\downarrow}(b))} \left((k-1)\widetilde{Q}^1(k) + \widetilde{Q}^2(k,k,n(t^{\uparrow}(\beta)) + 1,1,-1)\right).$$

S2.6.3 Probability that change in total branch length is positive

Similarly, the probability that the change in total branch length is positive is given by

$$\begin{aligned} \mathbb{P}_{\mathcal{T}}(C>0|\mathcal{R}=(b,s)) &= \int_{t^{\uparrow}(b)}^{\infty} n(u) \exp\left(-\int_s^u n(t)\,dt\right) du \\ &= \int_{t^{\uparrow}(b)}^{T_2} n(u) \exp\left(-\int_s^u n(t)\,dt\right) du + \int_{T_2}^{\infty} \exp\left(-\int_s^u n(t)\,dt\right) du \end{aligned}$$

$$= e^{ks} \sum_{j=2}^{n(t^\uparrow(b))} jQ_{kj} + e^{-L_\mathcal{T}(s)},$$

giving

$$\mathbb{P}_\mathcal{T}(C > 0|\mathcal{R} \in b) = \frac{1}{\bar{t}(b)} \sum_{k=n(t^\uparrow(b))+1}^{n(t^\downarrow(b))} \left(\widetilde{Q}^2(k, n(t^\uparrow(b)), 2, 1, 0) + \widetilde{Q}^3(k) \right),$$

where

$$\begin{aligned}
\widetilde{Q}^3(k) &= \int_{T_{k+1}}^{T_k} e^{-L_\mathcal{T}(s)}\, ds \\
&= \int_{T_{k+1}}^{T_k} e^{ks} \exp\left(-kT_k - L_\mathcal{T}(T_k)\right)\, ds \\
&= \frac{1}{k}\left(e^{kT_k} - e^{kT_{k+1}}\right) \exp\left(-kT_k - L_\mathcal{T}(T_k)\right) \\
&= \frac{1}{k}\left(e^{-L_\mathcal{T}(T_k)} - e^{-L_\mathcal{T}(T_{k+1})}\right).
\end{aligned}$$

Thus,

$$\mathbb{P}_\mathcal{T}(C > 0) = \frac{1}{L_\mathcal{T}(0)} \sum_{b\in\mathcal{T}} \sum_{k=n(t^\uparrow(b))+1}^{n(t^\downarrow(b))} \left(\widetilde{Q}^2(k, n(t^\uparrow(b)), 2, 1, 0) + \widetilde{Q}^3(k) \right).$$

S2.7 Proof of Proposition S1.6

We first calculate the density of change in total branch length conditional on the recombination point $\mathcal{R} = (b, s)$, then marginalise out the recombination time and edge.

S2.8 Density of change in total branch length conditional on recombination point

Suppose that the recombination point is on edge b at time s. Then given the coalescence time u, the change in total branch length $C > t^\downarrow(b) + T_3 - 2T_2$ given $C \neq 0$ is

$$C = \begin{cases} u + T_3 - 2T_2 & t^\uparrow(b) = T_2 \text{ and } u \le T_3 \\ 2u - t^\uparrow(b) - T_2 & t^\uparrow(b) = T_2 \text{ and } u > T_3, \text{ or } u > T_2 \\ u - t^\uparrow(b) & \text{otherwise.} \end{cases}$$

Let $l = n(u)$, so T_l is the time of the first coalescence event just above time u. We need to condition on the coalescence point not being on edge b, and as a simplification we take

$$p_\mathcal{T}^U(u|\mathcal{R} = (\cdot, s), \mathcal{C} \notin b) = \begin{cases} n(u) \exp\left(-\int_s^u n(t)dt\right) & u \ge t^\uparrow(b) \\ (n(u) - 1) \exp\left(-\int_s^u (n(t) - 1)dt\right) & s \le u < t^\uparrow(b) \\ 0 & \text{otherwise.} \end{cases}$$

This essentially assumes SMC rather than SMC' dynamics, since the two models differ only in that the latter allows the coalescence event to occur on the same branch as the recombination event, so this is a very close match for the conditional distribution (and simplifies our calculations). Thus, through a change of variable in (S1), for $t^\uparrow(b) \neq T_2$ and $s - t^\uparrow(b) < c < 0$,

$$\begin{aligned}
p_\mathcal{T}^C(c|C \neq 0, \mathcal{R} = (b, s), t^\uparrow(b) \neq T_2) &= (n(u) - 1) \exp\left(-\int_s^u (n(t) - 1)\, dt\right) \\
&= (n(u) - 1) \exp\left(\left[-\int_s^{T_k} - \int_{T_k}^{T_l} + \int_u^{T_l}\right] (n(t) - 1)\, dt\right)
\end{aligned}$$

$$= e^{(k-1)s} P^1_{kl} (n(u) - 1) e^{-(l-1)u}$$
$$= e^{(k-1)s} P^1_{kl} (n(c + t^{\uparrow}(b)) - 1) e^{-(l-1)(c+t^{\uparrow}(b))},$$

where

$$P^1_{kl} := \exp\left(-(k-1)T_k - \sum_{i=l}^{k-1}(i-1)(T_i - T_{i+1}) + (l-1)T_l\right)$$
$$= \exp\left(l \cdot T_l - k \cdot T_k + L_{\mathcal{T}}(T_l) - L_{\mathcal{T}}(T_k)\right).$$

For $t^{\uparrow}(b) = T_2$ and $t^{\downarrow}(b) + T_3 - 2T_2 \leq c \leq 2(T_3 - T_2)$, similarly,

$$p^C_{\mathcal{T}}(c | C \neq 0, \mathcal{R} = (b, s), t^{\uparrow}(b) = T_2) = e^{(k-1)s} P^1_{kl}(n(c + 2T_2 - T_3) - 1)e^{-(l-1)(c+2T_2-T_3)},$$

and for $t^{\uparrow}(b) = T_2$ and $2(T_3 - T_2) < c < 0$, since $l = 2$,

$$p^C_{\mathcal{T}}(c | C \neq 0, \mathcal{R} = (b, s), t^{\uparrow}(b) = T_2) = \frac{1}{2} e^{(k-1)s} P^1_{kl}\left(n\left(c/2 + T_2\right) - 1\right) e^{-(c/2+T_2)}.$$

For $0 < c \leq T_2 - t^{\uparrow}(b)$,

$$p^C_{\mathcal{T}}(c | C \neq 0, \mathcal{R} = (b, s)) = n(u) \exp\left(-\int_s^{t^{\uparrow}(b)} (n(t) - 1)\, dt - \int_{t^{\uparrow}(b)}^{u} n(t)\, dt\right)$$
$$= \exp\left(-\int_s^{t^{\uparrow}(b)} (n(t) - 1)\, dt\right) n(u) \exp\left(-\int_{t^{\uparrow}(b)}^{u} n(t)\, dt\right)$$
$$= e^{(k-1)s} P^2_k n(c + t^{\uparrow}(b)) \exp\left(-\int_{t^{\uparrow}(b)}^{c+t^{\uparrow}(b)} n(t)\, dt\right)$$
$$= e^{(k-1)s} P^2_k n(c + t^{\uparrow}(b)) \exp\left(-\left[L_{\mathcal{T}}(t^{\uparrow}(b)) - L_{\mathcal{T}}(c + t^{\uparrow}(b))\right]\right),$$

where

$$P^2_k := \exp\left(-(k-1)T_k - \sum_{i=n(t^{\uparrow}(b))+1}^{k-1} (i-1)(T_i - T_{i+1})\right)$$
$$= \exp\left(t^{\uparrow}(b) - k \cdot T_k - L_{\mathcal{T}}(T_k) + L_{\mathcal{T}}(t^{\uparrow}(b))\right).$$

Finally, for $c > T_2 - t^{\uparrow}(b)$,

$$p^C_{\mathcal{T}}(c | C \neq 0, \mathcal{R} = (b, s)) = \exp\left(-\int_s^{t^{\uparrow}(b)} (n(t) - 1)\, dt - \int_{t^{\uparrow}(b)}^{T_2} n(t)\, dt - \int_{T_2}^{u} 1\, dt\right)$$
$$= \exp\left(-\int_s^{T_2} n(t)\, dt + \int_s^{t^{\uparrow}(b)} 1\, dt - \int_{T_2}^{u} 1\, dt\right)$$
$$= e^{(k-1)s} P^3_k e^{-(u-T_2)}$$
$$= \frac{1}{2} e^{(k-1)s} P^3_k e^{-(c+t^{\uparrow}(b)-T_2)/2},$$

where

$$P^3_k := \exp\left(-(k-1)T_k - \sum_{i=2}^{k-1} i(T_i - T_{i+1}) + \sum_{i=n(t^{\uparrow}(b))+1}^{k-1} (T_i - T_{i+1})\right)$$
$$= \exp\left(t^{\uparrow}(b) - k \cdot T_k - L_{\mathcal{T}}(T_k)\right).$$

Note that

$$e^{(k-1)s} P_k^3 = \exp\left(-\left[L_{\mathcal{T}}(s) - (t^{\uparrow}(b) - s)\right]\right)$$

gives the probability that the coalescence event happens above T_2. The conditional density $p_{\mathcal{T}}^C(c|C \neq 0, \mathcal{R} = (b,s))$ is thus

$$\begin{cases} e^{(k-1)s} P_{kl}^1 (n(c + t^{\uparrow}(b)) - 1) e^{-(l-1)(c+t^{\uparrow}(b))} & t^{\uparrow}(b) \neq T_2,\ s - t^{\uparrow}(b) \leq c < 0 \\ e^{(k-1)s} P_{kl}^1 (n(c + 2T_2 - T_3) - 1) e^{-(l-1)(c+2T_2-T_3)} & t^{\uparrow}(b) = T_2,\ t^{\downarrow}(b) + T_3 - 2T_2 \leq c < 2(T_3 - T_2) \\ \frac{1}{2} e^{(k-1)s} P_{kl}^1 \left(n\left(\frac{c}{2} + T_2\right) - 1\right) e^{-(c/2+T_2)} & t^{\uparrow}(b) = T_2,\ 2(T_3 - T_2) \leq c < 0 \\ e^{(k-1)s} P_k^2 n(c + t^{\uparrow}(b)) e^{-\left[L_{\mathcal{T}}(t^{\uparrow}(b)) - L_{\mathcal{T}}(c+t^{\uparrow}(b))\right]} & 0 < c \leq T_2 - t^{\uparrow}(b) \\ \frac{1}{2} e^{(k-1)s} P_k^3 e^{-(c+t^{\uparrow}(b)-T_2)/2} & c > T_2 - t^{\uparrow}(b) \\ 0 & \text{otherwise.} \end{cases}$$

S2.8.1 Density of change in total branch length

Marginalising out the position and time of the recombination point, we have

$$\begin{aligned} p_{\mathcal{T}}^C(c|C \neq 0) &= \sum_{b \in \mathcal{T}} \mathbb{P}(\mathcal{R} \in b) \int_0^{\infty} p_{\mathcal{T}}^S(s|\mathcal{R} \in b)\, p_{\mathcal{T}}^C(c|C \neq 0, \mathcal{R} = (b,s))\, ds \\ &= \frac{1}{L_{\mathcal{T}}(0)} \sum_{\substack{b \in \mathcal{T}: \\ c \geq -\bar{t}(b), \\ t^{\uparrow}(b) \neq T_2}} \int_{t^{\downarrow}(b)}^{t^{\uparrow}(b)+\min(0,c)} p_{\mathcal{T}}^C(c|C \neq 0, \mathcal{R} = (b,s))\, ds \\ &+ \frac{1}{L_{\mathcal{T}}(0)} \sum_{\substack{b \in \mathcal{T}: \\ t^{\downarrow}(b)+T_3-2T_2 \leq c < 2(T_3-T_2), \\ t^{\uparrow}(b) = T_2}} \int_{t^{\downarrow}(b)}^{c+2T_2-T_3} p_{\mathcal{T}}^C(c|C \neq 0, \mathcal{R} = (b,s))\, ds \\ &+ \frac{1}{L_{\mathcal{T}}(0)} \sum_{\substack{b \in \mathcal{T}: \\ 2(T_3-T_2) \leq c < 0, \\ t^{\uparrow}(b) = T_2}} \int_{t^{\downarrow}(b)}^{c/2+T_2} p_{\mathcal{T}}^C(c|C \neq 0, \mathcal{R} = (b,s))\, ds. \end{aligned}$$

Let

$$\begin{aligned} \widetilde{P}_{kl}^1 &:= \int_{T_{k+1}}^{T_k} e^{(k-1)s} P_{kl}^1\, ds = \frac{1}{k-1}\left(e^{(k-1)T_k} - e^{(k-1)T_{k+1}}\right) P_{kl}^1, \\ \widetilde{P}_k^2 &:= \int_{T_{k+1}}^{T_k} e^{(k-1)s} P_k^2\, ds = \frac{1}{k-1}\left(e^{(k-1)T_k} - e^{(k-1)T_{k+1}}\right) P_k^2, \\ \widetilde{P}_k^3 &:= \int_{T_{k+1}}^{T_k} e^{(k-1)s} P_k^3\, ds = \frac{1}{k-1}\left(e^{(k-1)T_k} - e^{(k-1)T_{k+1}}\right) P_k^3. \end{aligned}$$

Then for $t^{\uparrow}(b) \neq T_2$ and $-\bar{t} \leq c < 0$,

$$\begin{aligned} &\int_{t^{\downarrow}(b)}^{t^{\uparrow}(b)+c} e^{(k-1)s} P_{kl}^1 (n(c + t^{\uparrow}(b)) - 1) e^{-(l-1)(c+t^{\uparrow}(b))}\, ds \\ &\qquad = (n(c + t^{\uparrow}(b)) - 1) e^{-(l-1)(c+t^{\uparrow}(b))} \left(\sum_{k=l}^{n(t^{\downarrow}(b))} \int_{T_{k+1}}^{T_k} e^{(k-1)s} P_{kl}^1\, ds - \int_{c+t^{\uparrow}(b)}^{T_l} e^{(l-1)s}\, ds\right) \end{aligned}$$

$$= (n(c+t^{\uparrow}(b))-1)e^{-(l-1)(c+t^{\uparrow}(b))}\left(\sum_{k=l}^{n(t^{\downarrow}(b))}\widetilde{P}^1_{kl}-\frac{1}{l-1}\left(e^{(l-1)T_l}-e^{(l-1)(c+t^{\uparrow}(b))}\right)\right)$$

$$= (n(c+t^{\uparrow}(b))-1)\left(e^{-(l-1)(c+t^{\uparrow}(b))}\cdot\sum_{k=l}^{n(t^{\downarrow}(b))}\widetilde{P}^1_{kl}-\frac{1}{l-1}\left(e^{(l-1)(T_l-t^{\uparrow}(b)-c)}-1\right)\right).$$

Similarly, for $t^{\uparrow}(b)=T_2$ and $t^{\downarrow}(b)+T_3-2T_2\leq c<2(T_3-T_2)$,

$$\int_{t^{\downarrow}(b)}^{c+2T_2-T_3}e^{(k-1)s}P^1_{kl}(n(c+2T_2-T_3)-1)e^{-(l-1)(c+2T_2-T_3)}\,ds$$

$$= (n(c+2T_2-T_3)-1)\left(e^{-(l-1)(c+2T_2-T_3)}\cdot\sum_{k=l}^{n(t^{\downarrow}(b))}\widetilde{P}^1_{kl}-\frac{1}{l-1}\left(e^{(l-1)(T_l-c-2T_2+T_3)}-1\right)\right),$$

and for $t^{\uparrow}(b)=T_2$ and $2(T_3-T_2)\leq c<0$,

$$\int_{t^{\downarrow}(b)}^{c/2+T_2}\frac{1}{2}e^{(k-1)s}P^1_{kl}(n(c/2+T_2)-1)e^{-(l-1)(c/2+T_2)}\,ds$$

$$= \frac{1}{2}(n(c/2+T_2)-1)\left(e^{-(l-1)(c/2+T_2)}\cdot\sum_{k=l}^{n(t^{\downarrow}(b))}\widetilde{P}^1_{kl}-e^{T_l-c/2-T_2}+1\right).$$

For $0<c\leq T_2-t^{\uparrow}(b)$,

$$\int_{t^{\downarrow}(b)}^{t^{\uparrow}(b)}e^{(k-1)s}P^2_k n(c+t^{\uparrow}(b))e^{-\left[L_{\mathcal{T}}(t^{\uparrow}(b))-L_{\mathcal{T}}(c+t^{\uparrow}(b))\right]}\,ds$$

$$= n(c+t^{\uparrow}(b))e^{-\left[L_{\mathcal{T}}(t^{\uparrow}(b))-L_{\mathcal{T}}(c+t^{\uparrow}(b))\right]}\sum_{k=n(t^{\uparrow}(b))+1}^{n(t^{\downarrow}(b))}\int_{T_{k+1}}^{T_k}e^{(k-1)s}P^2_k\,ds$$

$$= n(c+t^{\uparrow}(b))e^{-\left[L_{\mathcal{T}}(t^{\uparrow}(b))-L_{\mathcal{T}}(c+t^{\uparrow}(b))\right]}\sum_{k=n(t^{\uparrow}(b))+1}^{n(t^{\downarrow}(b))}\widetilde{P}^2_k.$$

For $c>T_2-t^{\uparrow}(b)$,

$$\int_{t^{\downarrow}(b)}^{t^{\uparrow}(b)}\frac{1}{2}e^{(k-1)s}P^3_k e^{-(c+t^{\uparrow}(b)-T_2)/2}\,ds$$

$$= \frac{1}{2}e^{-(c+t^{\uparrow}(b)-T_2)/2}\sum_{k=n(t^{\uparrow}(b))+1}^{n(t^{\downarrow}(b))}\int_{T_{k+1}}^{T_k}e^{(k-1)s}P^3_k\,ds$$

$$= \frac{1}{2}e^{-(c+t^{\uparrow}(b)-T_2)/2}\sum_{k=n(t^{\uparrow}(b))+1}^{n(t^{\downarrow}(b))}\widetilde{P}^3_k.$$

Thus,

$$p^C_{\mathcal{T}}(c|C\neq 0)=\frac{1}{L_{\mathcal{T}}(0)}\sum_{b\in\mathcal{T}}\xi_b(c),$$

where $\xi_b(c)$ is given by

$$\begin{cases} (n(c+t^{\uparrow}(b))-1)\left(e^{-(l-1)(c+t^{\uparrow}(b))}\cdot\sum_{k=l}^{n(t^{\downarrow}(b))}\widetilde{P}^1_{kl}-\frac{e^{(l-1)(T_l-t^{\uparrow}(b)-c)}-1}{l-1}\right) & t^{\uparrow}(b)\neq T_2,\ -\bar{t}(b)\leq c<0 \\ (n(c+2T_2-T_3)-1)\left(e^{-(l-1)(c+2T_2-T_3)}\cdot\sum_{k=l}^{n(t^{\downarrow}(b))}\widetilde{P}^1_{kl}-\frac{e^{(l-1)(T_l-c-2T_2+T_3)}-1}{l-1}\right) & t^{\uparrow}(b)=T_2, \\ & t^{\downarrow}(b)+T_3-2T_2\leq c<2(T_3-T_2) \\ \frac{1}{2}(n(c/2+T_2)-1)\left(e^{-(c/2+T_2)}\cdot\sum_{k=l}^{n(t^{\downarrow}(b))}\widetilde{P}^1_{kl}-e^{T_l-c/2-T_2}+1\right) & t^{\uparrow}(b)=T_2,\ 2(T_3-T_2)\leq c<0 \\ n(c+t^{\uparrow}(b))e^{-\left[L_{\mathcal{T}}(t^{\uparrow}(b))-L_{\mathcal{T}}(c+t^{\uparrow}(b))\right]}\cdot\sum_{k=n(t^{\uparrow}(b))+1}^{n(t^{\downarrow}(b))}\widetilde{P}^2_k & 0<c\leq T_2-t^{\uparrow}(b) \\ \frac{1}{2}e^{-(c+t^{\uparrow}(b)-T_2)/2}\cdot\sum_{k=n(t^{\uparrow}(b))+1}^{n(t^{\downarrow}(b))}\widetilde{P}^3_k & c>T_2-t^{\uparrow}(b) \\ 0 & \text{otherwise.} \end{cases}$$

S2.9 Proof of Proposition S1.7

The probability of a negative change in tree height, conditional on the location and time of the recombination point, is

$$\mathbb{P}_{\mathcal{T}}(H<0|\mathcal{R}=(b,s))=\begin{cases}\frac{k-1}{k}-e^{ks}\left(\exp\left(-kT_k-L_{\mathcal{T}}(T_k)\right)+\sum_{j=2}^{k}Q_{kj}\right) & b\in\mathcal{M}\\ 0 & b\notin\mathcal{M}.\end{cases}$$

Integrating over the recombination time gives

$$\mathbb{P}_{\mathcal{T}}(H<0|\mathcal{R}\in b)=\begin{cases}\frac{1}{\bar{t}(b)}\sum_{k=2}^{n(t^{\downarrow}(b))}\left\{(k-1)\widetilde{Q}^1(k)-\widetilde{Q}^2(k,k,2,0,1)-\widetilde{Q}^3(k)\right\} & b\in\mathcal{M}\\ 0 & b\notin\mathcal{M}.\end{cases}$$

Summing over the edges and multiplying by the corresponding probability, the unconditional probability that the change in tree height is negative is thus

$$\mathbb{P}_{\mathcal{T}}(H<0)=\frac{1}{L_{\mathcal{T}}(0)}\sum_{b\in\mathcal{M}}\sum_{k=2}^{n(t^{\downarrow}(b))}\left\{(k-1)\widetilde{Q}^1(k)-\widetilde{Q}^2(k,k,2,0,1)-\widetilde{Q}^3(k)\right\}.$$

The probability of no change in tree height, conditional on the recombination point, is

$$\mathbb{P}_{\mathcal{T}}(H=0|\mathcal{R}=(b,s))=\begin{cases}\frac{1}{k}+e^{ks}\sum_{j=2}^{k}Q_{kj} & b\in\mathcal{M}\\ 1-e^{ks}\exp\left(-kT_k-L_{\mathcal{T}}(T_k)\right) & b\notin\mathcal{M},\end{cases}$$

and

$$\mathbb{P}_{\mathcal{T}}(H=0|\mathcal{R}\in b)=\begin{cases}\frac{1}{\bar{t}(b)}\sum_{k=2}^{n(t^{\downarrow}(b))}\left(\widetilde{Q}^1(k)+\widetilde{Q}^2(k,k,2,0,1)\right) & b\in\mathcal{M}\\ 1-\frac{1}{\bar{t}(b)}\sum_{k=n(t^{\uparrow}(b))+1}^{n(t^{\downarrow}(b))}\widetilde{Q}^3(k) & b\notin\mathcal{M}.\end{cases}$$

The probability of no change in tree height is thus

$$\mathbb{P}_{\mathcal{T}}(H=0)=\frac{1}{L_{\mathcal{T}}(0)}\left\{\sum_{b\in\mathcal{M}}\sum_{k=2}^{n(t^{\downarrow}(b))}\left(\widetilde{Q}^1(k)+\widetilde{Q}^2(k,k,2,0,1)\right)+\sum_{b\notin\mathcal{M}}\left(\bar{t}(b)-\sum_{k=n(t^{\uparrow}(b))+1}^{n(t^{\downarrow}(b))}\widetilde{Q}^3(k)\right)\right\}.$$

Finally, the probability of a positive change in height, conditional on the recombination point, is

$$\mathbb{P}_{\mathcal{T}}(H > 0|\mathcal{R} = (b, s)) = e^{ks} \exp\left(-kT_k - L_{\mathcal{T}}(T_k)\right),$$

and

$$\mathbb{P}_{\mathcal{T}}(H > 0|\mathcal{R} \in b) = \frac{1}{\bar{t}(b)} \sum_{k=n(t^{\uparrow}(b))+1}^{n(t^{\downarrow}(b))} \widetilde{Q}^3(k).$$

The probability that the change in tree height is positive is

$$\mathbb{P}_{\mathcal{T}}(H > 0) = \frac{1}{L_{\mathcal{T}}(0)} \sum_{b\in\mathcal{T}} \sum_{k=n(t^{\uparrow}(b))+1}^{n(t^{\downarrow}(b))} \widetilde{Q}^3(k).$$

S2.10 Proof of Theorem S1.2

Conditional on $\mathcal{T}$, the recombination point is chosen uniformly along the edges, so

$$\mathbb{P}(\mathcal{R} \in G) = \frac{L_G(0)}{L_{\mathcal{T}}(0)}, \quad \mathbb{P}(\mathcal{R} \notin G \cup g) = \frac{L_{\mathcal{T}} - L_G(0) - \bar{t}(g)}{L_{\mathcal{T}}(0)}.$$

Conditional on the recombination point being in G and letting the clade MRCA time be $t^{\downarrow}(g) = T_m$, the density of the recombination time is

$$p_S(s|\mathcal{R} \in G) = \begin{cases} \frac{n_G(s)}{L_G(0)} & \text{for } s \le T_m \\ 0 & \text{otherwise,} \end{cases}$$

and similarly

$$p_S(s|\mathcal{R} \notin G \cup g) = \begin{cases} \frac{n(s) - n_{G\cup g}(s)}{L_{\mathcal{T}}(0) - L_G(0) - \bar{t}(g)} & \text{for } s \le T_2 \\ 0 & \text{otherwise.} \end{cases}$$

First conditioning on the recombination time,

$$\begin{aligned} \mathbb{P}(\mathcal{C} \in G \cup g|\mathcal{R} = (G, s)) &= \int_s^{t^{\uparrow}(g)} \frac{n_{G\cup g}(u)}{n(u)} n(u) \exp\left(-\int_s^u n(t)dt\right) du \\ &= \int_s^{T_k} n_{G\cup g}(T_{k+1}) \exp\left(-\int_s^u kdt\right) du \\ &+ \sum_{j=n(t^{\uparrow}(g))+1}^{k-1} \int_{T_{j+1}}^{T_j} n_{G\cup g}(T_{j+1}) \exp\left(-\int_s^u n(t)dt\right) du \\ &= \frac{1}{k} n_{G\cup g}(T_{k+1}) + e^{ks} \sum_{j=n(t^{\uparrow}(g))+1}^{k} n_{G\cup g}(T_{j+1}) Q_{kj}, \end{aligned}$$

and so

$$\begin{aligned} \mathbb{P}(\mathcal{C} \in G \cup g|\mathcal{R} \in G) &= \frac{1}{L_G(0)} \sum_{k=n(t^{\downarrow}(g))+1}^{n} \int_{T_{k+1}}^{T_k} n_G(T_{k+1}) \mathcal{P}(\mathcal{C} \in G \cup g|\mathcal{R} \in G, \mathcal{R} = (\cdot, s)) ds \\ &= \frac{1}{L_G(0)} \sum_{k=n(t^{\downarrow}(g))+1}^{n} \left[n_{G\cup g}(T_{k+1}) \widetilde{Q}^1(k) + \widetilde{Q}^4(k, G \cup g)\right] n_G(T_{k+1}), \end{aligned}$$

with

$$\widetilde{Q}^4(k, A) = \frac{1}{k}\left(e^{kT_k} - e^{kT_{k+1}}\right) \sum_{j=n(t^{\uparrow}(A))+1}^{k} n_A(T_{j+1}) Q_{kj}.$$

Similarly,

$$\begin{aligned}\mathbb{P}(\mathcal{C} \in G | \mathcal{R} \notin G \cup g, \mathcal{R} = (\cdot, s)) &= \int_s^{t^{\downarrow}(g)} \frac{n_G(u)}{n(u)} n(u) r(u) du \\ &= \frac{1}{k} n_G(T_{k+1}) + \sum_{j=n(t^{\downarrow}(g))+1}^{k} e^{ks} n_G(T_{j+1}) Q_{kj},\end{aligned}$$

and

$$\begin{aligned}\mathbb{P}(\mathcal{C} \in G | \mathcal{R} \notin G \cup g) = &\frac{1}{L_{\mathcal{T}}(0) - L_G(0) - \bar{t}(g)} \sum_{k=n(t^{\downarrow}(g))+1}^{n} \int_{T_{k+1}}^{T_k} (k - n_{G \cup g}(T_{k+1})) \\ &\cdot \mathbb{P}(\mathcal{C} \in G | \mathcal{R} \notin G \cup g, \mathcal{R} = (\cdot, s)) ds \\ = &\frac{1}{L_{\mathcal{T}}(0) - L_G(0) - \bar{t}(g)} \sum_{k=n(t^{\downarrow}(g))+1}^{n} (k - n_{G \cup g}(T_{k+1})) \left[n_G(T_{k+1}) \widetilde{Q}^1(k) + \widetilde{Q}^4(k, G) \right].\end{aligned}$$

S3 Supplementary Figures

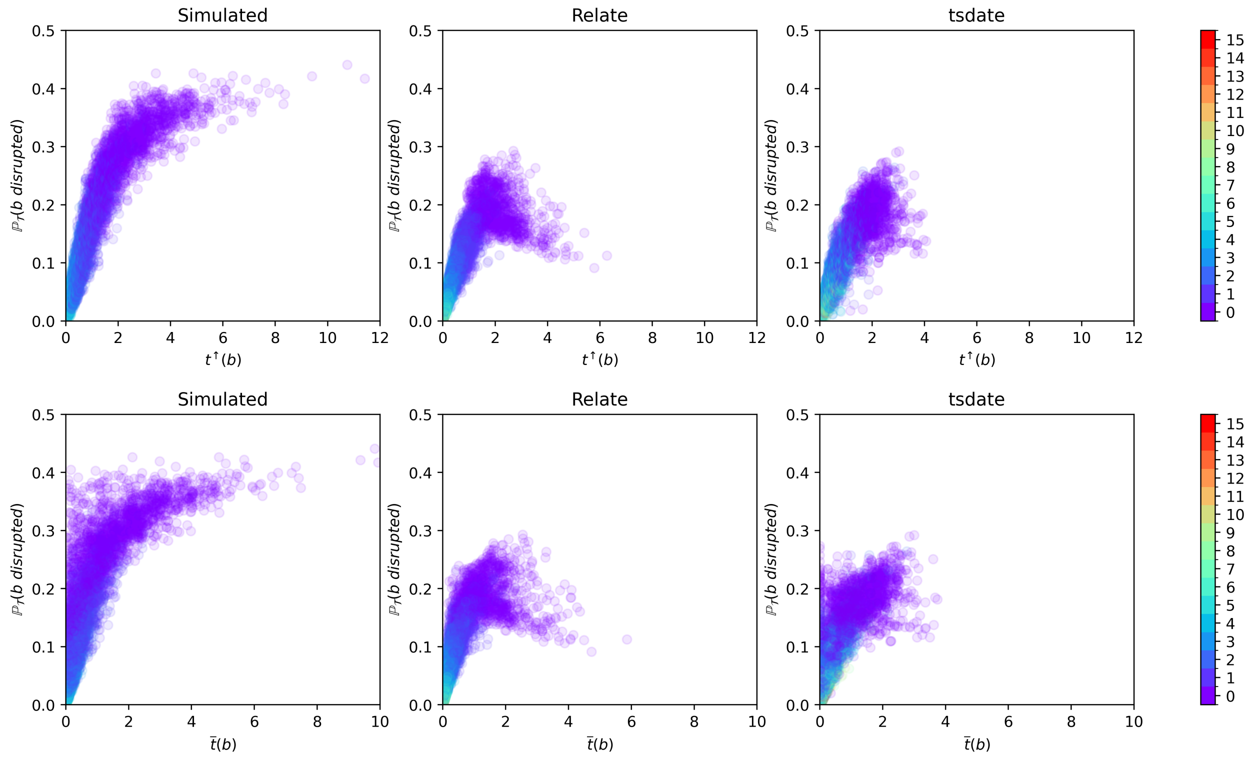

Figure S12: Same as Figure 2, but with x-axis showing the (un-normalised) time at the upper endpoint of each edge $t^{\uparrow}(b)$ (top panel), and time-length of each edge $\bar{t}(b)$ (bottom panel).

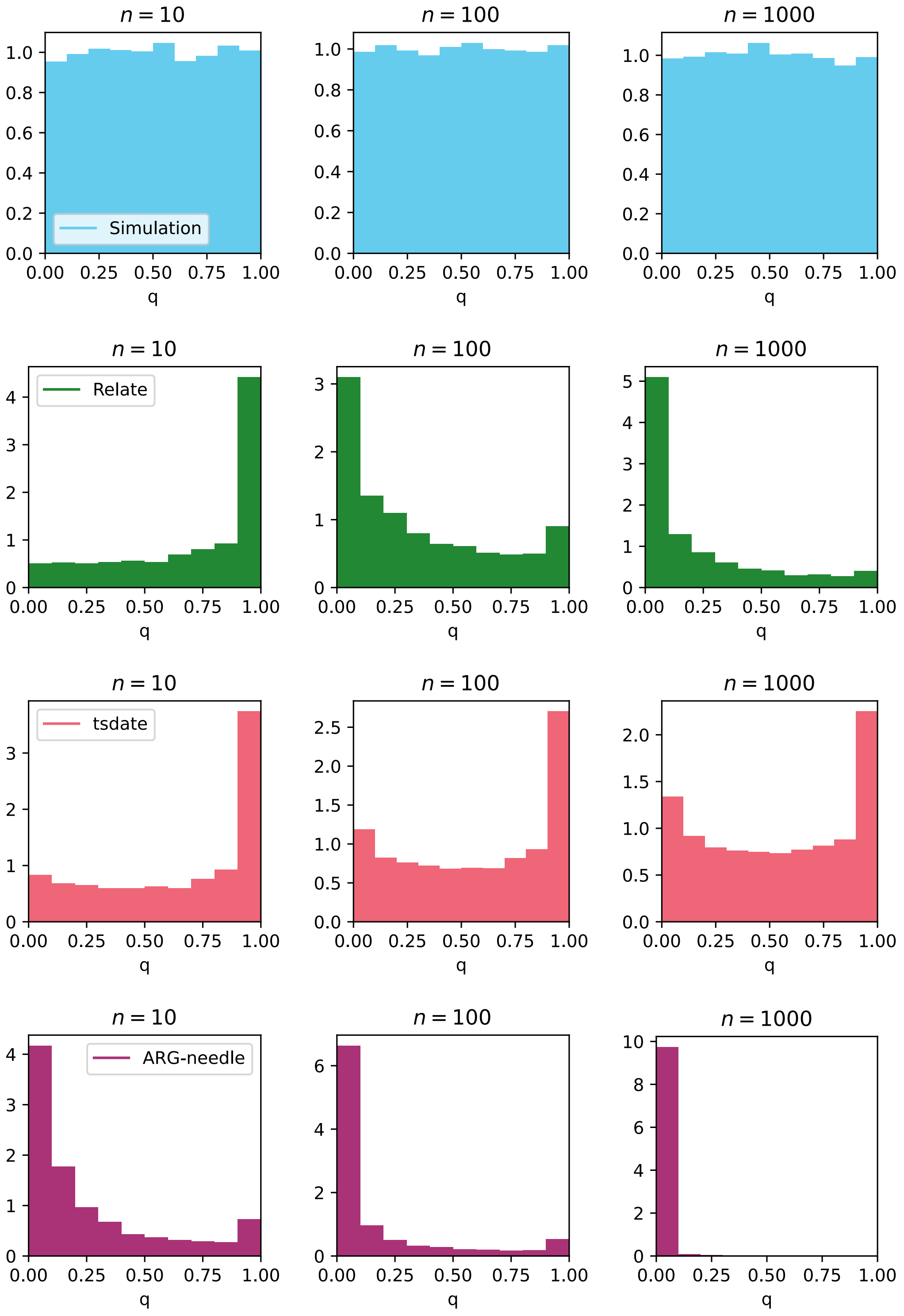

Figure S13: Histograms corresponding to Q-Q plots in Figure 3.

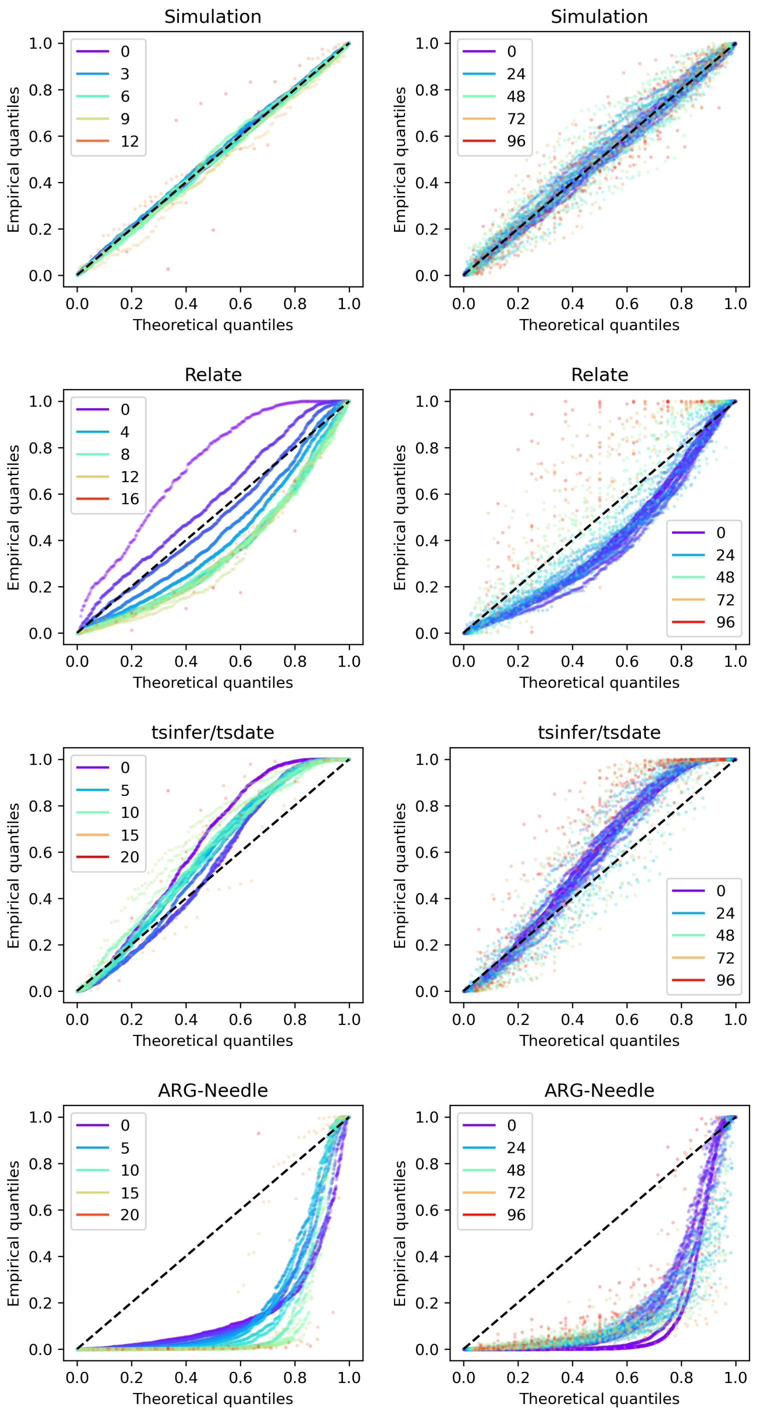

Figure S14: Q-Q plots split by clade size and depth. Q-Q plots using (S23) computed from an ARG simulated using dataset 1 parameters under the SMC' (with $n = 100$). Left panels: split by depth of the edge (defined as the number of edges to the root of the tree); right panels: split by clade size (defined as the number of samples subtended by the edge). Dashed line: diagonal from $(0, 0)$ to $(1, 1)$. For the simulated ARGs, none of the corresponding K–S p-values for each group are significant using a 0.05 significance threshold.

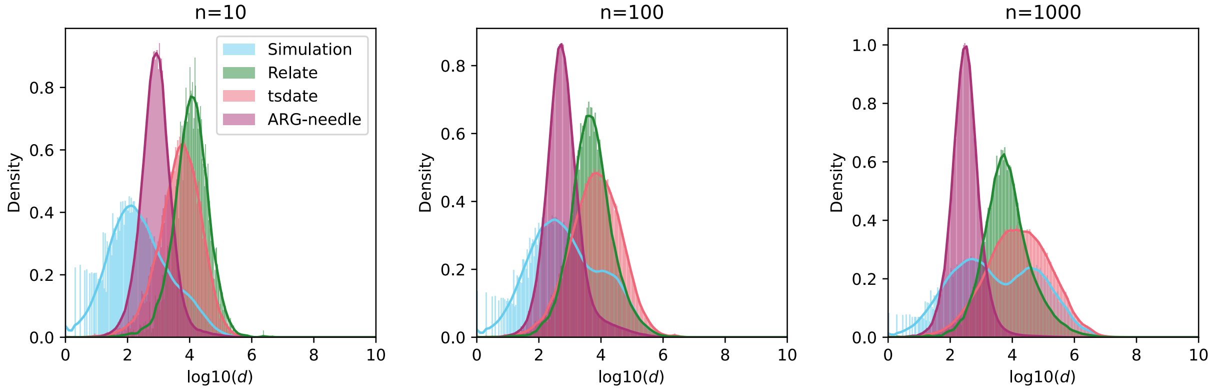

Figure S15: Histograms of observed edge span for simulated and reconstructed ARGs. Histograms of (observed) edge span, calculated as $d(b) = d^{\rightarrow}(b) - d^{\leftarrow}(b)$ for each edge b in simulated and reconstructed ARGs (same as those in Figure 3). Note log scale on the x-axis.

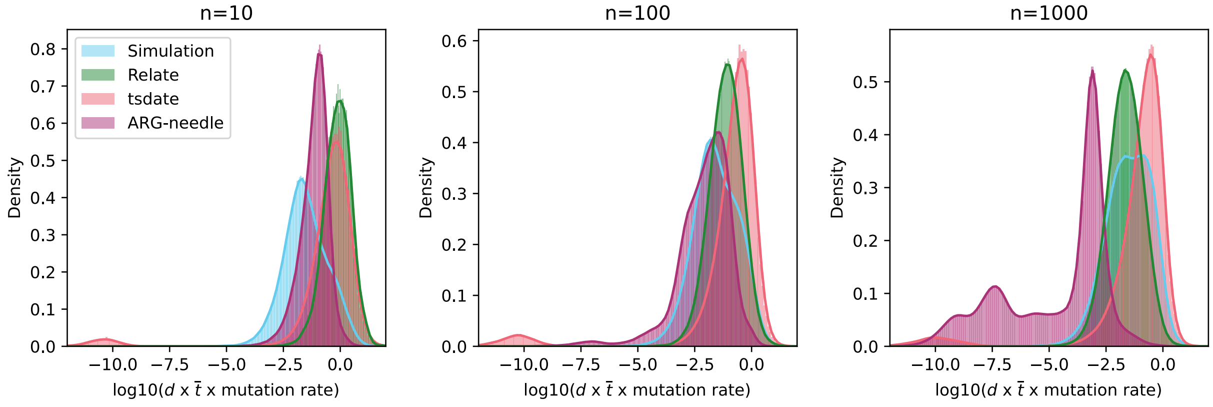

Figure S16: Histograms of expected number of mutations per edge for simulated and reconstructed ARGs. Histograms of (observed) expected number of mutations per edge, calculated as $(d^{\rightarrow}(b) - d^{\leftarrow}(b)) \cdot \bar{t}(b) \cdot \mu$ for each edge b in simulated and reconstructed ARGs (same as those in Figure 3). Note log scale on the x-axis.

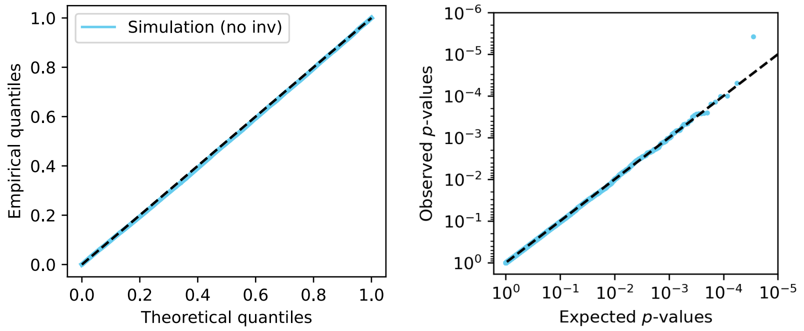

Figure S17: Q-Q and p-value plots (simulated ARG without inversion). Q-Q plot (left panel) and p-value plot (right panel) for ARG simulated using SLiM (without inversions and otherwise same parameters as in Section 4.6.2, main text). No clades have p-values below the Bonferroni-corrected significance threshold.

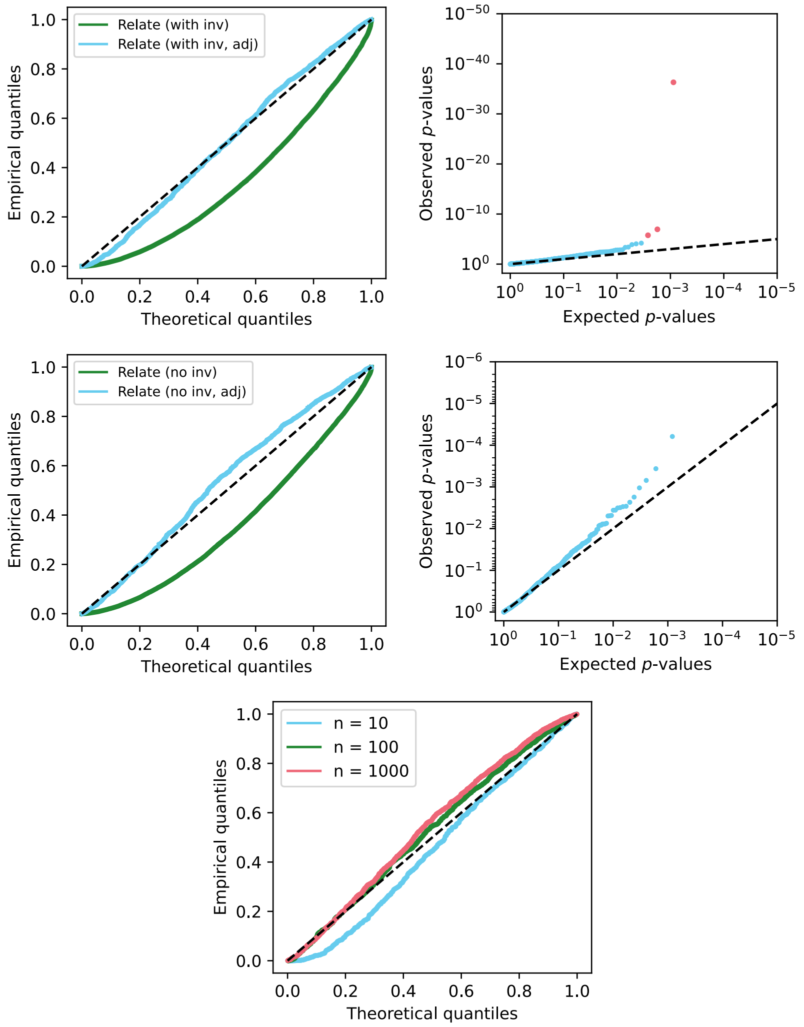

Figure S18: Q-Q and p-value plots (ARGs reconstructed using Relate). Top row: Q-Q plot (left panel) and p-value plot (right panel) for ARG reconstructed using Relate from data simulated using SLiM with one inversion under balancing selection (as described in Section 2.4.1). Blue (resp. green) points show values calculated after (resp. before) applying the adjustments described in Section S1.13.1; red points correspond to clades with p-value below the Bonferroni-corrected significance threshold. Middle and bottom rows: same using SLiM simulation without inversions (and otherwise same parameters); bottom panel shows QQ plot for Relate trees after the adjustments are applied, with varying sample sizes.

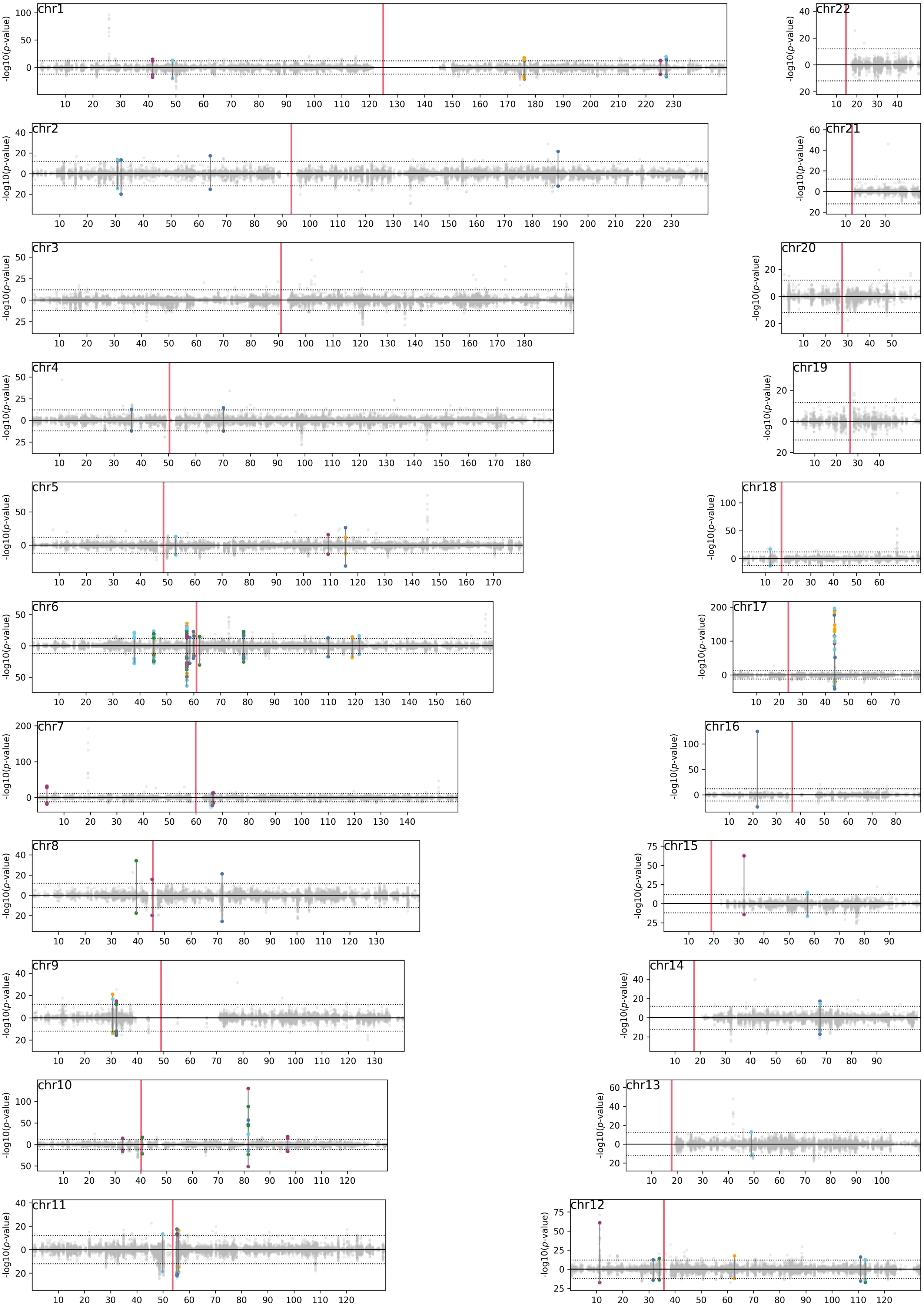

Figure S19: DoLoReS p-values for 1KGP ARG. Red vertical lines indicate positions of centromeres. See caption of Figure 6 (main text).

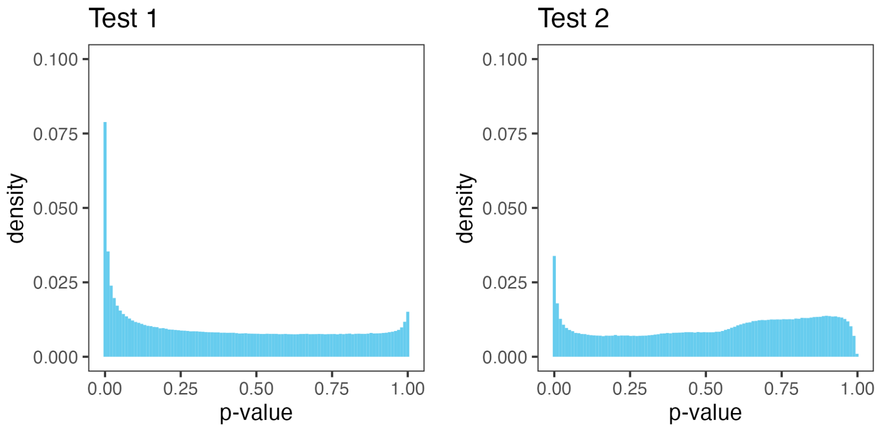

Figure S20: Histograms of p-values for Tests 1 and 2 for the 1KGP ARG (all populations combined, all clades with more than 2 mutations, at least 10 and at most $n-10$ samples, spanning at least 2 local trees).

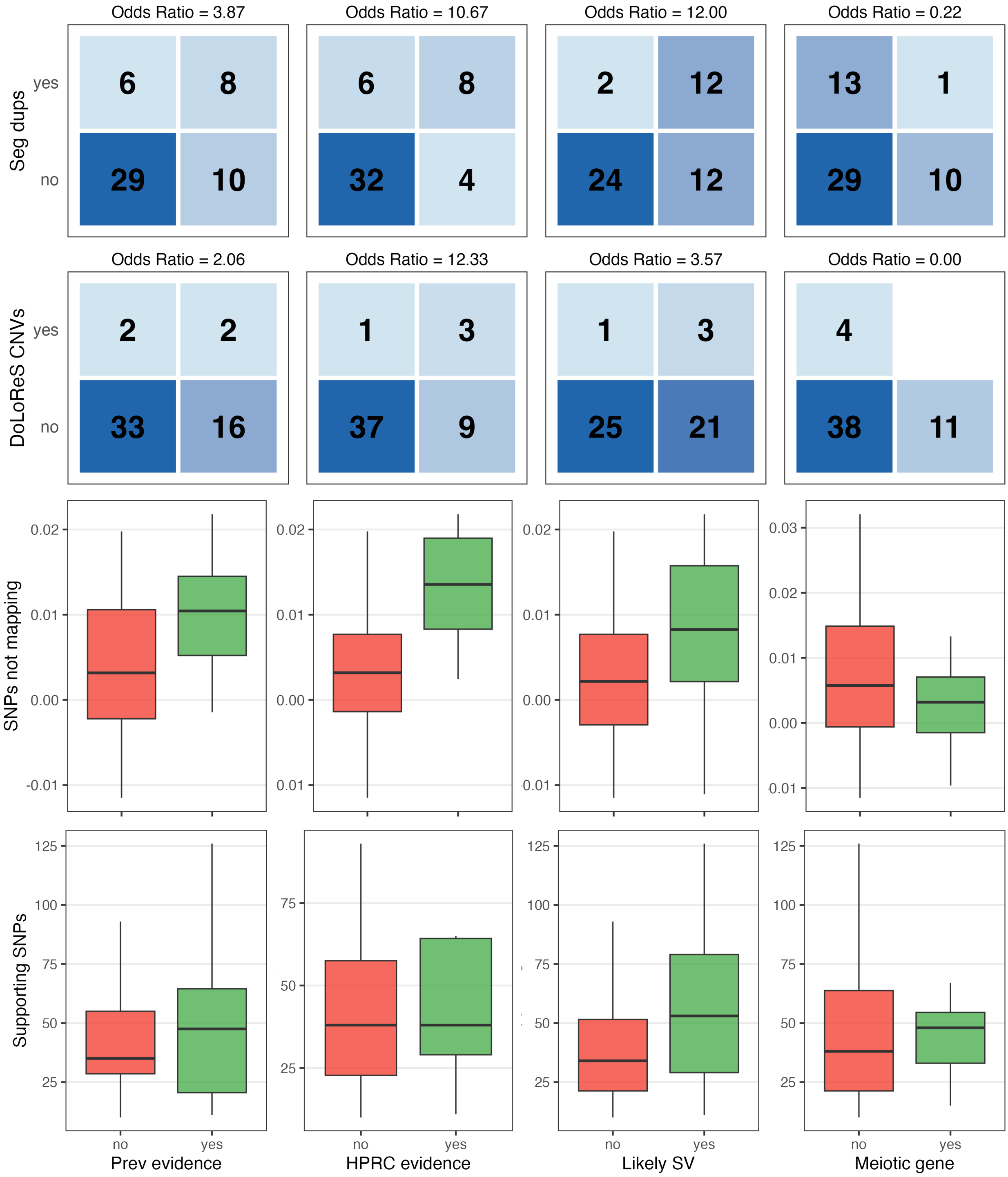

Figure S21: Some of the diagnostics that can be used to classify nature of recombination suppression in detected regions (given in full in Supplementary Table S1). Rows show the presence of direct or inverted segmental duplications (row 1), the detection of CNVs in the region by DoLoReS (row 2), the percentage point difference between the proportion of SNPs within the region that do not uniquely map to a branch of the local tree and the chromosome average proportion (row 3), the number of SNPs supporting the top significant clade (row 4). Columns show whether there is prior evidence from the literature for the detected region (column 1), whether our analysis of HPRC data shows evidence of SV (column 2), whether our assessment of the evidence toegther with other information (such as analysis of reads) points to the presence of an SV (column 3), and whether the region appears to exactly span a gene expressed in meiosis (column 4).

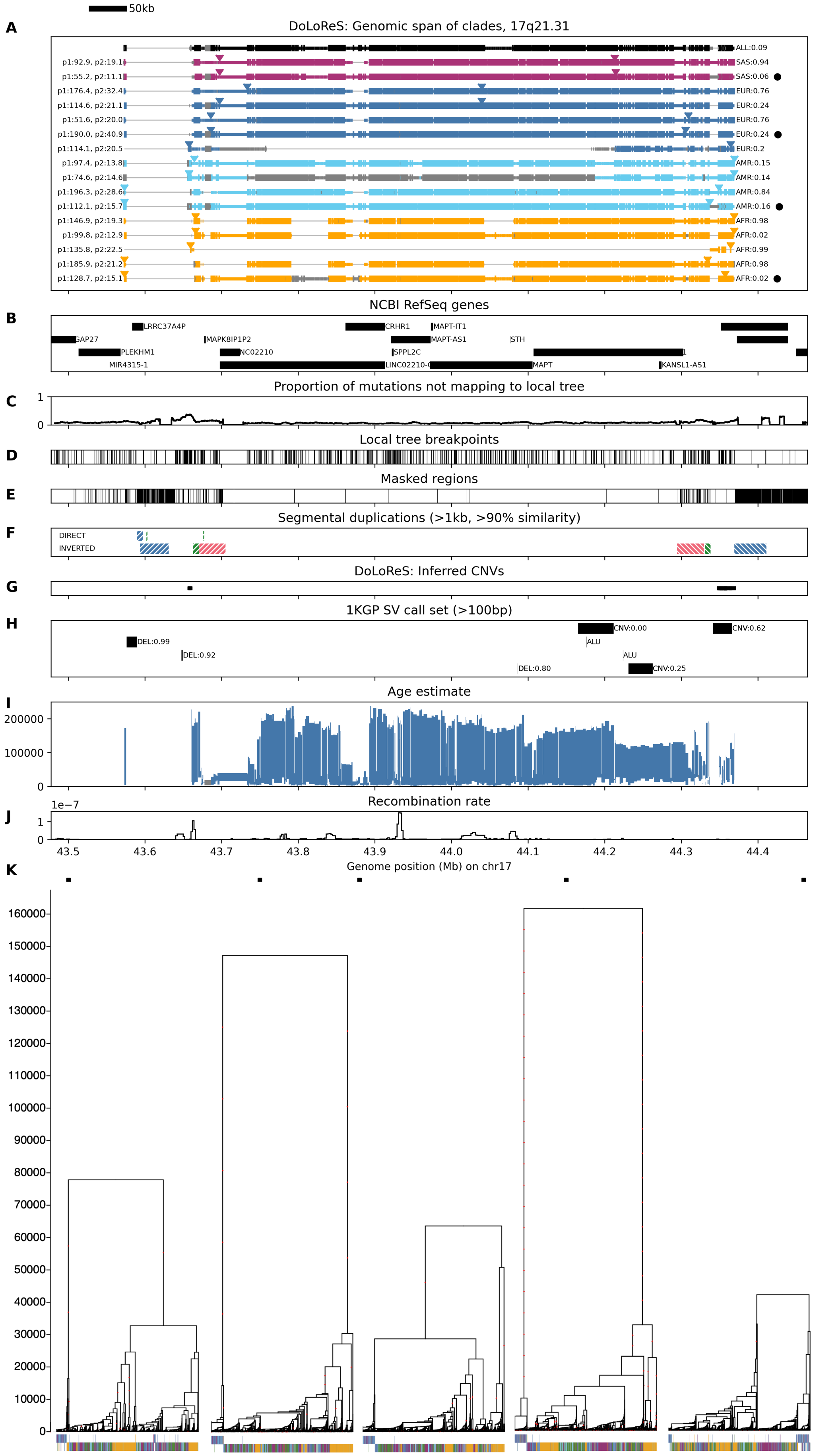

Figure S22: See caption of Figure 9 (main text). Age is estimated using the ARG subsetted to European populations.

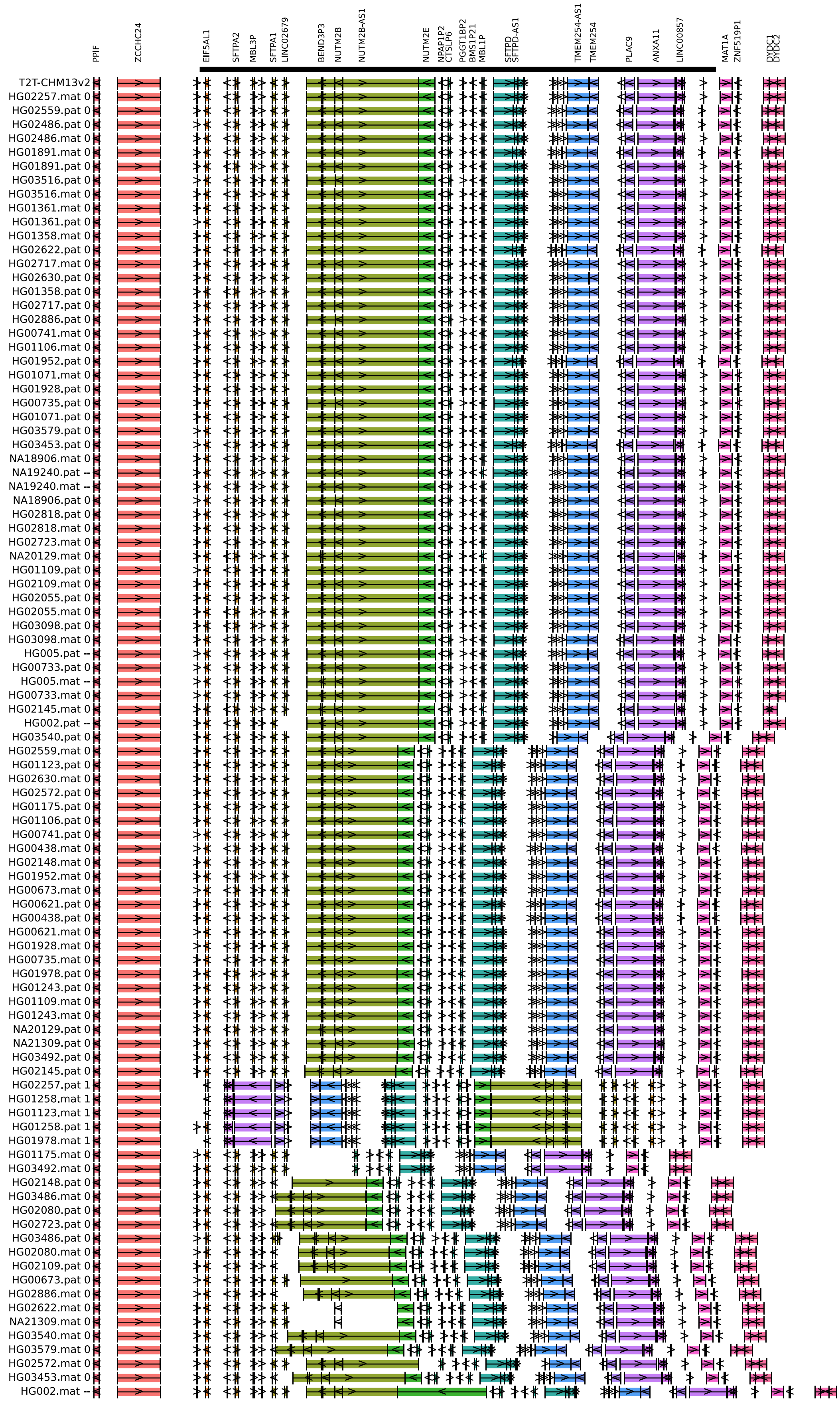

Figure S23: 10q22.3 inversion region for HPRC data and T2T-CHM13 reference. Each row is a sequence, labelled by the individual ID, whether it corresponds to the maternal (mat) or paternal (pat) haplotype, and the predicted inversion status (0 for non-carrier, 1 for carrier). Genes are coloured uniquely, from orange to purple left-to-right for the un-inverted (ancestral) orientation. Black bar shows predicted span of inversion.

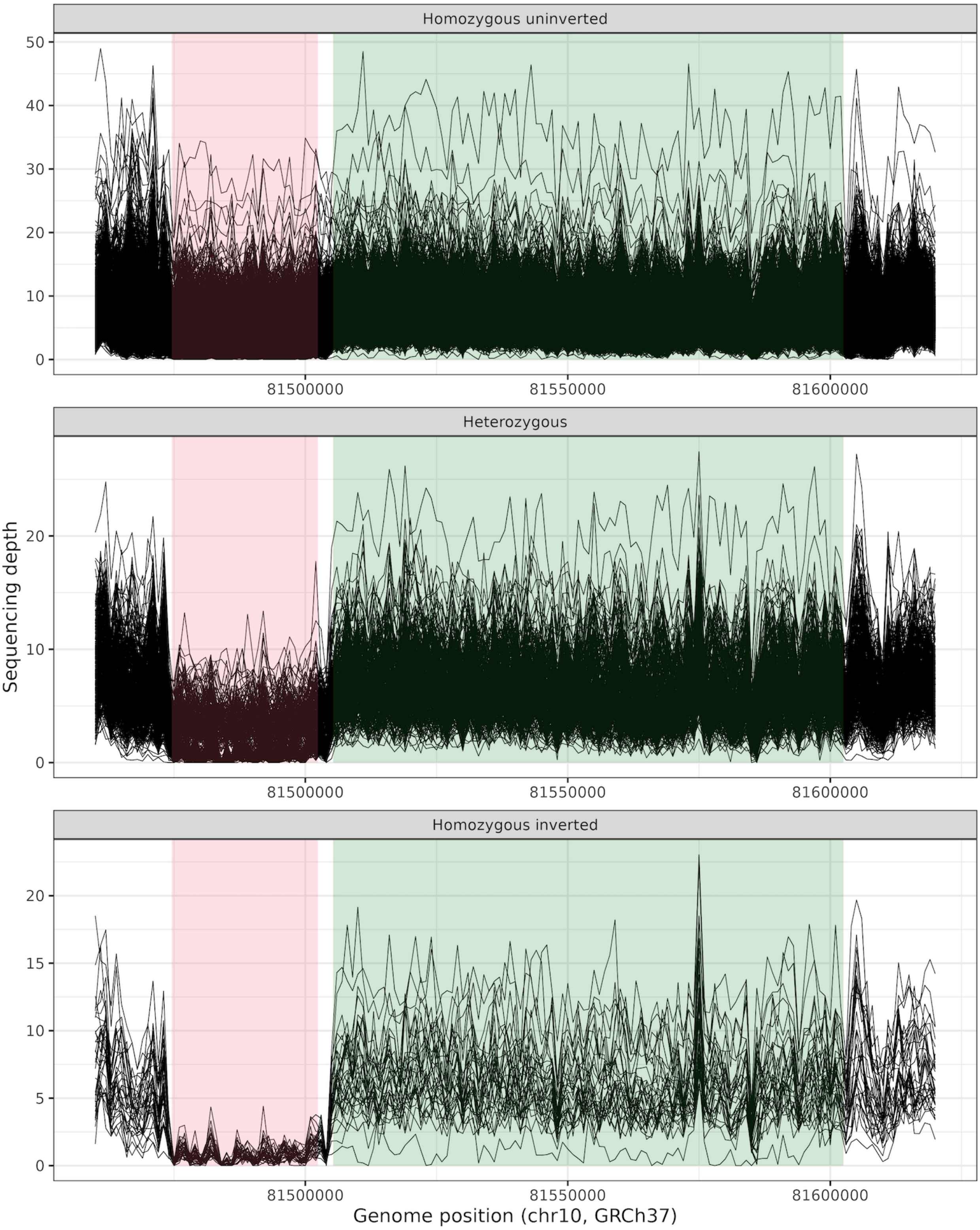

Figure S24: Sequencing read coverage on 10q22.3 around CNV1 (positions shown in red) and CNV2 (positions shown in green). Average coverage calculated in windows of 1000kb (each line corresponds to one individual) using 1KGP (Phase 3) low-coverage WGS GRCh37 data.

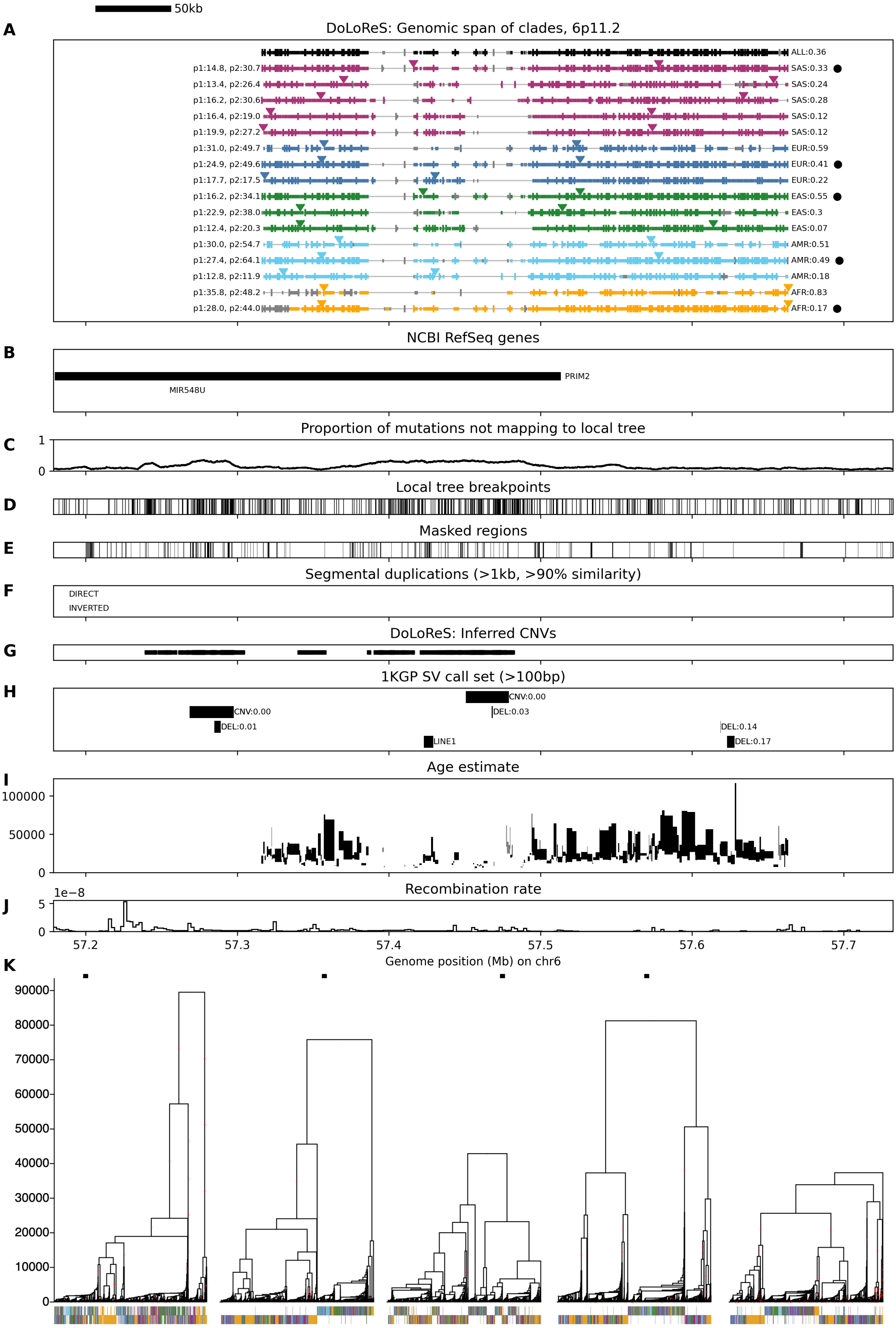

Figure S25: See caption of Figure 9 (main text). Age is estimated using the ARG for all populations.

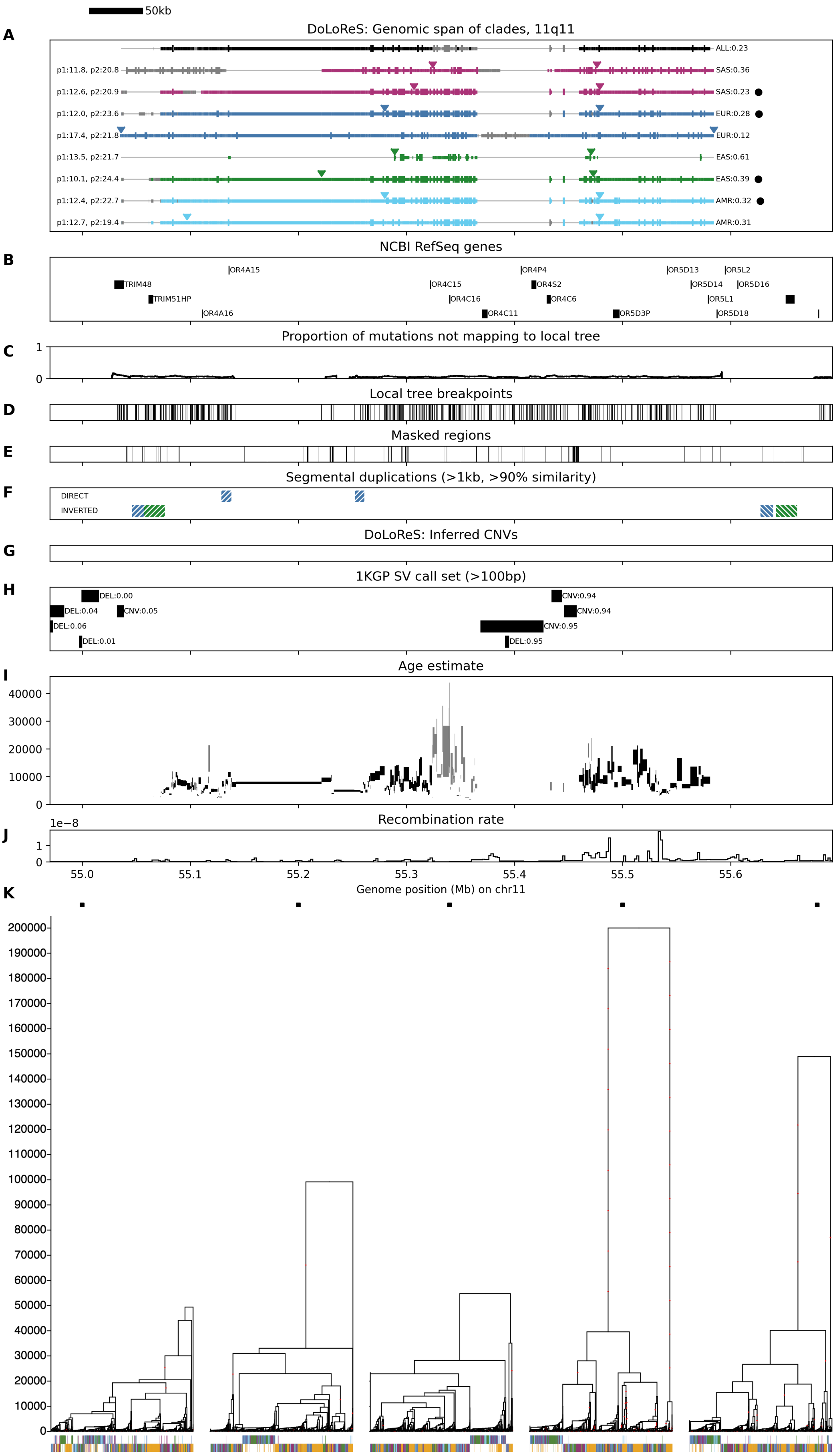

Figure S26: See caption of Figure 9 (main text). Age is estimated using the ARG for all populations.

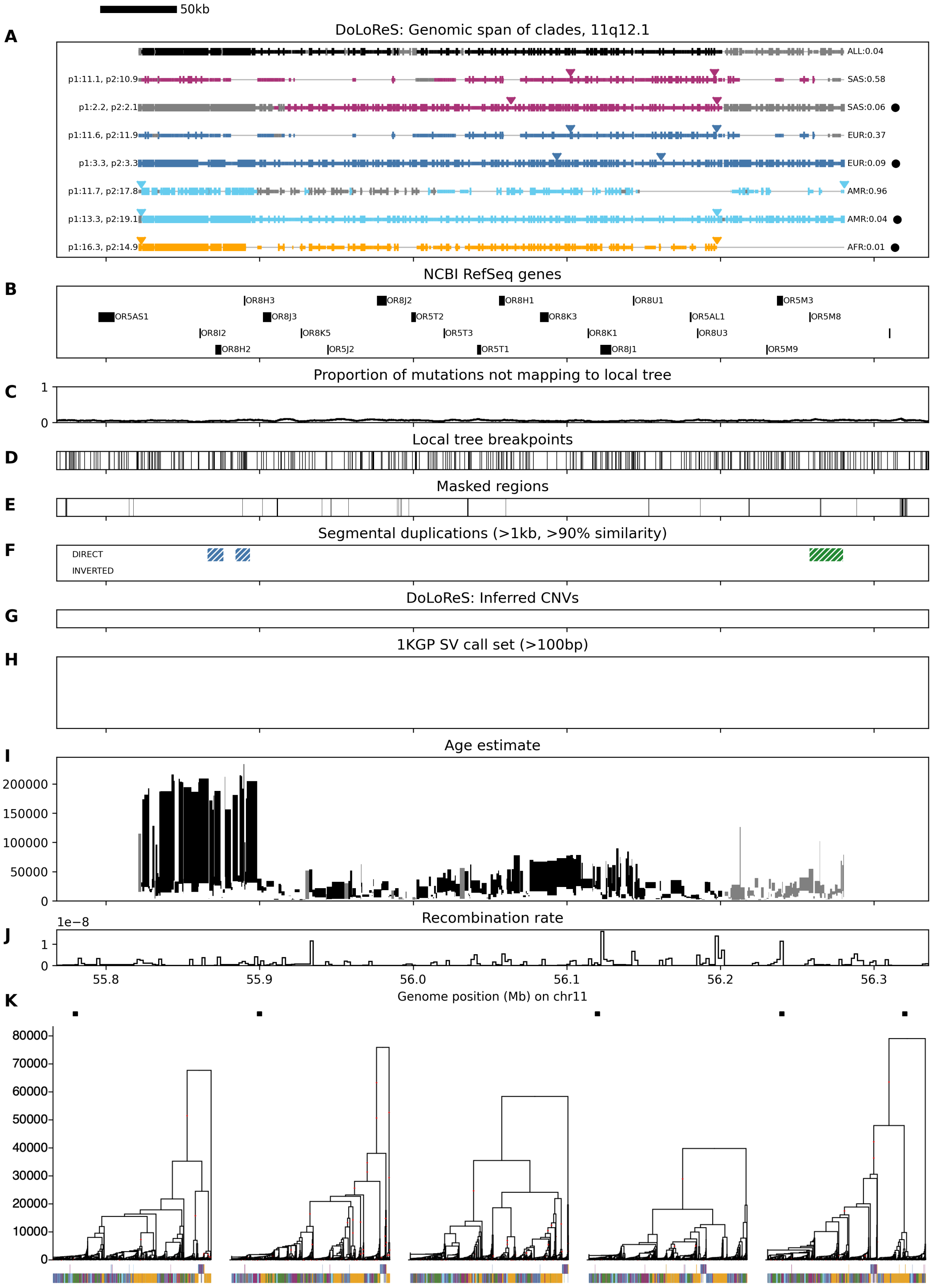

Figure S27: See caption of Figure 9 (main text). Age is estimated using the ARG for all populations.

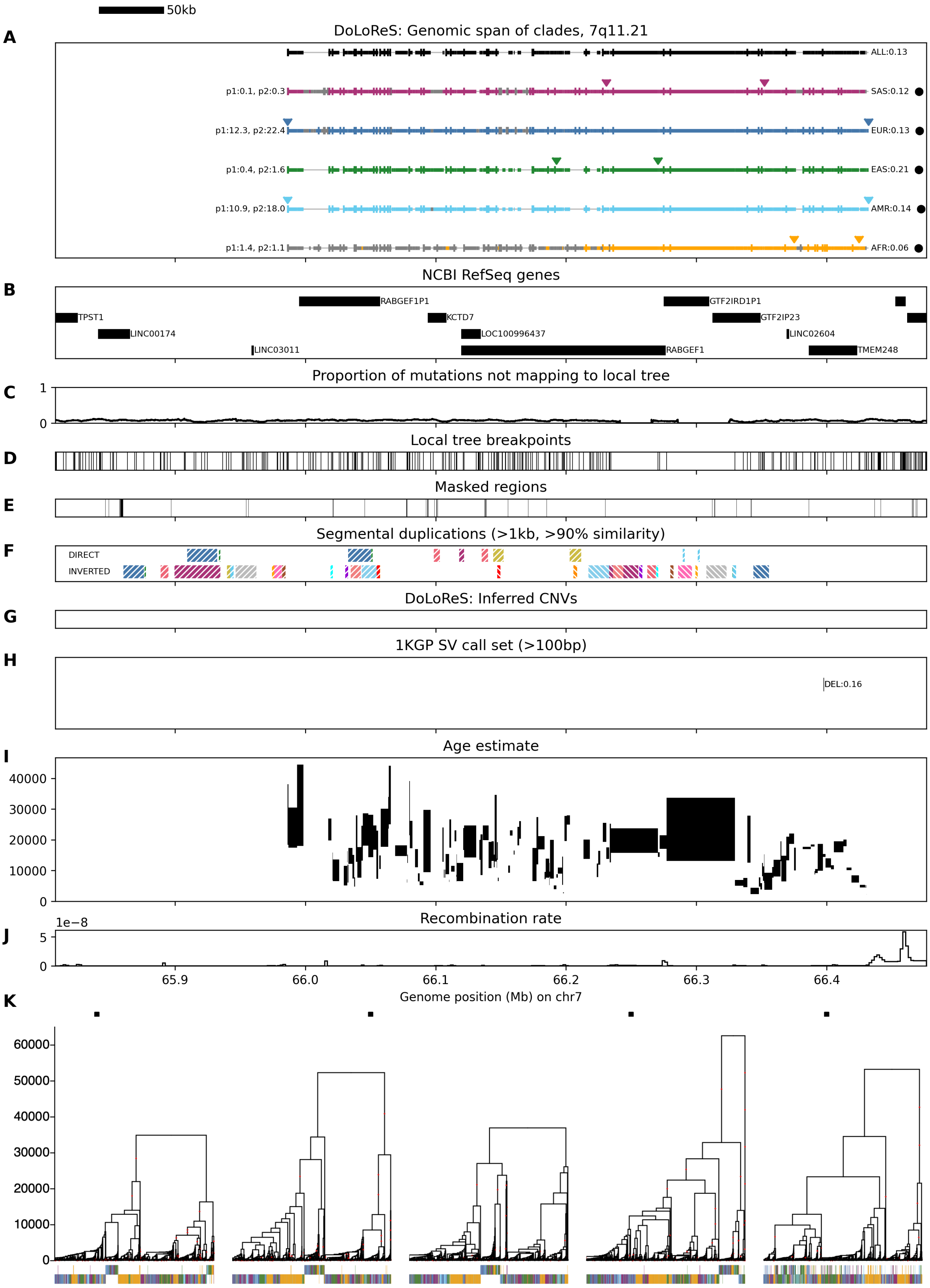

Figure S28: See caption of Figure 9 (main text). Age is estimated using the ARG for all populations.
